# Correcting for experiment-specific variability in expression compendia can remove underlying signals

**DOI:** 10.1093/gigascience/giaa117

**Published:** 2020-11-03

**Authors:** Alexandra J Lee, YoSon Park, Georgia Doing, Deborah A Hogan, Casey S Greene

**Affiliations:** Genomics and Computational Biology Graduate Program, University of Pennsylvania, 3400 Civic Center Blvd, Philadelphia, PA, 19104, USA; Department of Systems Pharmacology and Translational Therapeutics, University of Pennsylvania, 3400 Civic Center Blvd, Philadelphia, PA, 19104, USA; Department of Systems Pharmacology and Translational Therapeutics, University of Pennsylvania, 3400 Civic Center Blvd, Philadelphia, PA, 19104, USA; Department of Microbiology and Immunology, Geisel School of Medicine, Dartmouth, 1 Rope Ferry Rd, Hanover, NH, 03755, USA; Department of Microbiology and Immunology, Geisel School of Medicine, Dartmouth, 1 Rope Ferry Rd, Hanover, NH, 03755, USA; Department of Systems Pharmacology and Translational Therapeutics, University of Pennsylvania, 3400 Civic Center Blvd, Philadelphia, PA, 19104, USA; Childhood Cancer Data Lab, Alex's Lemonade Stand Foundation, 1429 Walnut St, Floor 10, Philadelphia, PA, 19102 USA

## Abstract

**Motivation:**

In the past two decades, scientists in different laboratories have assayed gene expression from millions of samples. These experiments can be combined into compendia and analyzed collectively to extract novel biological patterns. Technical variability, or "batch effects," may result from combining samples collected and processed at different times and in different settings. Such variability may distort our ability to extract true underlying biological patterns. As more integrative analysis methods arise and data collections get bigger, we must determine how technical variability affects our ability to detect desired patterns when many experiments are combined.

**Objective:**

We sought to determine the extent to which an underlying signal was masked by technical variability by simulating compendia comprising data aggregated across multiple experiments.

**Method:**

We developed a generative multi-layer neural network to simulate compendia of gene expression experiments from large-scale microbial and human datasets. We compared simulated compendia before and after introducing varying numbers of sources of undesired variability.

**Results:**

The signal from a baseline compendium was obscured when the number of added sources of variability was small. Applying statistical correction methods rescued the underlying signal in these cases. However, as the number of sources of variability increased, it became easier to detect the original signal even without correction. In fact, statistical correction reduced our power to detect the underlying signal.

**Conclusion:**

When combining a modest number of experiments, it is best to correct for experiment-specific noise. However, when many experiments are combined, statistical correction reduces our ability to extract underlying patterns.

## Introduction

Over the past two decades, unprecedented amounts of transcriptome-wide gene expression profiling data have been generated. Most of these datasets are shared on public platforms for the research community [1]. Researchers are now combining samples across different experiments to form compendia, and analyzing these compendia is revealing new biology [2–6]. It is well understood that technical sources of variability pervade large-scale data analysis such as transcriptome-wide expression profiling studies [7–10]. Numerous methods have been designed to correct for various types of effects [7, 11–13]. Despite the prevalence of technical sources of variability, researchers have successfully extracted biological patterns from multi-experiment compendia without applying correction methods [2–5, 14]. To determine the basis of these seemingly contradictory results, we examined the extent to which underlying statistical structure can be extracted from compendium-style datasets in the presence of sources of undesired variability.

A number of methods have been developed to simulate transcriptome-wide expression experiments [15–18]. However, these existing approaches require defining a statistical model that describes the process by which researchers design and carry out experiments, which is often challenging to obtain. Instead, we developed an approach to simulate compendia by sampling from the low-dimensional representation produced by multi-layer generative neural networks trained on gene expression data from an existing compendium. This allowed us to simulate gene expression experiments that mimic real experimental configurations. We combined these experiments to create compendia.

Using this simulation approach, we studied how adding varying amounts of experiment-specific noise affects our ability to detect underlying patterns in the gene expression compendia. This topic is becoming pressing as more large-scale expression compendia are becoming available. We found that prior reports of pervasive technical noise and analyses that succeed without correcting for it are, in fact, consistent. In settings with relatively few experiment-specific sources of undesired variation, the added noise substantially alters the structure of the data. In these settings, statistical correction produces a data representation that better captures the original variability in the data. On the other hand, when the number of experiment-specific sources of undesired variability is large, attempting to correct for these sources does more harm than good.

## Results

We characterized publicly available data compendia using refine.bio [19], a meta-repository that integrates data from multiple different repositories. We found that experiments typically contained hundreds to thousands of samples in most widely studied organisms (Table 1). These samples were derived from hundreds to thousands of experiments, and the most common experimental designs had relatively few samples (medians in the range 5–12). We compared compendia from refine.bio to two readily available compendia, recount2 and a compendium for *P. aeruginosa*, that have been used for compendium-wide analyses [2, 3, 6]. The compendia that have been successfully used in prior work [2, 3, 6] have similar median numbers of samples per experiment (recount2 = 4, *P. aeruginosa* = 6) to the present publicly available data.

**Table 1:** Statistics for the 10 largest transcriptomic compendia found in refine.bio

Species	No. experiments	No. samples	
		Median	Total
*Homo sapiens*	15,440	12	571,862
*Mus musculus*	13,224	10	296,829
*Arabidopsis thaliana*	1,627	9	24,855
*Rattus norvegicus*	1,368	12	38,530
*Drosophila melanogaster*	853	9	17,836
*Saccharomyces cerevisiae*	627	12	12,972
*Danio rerio*	546	9.5	28,518
*Caenorhabditis elegans*	375	10	7,953
*Sus scrofa*	280	12	6,063
*Zea mays*	274	5	3,458

The refine.bio meta-repository contains publicly available expression data from the SRA [20], Gene Expression Omnibus (GEO) [21], and ArrayExpress [22]. Public data usually have only a modest number of samples per experiment, although many samples are available in aggregate.

### Constructing a generative model for gene expression samples

**Figure 1:**
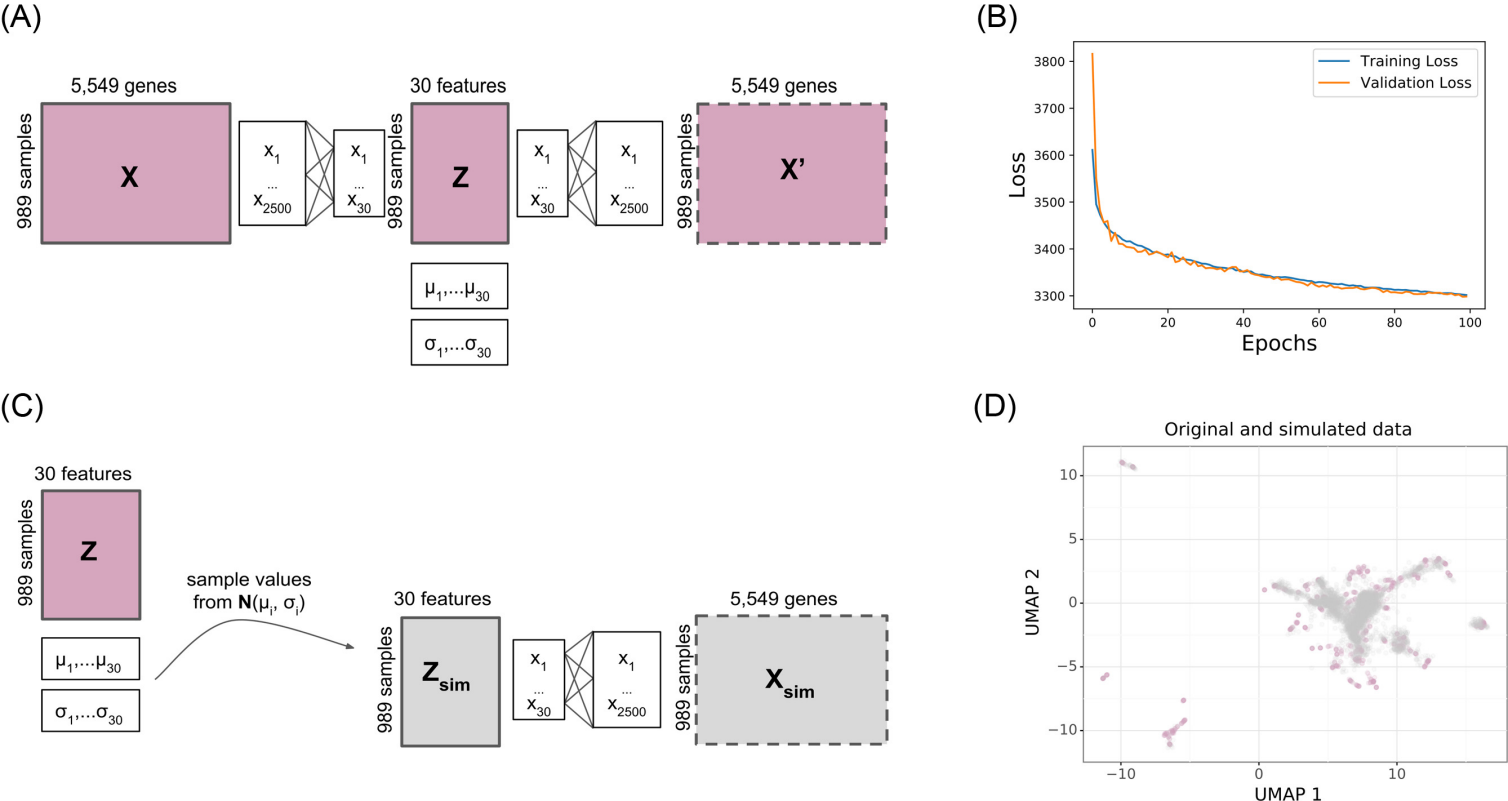
Simulating gene expression data using VAE. (A) Architecture of the VAE, where the input data get compressed into an intermediate layer of 2,500 features and then into a hidden layer of 30 latent features. Each latent feature follows a normal distribution with mean µ and variance σ. The input dimensions of the *P. aeruginosa* dataset are shown here as an example (989 samples, 5,549 genes). The same architecture is used to train the recount2 dataset except the input has 896 samples and 58,037 genes. (B) Validation loss plotted per epoch during training using the *P. aeruginosa* compendium. (C) Workflow to simulate gene expression samples from a compendium model, where new samples are generated by sampling from the latent space distribution. (D) UMAP projection of *P. aeruginosa* gene expression data from the real dataset (pink) and the simulated compendium using the workflow in C (grey).

We developed an approach to simulate new gene expression compendia using generative multi-layer neural networks. Specifically, we trained a variational autoencoder (VAE) [23], which comprised an encoder and decoder neural network. The encoder neural network compressed the input data through two layers into a low-dimensional representation, and the decoder neural network expanded the dimensionality back to the original input size. The VAE learned a low-dimensional representation that can reconstruct the original input data. Simultaneously, the VAE optimized the lowest dimensional representation to follow a normal distribution (Fig.   1A). This normal distribution constraint, which distinguishes VAEs from other types of autoencoders, allowed us to generate variations of the input data by sampling from a continuous latent space [23].

We trained VAEs for each compendium: recount2 (896 samples with 58,037 genes) and *P. aeruginosa* (989 samples with 5,549 genes). We evaluated the training and validation set losses at each epoch, which stabilized after ∼100 epochs (Fig. 1B). We observed a similar stabilization after 40 epochs for recount2 (Fig. S1A). We simulated new genome-wide gene expression data by sampling from the latent space of the VAE using a normal distribution (Fig. 1C). We used UMAP [24] to visualize the structure of the original and simulated data and found that the simulated data generally fell near original data for both compendia (Fig. 1D; Supplementary Fig. S1B).

### Simulating gene expression compendia with synthetic samples

**Figure 2:**
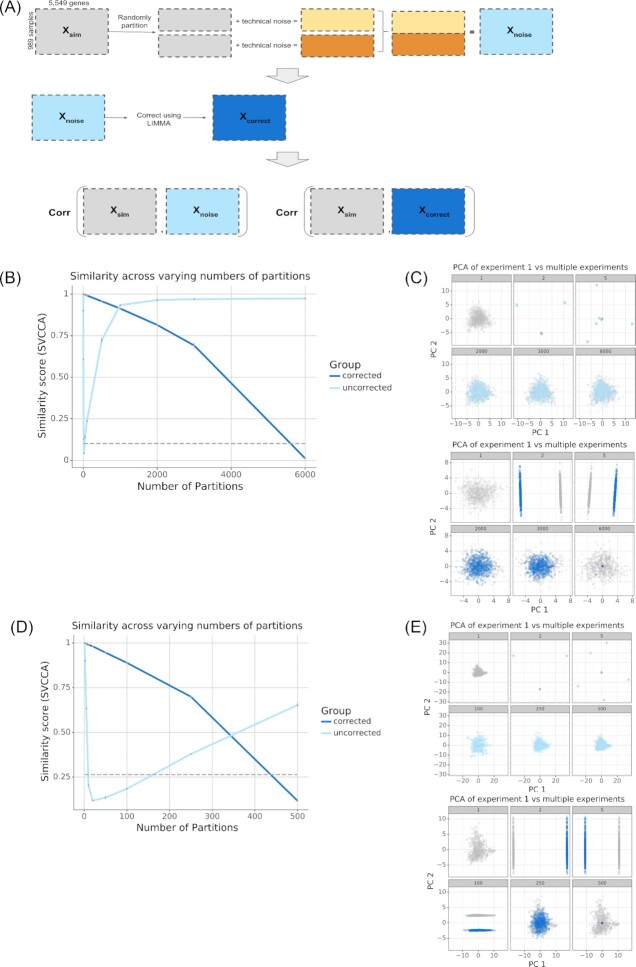
Results of simulating compendia. (A) Workflow describing how experiment-specific noise was added to the simulated compendia and how the noisy simulated compendia were evaluated for similarity compared to the unpartitioned simulated compendia. (B, D) SVCCA curve measuring the similarity between a compendium without noise vs a compendium with noise (light blue), compendium with noise corrected for (dark blue). As a negative control, we used the similarity between the gene expression pattern of the compendium without noise with a permuted compendium, where the genes were shuffled (dashed grey line) to destroy any meaningful structure in the data. (C, E) Subsampled gene expression data (500 samples per compendium) projected onto the first 2 principal components showing the overlap in structure between the compendium without noise (gray) vs the compendium with noise (light blue), compendium with noise corrected for (dark blue).

We designed a simulation study to assess the extent to which artifactual noise associated with individual partitions of a large compendium affects the structure of the overall compendium. Our simulation is akin to asking, if different laboratories performing transcriptome-wide experiments randomly sampled from the available set of possible conditions, to what extent would experiment-specific biases dominate the signal of the data? First, we simulated new compendia. Then we randomly divided the samples within these compendia into partitions and added noise to each partition. Finally, we compared the simulated compendia with added noise to the unpartitioned one (Fig. 2A). Each partition represented groups of samples with shared experiment-specific noise. We evaluated the similarity before and after applying an algorithm designed to correct for technical noise in each partition—given that the added noise was linear, we used limma [25] to correct. Singular vector canonical correlation analysis (SVCCA) [26] was used to assess similarity. The SVCCA analysis measured the correlation between the distribution of gene expression in the compendia without noise compared to the distribution in the compendia with multiple sources of technical variance.

We performed a study with this design using the VAE trained from the *P. aeruginosa* compendium. We simulated a *P. aeruginosa* compendium with 6,000 samples for [1, 2, 5, 10, 20, 50, 100, 500, 1,000, 2,000, 3,000, 6,000] partitions. We found that adding technical variance to partitions always reduced the similarity between the simulated data without partitions and the partitioned simulated data. However, the nature of the change in similarity differed substantially between the partitioned compendia before and after the correction step (Fig. 2B). With the correction step (dark blue line) similarity decreased throughout the range of the study, eventually reaching the same level as the permuted data (dashed grey line). Without the correction step (light blue line), similarity decreased immediately to the random level and then recovered throughout the rest of the tested range. We visualized the simulated data on the top 2 principal components from the original data (Fig. 2C, grey points). The corrected (Fig. 2C, dark blue) and uncorrected (Fig. 2C, light blue) data at various numbers of partitions revealed that the correction step removes both wanted and unwanted variability, eventually removing all variability in the data. Without correction, the data were initially dramatically transformed. However, as the number of partitions grows very large the effect on the structure of the data was diminished.

To determine whether this correction removing signal was a more general property of such compendia, we repeated the same simulation study using a VAE trained on a recount2 compendium. Because recount2 is a compendium composed of human RNA-seq samples, it is generated using a different technology and consists of assays of a very different organism. We simulated a compendium with 500 samples for [1, 2, 5, 10, 20, 50, 100, 250, 500] partitions. The results with recount2 mirrored our findings with the *P. aeruginosa* compendium. The correction step initially retained more similarity, but performance crossed over and by 500 partitions the uncorrected data were more similar to the unpartitioned simulated compendium (Fig. 2D). Visualizing the top principal components, again, revealed that correction restored the structure of the original data with few partitions, but with many partitions the structure was better retained without correction (Fig. 2E). Additionally, the same trends were observed when we varied the magnitude of the noise added (Fig. S2) or used a different noise correction method, such as COMBAT [12] (Supplementary Fig. S3). In general, there exists some minimum number of experiment-specific sources of noise that determines the effectiveness of applying noise correction to these multi-experiment compendia.

### A generative model for gene expression experiments

**Figure 3:**
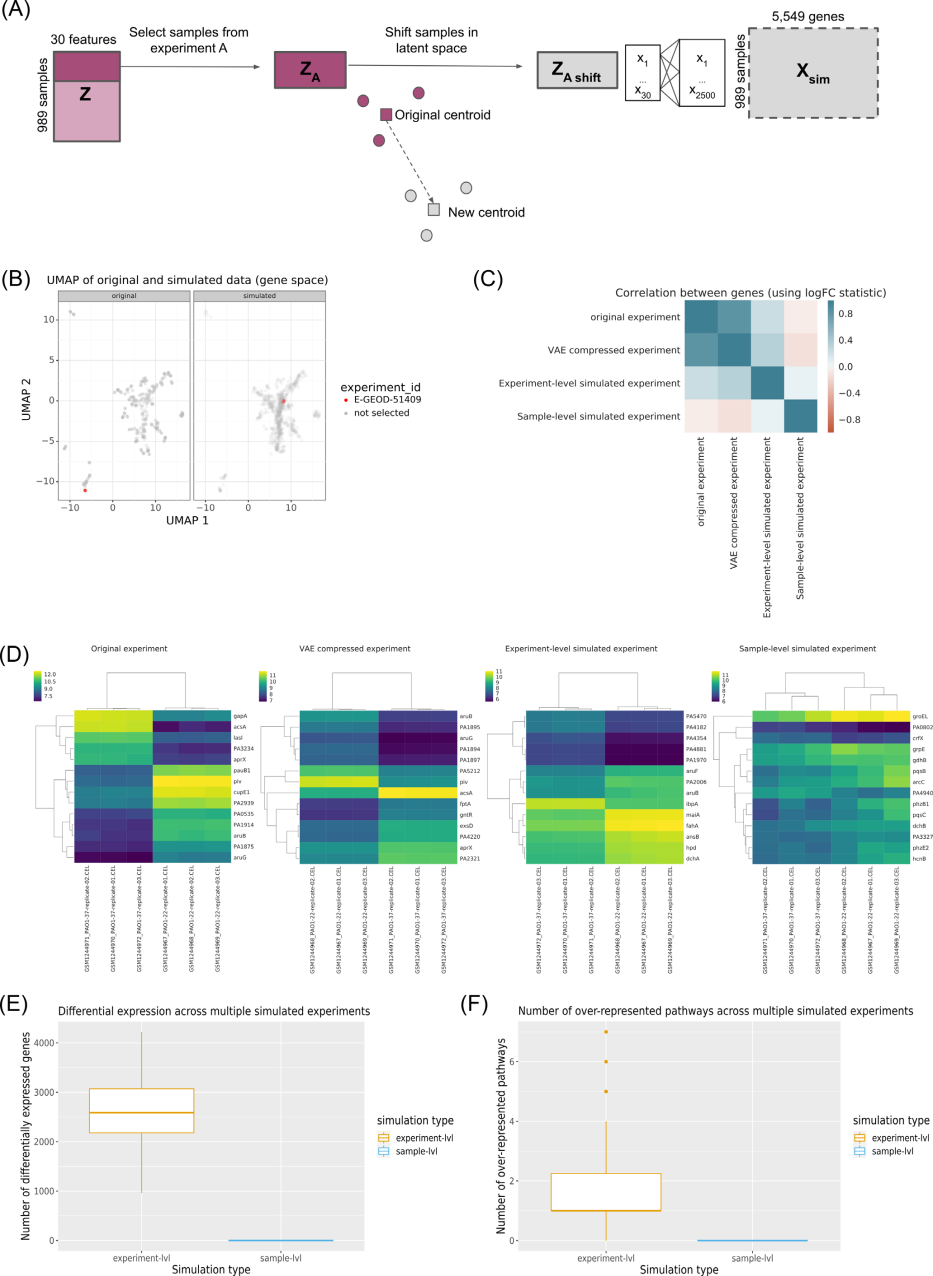
Simulating gene expression compendia by experiment. (A) Workflow to simulate gene expression per experiment. (B) UMAP projection of *P. aeruginosa* gene expression data highlighting a single experiment, E-GEOD-51409 (red), in the original dataset (left) and the simulated dataset (right), which was subsampled to 1,000 samples. (C) Correlation matrix showing the correlation between genes, based on their logFC, in the original experiment (E-GEOD-51409) vs VAE compressed experiment; original experiment vs simulated experiment using the original experiment as a template (experiment-level simulated); original vs random simulated (sample-level simulated) experiment. (D) Differential expression analysis of experiment E-GEOD-51409 (left), VAE-compressed experiment (middle-left), random simulated experiment (middle-right), and simulated experiment using the original experiment as a template (right). (E) Number of differentially expressed genes identified across 100 simulated experiments generated using experiment-level simulation and sample-level simulation. (F) Number of enriched pathways identified across 100 simulated experiments generated using experiment-level simulation and sample-level simulation. Circles denote outliers.logFC: log fold-change.

We randomly selected samples from the range of all possible samples in the compendium. This next simulation added another level of complexity to the model, by simulating experiments as opposed to samples to make the simulated compendia more representative of true expression data. This simulation generated synthetic experiments for which the gene expression patterns were consistent with those from the types of experiments that are used within the field. The technique that we developed uses the same underlying approach of sampling from a VAE. However, in this case we randomly selected a template experiment (E-GEOD-51409, which compared *P. aeruginosa* at 22°C and 37°C) and a vector that would move that template experiment to a new location in the gene expression space (Fig. 3A). The simulation preserved the relationship between samples within the template experiment while also shifting the activity of the samples in the latent space (Fig. 3B). Intuitively, this process maintained the relationship between samples but changed the underlying perturbation; this simulation maintained the same experimental design but is akin to studying a distinct biological process. We used this process to generate compendia of new gene expression experiments. We then examined the retention of the original differential expression signature by comparing the set of differentially expressed genes (DEGs) found in the simulated experiments (Fig. 3D). Applying only the VAE compression to the original experiment (E-GEOD-51409) generated an experiment that had the same sample grouping as the original. However, only a subset of the DEGs found in the VAE compressed experiment were also found in the original experiment. The VAE compression step added some noise to the expression signal in the original experiment, as expected, because the data were being compressed into a low-dimensional space. Overall, the correlation between the genes, based on their log_2_ fold-change values, in the original and VAE-compressed experiment was high, *R*^2^ = 0.822 (Fig. 3C). Next, we demonstrated how the original samples in an experiment and a simulated experiment, applying VAE compression and latent space translation of the E-GEOD-51409 experiment, had consistent clustering of samples (Fig. 3D original and experiment-level simulated experiment) [27]. However the sets of genes that were differentially expressed were different between the two experiments. This demonstrated that the perturbation intensity and experimental design were relatively consistent in gene expression space, even though the nature of the perturbation differed. The correlation between genes in the original and the experiment-level experiment was lower, *R*^2^ = 0.230, because it represented a unique experiment. The residual similarity was likely due to commonly differentially expressed genes that have been observed previously [28, 29]. Finally, as a control, we demonstrated that the original experiment structure was not well preserved using the random sampling approach (Fig. 3D, sample-level simulated experiment). The correlation between genes in the original and sample-level experiment was non-existent, *R*^2^ = −0.055, because experiment structure was not accounted for in the sample-level simulation.

In general, the numbers of DEGs found in the experiment-preserving simulated experiments (78 DEGs in VAE compressed, 14 DEGs in experiment-level) were lower compared with the original experiment (505 DEGs). This was because the simulated experiments had a lower variance compared with the original experiment. This reduced variance was due to the normality assumption made by the VAE, which compressed the latent space data representation [23]. However, the clustering of samples was conserved between the simulated and original experiments and this was also observed in the additional template experiments with more complex experimental setups (Supplementary Fig. S4). Given the fact that we preserved the association between samples and experiments in this new experiment-level simulation, we expected simulated experiments to preserve the correlation in expression of genes that are in the same pathway. In our previous example, the simulated experiment generated using the original E-GEOD-51409 as a template (i.e., experiment-level, Fig. 3A) identified 14 DEGs (Fig. 3D). In contrast, the simulated experiment generated by random sampling (i.e., sample-level, Fig. 1C) did not identify any DEGs; the median log_2_ fold-change was 0.08. Furthermore, simulating 100 new experiments using E-GEOD-51409 as a template identified a median of 2,588 DEGs compared to simulated experiments generated by random sampling, which identified a median of 0 DEGs (Fig. 3E). Additionally, the median number of enriched KEGG pathways was 1 using the template-shifting approach compared to 0 using the random sampling approach (Fig. 3F). Overall, this new simulation approach seemed to generate a compendium of more realistic experiments with underlying biology (see examples of the significantly enriched pathways in Table 2). The top over-represented pathway was the ribosome pathway, which is likely a commonly altered pathway found in many experiments regardless of experiment type, similar to the findings from human array experiments in Powers et. al. and Crow et al. [28, 29]. The remaining pathways found in the original experiment were related to metabolism, which is consistent with the finding from the original publication [27]. The simulated experiment was particularly enriched in sulfur metabolism and ABC transporters, which is consistent with an experiment that found upregulation of transport systems in response to sulfate limitations [30]. Overall, in accordance with real gene expression experiments, the new simulated experiments contain related groups of enriched pathways that reflect the specific hypotheses being tested. These results demonstrate the use of a VAE as a hypothesis-generating tool. We can now simulate new experiments to study the response of *P. aeruginosa* exposure to untested conditions.

**Table 2:** Enriched pathways found in the original E-GEOD-51409 experiment and the pseudo-experiment generated using the experiment-level simulation

Original	Adjusted *P*-value
Pae03010: Ribosome	2.966E−11
Pae00500: Starch and sucrose metabolism	1.512E−03
Pae01200: Carbon metabolism	4.466E−03
Pae00640: Propanoate metabolism	1.954E−03
Pae03010: Ribosome	7.96E−07
Pae02010: ABC transporters	4.009E−03
Pae00920: Sulfur metabolism	1.576E−02

### Simulating gene expression compendia with synthetic experiments

**Figure 4:**
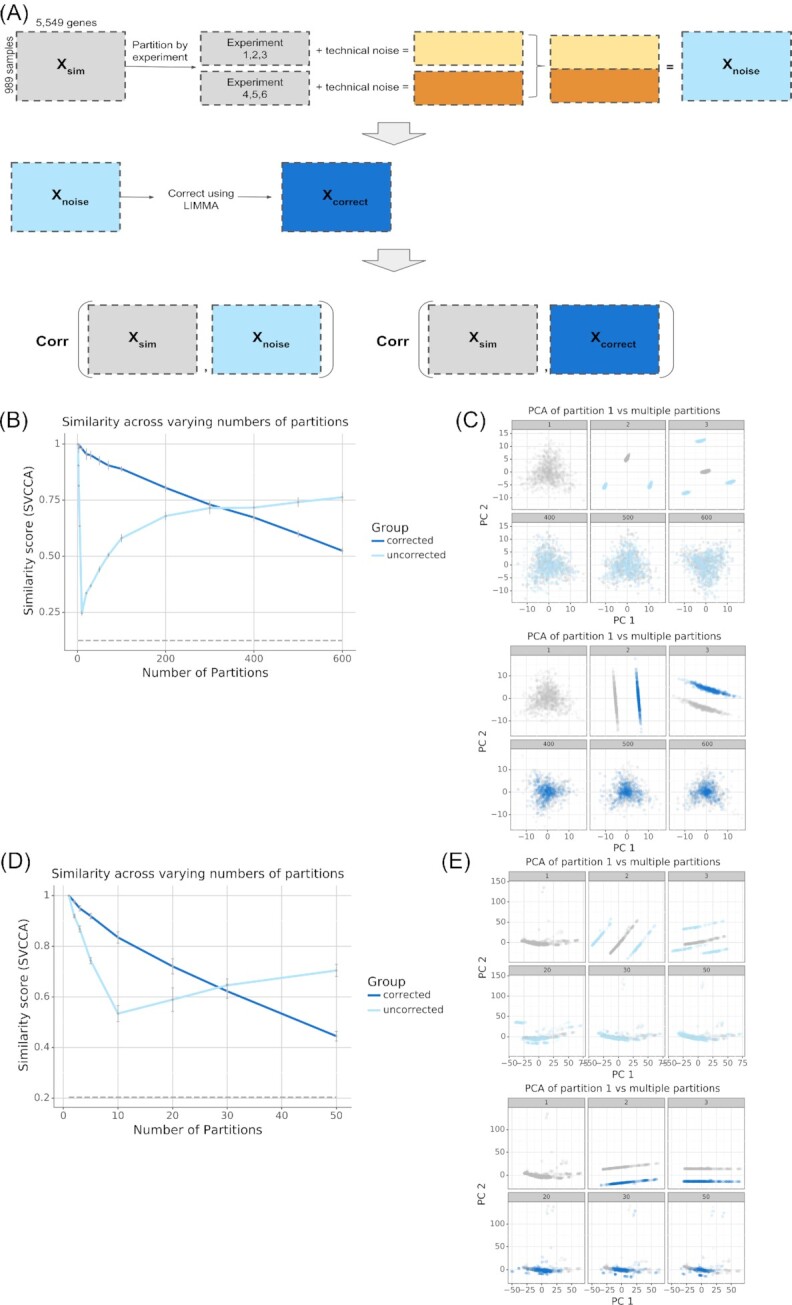
Results of simulating compendia composed of gene expression experiments. (A) Workflow describing how experiment-specific noise was added to the simulated compendia and how the noisy simulated compendia were evaluated for similarity compared to the unpartitioned simulated compendia. (B, D) SVCCA curve measuring the similarity between a compendium without noise vs a compendium with noise (light blue), and compendium with noise corrected for (dark blue). As a negative control, we used the similarity between the gene expression pattern of the compendium without noise with a permuted compendium, where the genes were shuffled (dashed grey line) to destroy any meaningful structure in the data. (C, E) Subsampled gene expression data (500 samples per compendium) projected onto the first 2 principal components showing the overlap in structure between the compendium without noise (gray) vs the compendium with noise (light blue), and compendium with noise corrected for (dark blue). PCA: principal component analysis.

We used our method to simulate new experiments that followed existing patterns to examine the patterns from generic partitions (Fig. 4A). We simulated 600 experiments using the *P. aeruginosa* compendium. We divided these experiments into [1, 2, 3, 5, 10, 20, 30, 50, 70, 100, 200, 300, 400, 500, 600] partitions. These partitions represented groupings of experiments with shared noise, such as experiments from the same laboratory or experiments with the same experimental design. Each partition contained technical sources of variance within and between experiments. Results with simulated experiments were similar to those from arbitrarily partitioned samples. We observed a monotonic loss of similarity after the correction step as the number of partitions increased (Fig. 4B). Visualizing the top principal components revealed that statistical correction initially better recapitulated the overall structure of the data but that similarity decreased with many partitions (Fig. 4C, dark blue). Without statistical correction there was a larger initial decrease in similarity but a later recovery (Fig. 4B) and visualizing the top principal components recapitulated this finding (Fig. 4C, light blue). We performed analogous experiments using the recount2 VAE and 50 simulated experiments with [1, 2, 5, 10, 20, 30, 50] partitions. We observed consistent results with this dataset using both SVCCA similarity (Fig. 4D) and visual inspection of the top principal components (Fig. 4E).

One caveat in the design of the previous analysis is that the effect of the number of partitions was confounded by the number of experiments per partition. For example, more partitions equated to each partition having a smaller effect size because each partition had fewer experiments. To study the contribution of individual experiments in our signal detection, we performed an analysis where we held the number of experiments per partition fixed and varied the number of total experiments within a compendium (Supplementary Fig. S5A). With few experiments in a compendium, the main signal was the difference between experiments so adding noise to each experiment drove signal detection down (Supplementary Fig. S5B). Additionally, applying noise correction removed the main experiment-specific signal, as it was designed to do. With more experiments in a compendium, we gained a more global gene expression representation, where the main signal was no longer focused on the difference between experiments. Thus, adding noise to each experiment did not affect our signal detection and our similarity remained constant. However, applying noise correction will consistently remove more of our signal of interest. The results of this analysis exemplify how existing experiments can be combined and used without need for correction.

In summary, as the number of partitions or experiments increase the experiment-specific technical sources contribute less to the overall signal and the underlying patterns dominate the overall signal. When many partitions or experiments are present, even ideal statistical approaches to correct for noise over-correct and remove the underlying signal.

## Discussion

Our findings reveal that compendium-wide analyses do not always require correction for experiment-specific technical variance and that correcting for such variance may remove signal. This simulation study provides an explanation for the observation that past studies [2–6] have successfully extracted biological signatures from gene expression compendia despite the presence of uncorrected experiment-specific sources of technical variability. In general, there exist compendia that contain some small number of experiment-specific sources where traditional correction methods can be effective at recovering the biological structure of interest. However, there also exist large-scale gene expression compendia where these methods may be harmful instead of helpful. The number of experiment-specific sources that determines whether to apply correction will vary depending on the size of the compendia and the magnitude and structure of the signals. Using the associated repository [31] users can customize the scripts to run the simulation experiments on their own expression data to examine the effect of a linear noise model with linear noise correction on their dataset. Although our analysis uses simplifying assumptions that preclude us from defining a specific threshold for noise correction, these simulations define a set of general properties that will guide compendium analyses moving forward. This study suggests that new large-scale datasets can be created by distributing different experiments across many different laboratories and centers as opposed to being consolidated within a single laboratory.

We introduce a new method to simulate genome-wide gene expression experiments, using existing gene expression data as starting material, which goes beyond simulating individual samples. This allows us to examine the extent to which our findings hold with realistic experimental designs. The ability to simulate gene expression experiments with a realistic structure has many potential legitimate uses: pre-training for machine learning models, providing synthetic test data for software, and other such applications. Additionally, this simulation technique can be used to explore hypothetical experiments that have not been previously performed and generate hypotheses. However, such approaches could also be used by nefarious actors to generate synthetic data for publications. Forensic tools that detect synthetic genome-wide data may be needed to combat potential fraudulent uses.

Our study has several limitations. We assume a certain noise model that differs between experiments. However, the sources of real noise are multifaceted and any such assumption will necessarily be an oversimplification, although such assumptions are not uncommon [10, 12, 32]. By selecting a specific noise model and using an ideal noise-removal step, we provide a best-case scenario for artifact removal. While any simulation study will necessarily make simplifying assumptions, this work is the first to use deep generative models as part of a simulation study to probe the long-standing assumption that correcting for technical variability is necessary for analyses that span multiple experiments. Our findings reveal that in settings with hundreds or thousands of experiments, correcting for experiment-specific effects can harm performance and that it can be best to forgo statistical correction. Adjusting the choices of normalization, noise magnitude, and noise patterns will result in different selections of the precise cross-over point where it becomes beneficial to perform correction. With this design, we do not expect to estimate exactly where this precise cross-over point is. Such an estimation would require a compendium where investigators systematically performed the same combination of different experiments in multiple laboratories at different times. We were unable to identify such a compendium on the scale of thousands of samples from tens to hundreds of laboratories. Thus, although our analysis necessarily includes simplifying assumptions that limit our ability to precisely define the thresholds for correction for arbitrary datasets and noise sources, it remains suitable for examining the overriding principles that govern compendium-wide analyses.

Our study has broad implications for efforts to standardize scientific processes. Centralization of large-scale data generation has the potential to reduce experiment-specific technical noise, although it comes at a cost of flexibility. Our results suggest that a highly distributed process where experiments are carried out in many different locations, with their own specific sources of technical noise, can also lead to valuable data collections.

## Methods

### *Pseudomonas aeruginosa* gene expression compendium

We downloaded a compendium of *P. aeruginosa* data that was previously used for compendium-wide analyses [2]. Previous studies identified biologically relevant processes such as oxygen deprivation [2] and phosphate starvation [3] by applying denoising autoencoders. We obtained the processed and normalized gene expression matrices from the ADAGE GitHub repository [33]. The *P. aeruginosa* dataset was previously processed by Tan et al. During processing, raw microarray data were downloaded as .cel files, rma was used to convert probe intensity values from the .cel files to log_2_ base gene expression measurements, and these gene expression values were then normalized to 0–1 range per genes.

This compendium includes measurements from 107 experiments that contain 989 samples for 5,549 genes [2]. It contains experiments that accrued between the release of the GeneChip *P. aeruginosa* genome array and the time of data freeze in 2014. Approximately 70% of the samples were from cultures of strain PAO1 and derivatives, 13% were in strain PA14 background, 0.6% were from PAK strains, and the remaining were largely clinical isolates. Of the strains, 73% were wild-type (WT) genotypes and the rest were mutants that had undergone genetic modification. Approximately 60% of the samples were grown in lysogeny broth medium while the rest were grown in Pseudomonas Isolation Agar, glucose, pyruvate, or amino acid-based media [3]. Roughly 80% were grown planktonically, 15% were grown in biofilms, and the remaining samples were *in vivo* or not annotated. Overall, this *P. aeruginosa* compendium covered a wide range of gene expression patterns including characterization of clinical isolates from cystic fibrosis infections, differences between mutant versus WT, response to antibiotic treatment, microbial interactions, and adaptation from water to gastrointestinal tract infection. Despite having 989 samples, this compendium represents the heterogeneity of *P. aeruginosa* gene expression.

### recount2 gene expression compendium

We downloaded human RNA-seq data from recount2 [34]. The dataset includes >70,000 samples collected from the SRA. It comprises >50,000 samples from different types of experiments, ∼10,000 samples from the Genotype-Tissue Expression project (GTEx v6) covering 44 types of normal tissue, and >10,000 samples from The Cancer Genome Atlas (TCGA) measuring 33 cancer types [20, 35, 36]. The recount2 authors uniformly processed and quantified these data. We downloaded data using the recount library in Bioconductor (version 1.14.0) [34]. The entire recount2 dataset is 8 TB. On the basis of the *P. aeruginosa* compendium we expected a subset of the compendium to be sufficient for this simulation, so we selected a random subset of 50 NCBI studies, which resulted in 896 samples with 58,037 genes for our simulation. Each project (imported from NCBI bioproject) is akin to an experiment in the *P. aeruginosa* compendium, and we used the term "experiment" to describe different projects to maintain consistency in this article. The downloaded recount2 dataset was in the form of raw read counts, which was normalized to produce RPKMs used in our analysis. The normalized gene expression data were then scaled to a 0–1 range per gene.

### Strategy to construct VAE: structure and hyperparameters

We designed an approach to simulate gene expression compendia with a multi-layer VAE. We built this model in Keras (version 2.1.6) with a TensorFlow back end (version 1.10.0), modifying the previously published Tybalt method [37–39]. Our architecture used each input gene as a feature. These genes were compressed to 2,500 intermediate features using a rectified linear unit (ReLU) activation function to combine weighted nodes from the previous layer. These features were encoded into 30 latent space features, also using a ReLU activation function, which were optimized via the addition of a Kullback-Leibler divergence term into the loss function (binary cross entropy) to follow a standard normal distribution. These features were then reconstructed back to the input feature dimensions using decoding layers that mirror the structure of the encoder network. We trained the VAE using 90% of the input dataset, leaving 10% as a validation set. We determined training hyperparameters by manually adjusting parameters and selecting the parameters that optimized the validation loss based on visual inspection. These were a learning rate of 0.001, a batch size of 100, warmups set to 0.01, 100 epochs for the *P. aeruginosa* compendium, and 20 epochs for the recount2 compendium. A similar assessment was performed to determine the neural network architecture. We manually inspected the validation loss using multiple different 2-layer designs (300–10, 2,500–10, 2,500–20, 2,500–30, 2,500–100, 2,500–300) and found a 2,500 layer to a 30 hidden layer VAE to be most optimal.

### Sample-based simulation

We used the VAE trained from each compendium to generate new compendia by randomly sampling from the latent space. We generated a simulated compendium containing 6,000 *P. aeruginosa* samples or 500 recount2 samples. For our first simulation, we sampled randomly—ignoring the relationship between samples within a specific experiment. We simulated experiment-specific sources of undesired variability within compendia by dividing the data into partitions and adding noise to each partition.

We divided the *P. aeruginosa* simulated compendium into [1, 2, 5, 10, 20, 50, 100, 500, 1,000, 2,000, 3,000, 6,000] partitions and divided the recount2 simulated compendium into [1, 2, 5, 10, 20, 50, 100, 250, 500] partitions. Each partition of data represented a group of samples from the same experiment or laboratory. We randomly added linear noise to each partition by generating a vector of length equal to the number of genes (5,549 *P. aeruginosa* genes and 58,037 human genes), where each value in the vector was drawn from a normal distribution with a mean of 0 and a variance of 0.2. With the 0–1 scaling, a value of 0.2 produces a relatively large difference in gene expression space (Supplementary Fig. S1). Although linear noise is an over-simplification of the types of noise that affect gene expression data, it allowed us to design an approach to optimally remove noise.

### Experiment-based simulation

For the experiment-level simulation, we developed an approach that could simulate realistic experimental structure. There was no consistent set of annotated experimental designs, so we developed a simulation method that did not depend on *a priori* knowledge of experimental design. For each synthetic experiment, we randomly sampled a “template experiment” from the set of *P. aeruginosa* or recount2 experiments. We then simulated new data that matched the template experiment by selecting a random location from the low-dimensional representation of the simulated compendia (i.e., selecting a location according to the low-dimensional distribution) and calculating the vector that connected this random location and the encoded template experiment. We then linearly shifted the template experiment in the low-dimensional latent space by adding this vector to each sample in the experiment. This process preserved the relationship between samples within the experiment but shifted the samples to a new location in the latent space. Repeating this process for each experiment allowed us to generate new simulated compendia composed of realistic experimental designs.

We divided the *P. aeruginosa* simulated compendium into [1, 2, 3, 5, 10, 20, 30, 50, 70, 100, 200, 300, 400, 500, 600] partitions and divided the recount2 simulated compendium into [1, 2, 5, 10, 20, 30, 50] partitions, where experiments are divided equally amongst the partitions. For each partition we added simulated noise as described in the previous section. Experiments within the same partition had the same noise added. Each partition represented a group of experiments generated from the same laboratory or with the same experimental design.

### Experiment-effect analysis

For this analysis we wanted to examine the effect of individual experiments on our ability to detect underlying gene expression structure. First, we used the experiment-based simulation approach to simulate *P. aeruginosa* compendia with [2, 3, 5, 10, 20, 30, 50, 70, 100, 200, 300, 400, 500, 600] experiments. Next, we divided the simulated compendium into the same number of partitions so that there was 1 experiment per partition. For each partition we added simulated noise as described in the previous section. Finally we used SVCCA to compare the noisy compendia with *X* number experiments with the unpartitioned compendia with *X* number of experiments. We also used SVCCA to compare the noise-corrected compendia with *X* experiments with the unpartitioned compendia with *X* experiments.

### Removing technical variability from noisy compendia

Our model of undesired variability was a linear signature applied separately to each partition of the data, which we considered akin to experiments or groups of experiments in a compendium of gene expression data. We used the removeBatchEffect function in the R library, limma (limma, RRID:SCR_010943) version 3.44.0, to correct for the technical variation that was artificially added to the simulated compendia [25]. Limma removes the technical noise by first fitting a linear model to describe the relationship between the input gene expression data and the experiment labels. The input expression data contain both a biological signal and technical noise component. By fitting a linear model, limma will extract the noise contribution and then subtract this from the total input expression data. This method presents a best-case scenario for removing the undesired variability in the simulated compendia because the model matches the noise pattern that we used in the simulation.

### Measuring the similarity of matched compendia

We used SSVCCA [26] to estimate similarities between different compendia. SVCCA is a method designed to compare 2 data representations [26]. Given 2 multivariate datasets, X_1_ and X_2_, the goal of SVCCA is to find the basis vectors, *w* and *s*, to maximize the correlation between w^T^X_1_ and s^T^X_2_. In other words, SVCCA attempts to find the space, defined by a set of basis vectors, such that the projection of the data onto that space is most correlated. Two datasets are considered similar if their linearly invariant correlation is high (i.e., if X_1_ is a shift or rotation of X_2_, then X_1_ and X_2_ are considered similar).

We compared the statistical structure of the gene expression, projected onto the first 10 principal components, in the baseline simulated compendia (those with only 1 experiment or partition, X_1_) versus those with multiple experiments or partitions (X_2_). Our SVCCA analysis was designed to measure the extent to which the gene expression structure of the compendia without noise was similar to the gene expression structure of the compendia with multiple sources of technical variance added, as well as those where correction has been applied. Here we use 10 principal components for computational simplicity. Selecting a different value would affect the cross-over point but not the general trends that we describe.

### A case study of differential expression in a template experiment

We compared the E-GEOD-51409 experiment [40] with 2 different simulated representations to provide a case study for experiment-based simulation. E-GEOD-51409 included *P. aeruginosa* in 2 different growth conditions. For 1 simulation, we generated random samples and randomly assigned them to conditions, which we termed the sample-simulated experiment. For the second we used the latent space transformation process described above, which we termed the experiment-simulated experiment. We used the eBayes module in the limma library to calculate differential gene expression values for each gene between the 2 different growth conditions in the real and simulated data. We built heat maps for the 14 most differentially expressed genes, where DEGs were those with false discovery rate–adjusted cut-off (using Benjamini-Hochberg correction) <0.05 and log_2_ fold-change >1, which are thresholds frequently used in practice. We selected 14 genes because there were 505, 14, and 0 DEGs found in the original experiment, experiment-simulated experiment, and sample-simulated experiment, respectively. Because 0 DEGs were found in the sample-simulated experiment, we displayed the top 14 genes sorted by adjusted *P*-value to provide a visual summary of the simulation process.

### Comparing sample-level and experiment-level simulated datasets

We simulated 100 experiments using the template E-GEOD-51409 experiment [40]. We sought to compare the sample-level and experiment-level simulation processes. We set a threshold for DEGs at a Bonferroni-corrected *P*-value cut-off of 0.05/5,549. We used the enrichKEGG module in the clusterProfiler library (clusterProfiler, RRID:SCR_016884) to conduct an over-representation analysis [41]. We used the Fisher exact test to calculate a *P*-value for over-representation of pathways in the set of DEGs. We considered pathways to be over-represented if the *q*-value was <0.02.

### Implementation and software availability

All scripts to reproduce this analysis are available the GitHub repository [31] under an open source license. The repository contains 98% Python Jupyter notebooks, 2% Python, and 0.1% R scripts. The repository's structure is separated by input dataset. "Pseudomonas/" and "Human/" directories each contain the input data in the data/input/directory. Scripts for the sample-level simulation can be found in Pseudomonas/Pseudomonas_sample_lvl_sim.ipynb for the *P. aeruginosa* compendium and Human/Human_sample_lvl_sim.ipynb for the recount2 compendium. Scripts for the experiment-level simulation can be found in Pseudomonas/Pseudomonas_experiment_lvl_sim.ipynb and Human/Human_experiment_lvl_sim.ipynb, respectively. The virtual environment was managed using Conda (version 4.6.12), and the required libraries and packages are defined in the environment.yml file. Additionally, scripts to simulate gene expression compendia using the sample-level and experiment-level approaches are available as a separate module, called ponyo, and can be installed from PyPi [42]. We describe in the Readme file how users can analyze different compendia or use different noise patterns. All simulations were run on a CPU.

## Availability of Supporting Source Code and Requirements

- Project name: Simulate Expression Compendia
- Project home page: https://github.com/greenelab/simulate-expression-compendia
- Operating systems: Mac OS, Linux
- Programming language: Python, R
- Other requirements: Git LFS
- License: BSD v3

## Availability of Supporting Data and Materials

An archival copy of the GitHub repository (including scripts and result files) is available in the *GigaScience* GigaDB repository [43].

## Additional Files

**Figure S1:** Simulating recount2 gene expression data using VAE. (A) Validation loss plotted per epoch during training. (B) UMAP projection of gene expression data from the real dataset (pink) and the simulated compendium using the workflow in Fig. 1C (grey).

**Figure S2:** Results of varying the magnitude of the experiment-specific noise added to each partition. SVCCA curve measuring the similarity between a compendium without noise vs a compendium with noise (light blue), and compendium with noise corrected for (dark blue). As a negative control, we used the similarity between the gene expression pattern of the simulated data with a single partition compared with the simulated data permuted to destroy any meaningful structure in the data. Using noise model with (A) 0.2 variance, (B) 0.05 variance with a zoomed-in view on the left, (C) 0.025 variance with a zoomed-in view on the left.

**Figure S3:** Results of simulating *P. aeruginosa* compendia using (A) sample-level simulation or (B) experiment-level simulation with COMBAT noise correction.

**Figure S4:** Clustering of 100 random gene expression profiles in original vs simulated experiments using (A) E-GEOD-21704 and (B) E-GEOD-10030 as templated.

**Figure S5:** Results of simulating compendia with fixed number of experiments. (A) Workflow describing how each compendium is designed to have a fixed number of experiments, how experiment-specific noise was added to the simulated compendia, and how the noisy simulated compendia were evaluated for similarity compared to the unpartitioned simulated compendia. (B) SVCCA curve measuring the similarity between a compendium without noise vs a compendium with noise (light blue), and compendium with noise corrected for (dark blue). As a negative control, we used the similarity between the gene expression pattern of the simulated data with a single partition compared with the simulated data that have been permuted to destroy any meaningful structure in the data. (C) Subsampled gene expression data (<500 samples per compendium) projected onto the first 2 principal components showing the overlap in structure between the compendium without noise (gray) vs the compendium with noise (light blue), and compendium with noise corrected for (dark blue).

## Abbreviations

CPU: central processing unit; DEG: differentially expressed gene; GEO: Gene Expression Omnibus; GTEx: Genotype-Tissue Expression project; KEGG: Kyoto Encyclopedia of Genes and Genomes; NCBI: National Center for Biotechnology Information; ReLU: rectified linear unit; RNA-seq: RNA sequencing; RPKM: reads per kilobase per million mapped reads; SRA: Sequence Read Archive; SVCCA: singular vector canonical correlation analysis; TCGA: The Cancer Genome Atlas; UMAP: Uniform Manifold Approximation and Projection; VAE: variational autoencoder; WT: wild type.

## Competing Interests

The authors declare that they have no competing interests.

## Funding

This work was funded in part by grants from the Cystic Fibrosis Foundation (HOGAN19G0, STANTO19R0), the National Science Foundation (1458359), the Gordon and Betty Moore Foundation (GBMF 4552), and the National Institutes of Health (T32 GM008704, R01 HG010067, R01 CA237170, and R01 CA200854).

## Author's Contributions

Alexandra Lee: Formal Analysis, Investigation, Methodology, Software, Visualization, Writing - Original Draft; Writing - Review and Editing; YoSon Park: Validation, Writing - Review and Editing; Georgia Doing: Methodology, Validation, Writing - Review and Editing; Deborah Hogan: Funding Acquisition, Methodology, Supervision, Validation, Writing - Review and Editing; Casey Greene: Conceptualization, Funding Acquisition, Methodology, Supervision, Validation, Writing - Original Draft, Writing - Review and Editing

## Supplementary Material

giaa117_GIGA-D-20-00127_Original_Submission

giaa117_GIGA-D-20-00127_Revision_1

giaa117_Response_to_Reviewer_Comments_Original_Submission

giaa117_Reviewer_1_Report_Original_Submission Hao Li -- 5/26/2020 Reviewed

giaa117_Reviewer_2_Report_Original_Submission Eric Lu Zhang -- 7/31/2020 Reviewed

giaa117_Reviewer_2_Report_Revision_1 Eric Lu Zhang -- 9/3/2020 Reviewed

giaa117_Supplemental_Figures

## References

[1] Perou CM . Show me the data! Nat Genet. 2001;29(4):373. 11726921 10.1038/ng1201-373

[2] Tan J, Hammond JH, Hogan DA, et al. ADAGE-based integration of publicly available *Pseudomonas aeruginosa* gene expression data with denoising autoencoders illuminates microbe-host interactions. mSystems. 2016;1(1), doi:10.1128/mSystems.00025-15. PMC5069748 27822512

[3] Tan J, Doing G, Lewis KA, et al. Unsupervised extraction of stable expression signatures from public compendia with an ensemble of neural networks. Cell Syst. 2017;5(1):63–71.e6. 28711280 10.1016/j.cels.2017.06.003 PMC5532071

[4] Chen L, Cai C, Chen V, et al. Learning a hierarchical representation of the yeast transcriptomic machinery using an autoencoder model. BMC Bioinformatics. 2016;17(Suppl 1):9. 26818848 10.1186/s12859-015-0852-1 PMC4895523

[5] Zhou W, Altman RB. Data-driven human transcriptomic modules determined by independent component analysis. BMC Bioinformatics. 2018;19(1):327. 30223787 10.1186/s12859-018-2338-4 PMC6142401

[6] Taroni JN, Grayson PC, Hu Q, et al. MultiPLIER: a transfer learning framework for transcriptomics reveals systemic features of rare disease. Cell Syst. 2019;8(5):380–94. 31121115 10.1016/j.cels.2019.04.003 PMC6538307

[7] Leek JT, Storey JD. Capturing heterogeneity in gene expression studies by surrogate variable analysis. PLoS Genet. 2007;3(9):1724–35. 17907809 10.1371/journal.pgen.0030161 PMC1994707

[8] Renard E, Absil PA. Comparison of batch effect removal methods in the presence of correlation between outcome and batch. PLos One. 2018;13(8):e0202947.30161168

[9] Tseng GC, Oh M-K, Rohlin L, et al. Issues in cDNA microarray analysis: quality filtering, channel normalization, models of variations and assessment of gene effects. Nucleic Acids Res. 2001;29(12):2549–57. 11410663 10.1093/nar/29.12.2549 PMC55725

[10] Kerr MK, Martin M, Churchill GA. Analysis of variance for gene expression microarray data. J Comput Biol. 2000;7(6):819–37. 11382364 10.1089/10665270050514954

[11] Chen C, Grennan K, Badner J, et al. Removing batch effects in analysis of expression microarray data: an evaluation of six batch adjustment methods. PLoS One. 2011;6(2):e17238. 21386892 10.1371/journal.pone.0017238 PMC3046121

[12] Johnson WE, Li C, Rabinovic A. Adjusting batch effects in microarray expression data using empirical Bayes methods. Biostatistics. 2007;8(1):118–27. 16632515 10.1093/biostatistics/kxj037

[13] Stegle O, Parts L, Piipari M, et al. Using probabilistic estimation of expression residuals (PEER) to obtain increased power and interpretability of gene expression analyses. Nat Protoc. 2012;7(3):500. 22343431 10.1038/nprot.2011.457 PMC3398141

[14] Taroni JN, Greene CS. Cross-platform normalization enables machine learning model training on microarray and RNA-Seq data simultaneously. bioRxiv. 2017:118349. 10.1038/s42003-023-04588-6 PMC9968332 36841852

[15] Parrish RS, Spencer HJ III, Xu P. Distribution modeling and simulation of gene expression data. Comput Stat Data Anal. 2009;53(5):1650–60.

[16] Singhal S, Kyvernitis CG, Johnson SW, et al. Microarray data simulator for improved selection of differentially expressed genes. Cancer Biol Ther. 2003;2(4):383–91. 14508110 10.4161/cbt.2.4.431

[17] Law CW, Chen Y, Shi W, et al. voom: Precision weights unlock linear model analysis tools for RNA-seq read counts. Genome Biol. 2014;15(2):R29. 24485249 10.1186/gb-2014-15-2-r29 PMC4053721

[18] Pimentel H, Bray NL, Puente S, et al. Differential analysis of RNA-seq incorporating quantification uncertainty. Nat Methods. 2017;14(7):687. 28581496 10.1038/nmeth.4324

[19] Greene CS., Hu D, Jones RWW, et al. refine.bio: A resource of uniformly processed publicly available gene expression datasets.

[20] Leinonen R, Sugawara H, Shumway M, et al. The Sequence Read Archive. Nucleic Acids Res. 2010;39(suppl_1):D19–21. 21062823 10.1093/nar/gkq1019 PMC3013647

[21] Edgar R, Domrachev M, Lash AE. Gene Expression Omnibus: NCBI gene expression and hybridization array data repository. Nucleic Acids Res. 2002;30(1):207–10. 11752295 10.1093/nar/30.1.207 PMC99122

[22] Brazma A, Parkinson H, Sarkans U, et al. ArrayExpress—A public repository for microarray gene expression data at the EBI. Nucleic Acids Res. 2003;31(1):68–71. 12519949 10.1093/nar/gkg091 PMC165538

[23] Kingma DP, Welling M. Auto-encoding variational bayes. arXiv. 2013:1312.6114.

[24] McInnes L, Healy J, Melville J. Umap: Uniform manifold approximation and projection for dimension reduction. arXiv. 2018:1802.03426.

[25] Ritchie ME, Phipson B, Wu D, et al. limma powers differential expression analyses for RNA-sequencing and microarray studies. Nucleic Acids Res. 2015;43(7):e47. 25605792 10.1093/nar/gkv007 PMC4402510

[26] Raghu M, Gilmer J, Yosinski J, et al. Svcca: Singular vector canonical correlation analysis for deep learning dynamics and interpretability. In: Guyon I, Luxburg UV, Bengio S et al. et al., eds. Advances in Neural Information Processing Systems. 2017:6076–85.

[27] Barbier M, Damron FH, Bielecki P, et al. From the environment to the host: Re-wiring of the transcriptome of *Pseudomonas aeruginosa* from 22°C to 37°C. PLoS One. 2014;9(2):e89941. 24587139 10.1371/journal.pone.0089941 PMC3933690

[28] Powers RK, Goodspeed A, Pielke-Lombardo H, et al. GSEA-InContext: Identifying novel and common patterns in expression experiments. Bioinformatics. 2018;34(13):i555–64. 29950010 10.1093/bioinformatics/bty271 PMC6022535

[29] Crow M, Lim N, Ballouz S, et al. Predictability of human differential gene expression. Proc Natl Acad Sci U S A. 2019;116(13):6491–500. 30846554 10.1073/pnas.1802973116 PMC6442595

[30] Tralau T, Vuilleumier S, Thibault C, et al. Transcriptomic analysis of the sulfate starvation response of*Pseudomonas aeruginosa*. J Bacteriol. 2007;189(19):6743–50. 17675390 10.1128/JB.00889-07 PMC2045191

[31] Lee AJ. simulate-expression-compendia repository (2019) [Source code]. https://github.com/greenelab/simulate-expression-compendia.

[32] Espín-Pérez A, Portier C, Chadeau-Hyam M, et al. Comparison of statistical methods and the use of quality control samples for batch effect correction in human transcriptome data. PLoS One. 2018;13(8):e0202947. 30161168 10.1371/journal.pone.0202947 PMC6117018

[33] Tan J, adage repository (2017) [Source code]. https://github.com/greenelab/adage/tree/master/Data_collection_processing.

[34] Collado-Torres L, Nellore A, Kammers K, et al. Reproducible RNA-seq analysis using recount2. Nat Biotechnol. 2017;35(4):319–21. 28398307 10.1038/nbt.3838 PMC6742427

[35] Weinstein JN, Collisson EA, Mills GB, et al. The Cancer Genome Atlas Pan-Cancer analysis project. Nat Genet. 2013;45(10):1113–20. 24071849 10.1038/ng.2764 PMC3919969

[36] The GTEx Consortium. The Genotype-Tissue Expression (GTEx) pilot analysis: Multitissue gene regulation in humans. Science. 2015;348(6235):648–60. 25954001 10.1126/science.1262110 PMC4547484

[37] Abadi M, Agarwal A, Barham P, et al. Tensorflow: Large-scale machine learning on heterogeneous distributed systems. arXiv. 2016:1603.04467.

[38] Chollet F . keras. (2015) [Source code]. https://github.com/fchollet.

[39] Way GP, Greene CS. Extracting a biologically relevant latent space from cancer transcriptomes with variational autoencoders. Pac Symp Biocomput. 2018;23:80–91. 29218871 PMC5728678

[40] Smyth Gordon K . Linear models and empirical bayes methods for assessing differential expression in microarray experiments. Stat Appl Genet Mol Biol. 2004;3(1):1–25. 10.2202/1544-6115.1027 16646809

[41] Yu G, Wang L-G, Han Y, et al. clusterProfiler: An R package for comparing biological themes among gene clusters. OMICS. 2012;16(5):284–7. 22455463 10.1089/omi.2011.0118 PMC3339379

[42] Lee AJ, ponyo repository (2019) [Source code]. https://github.com/greenelab/ponyo.

[43] Lee AJ, Park Y, Doing G, et al. Supporting data for “Correcting for experiment-specific variability in expression compendia can remove underlying signals.”. GigaScience Database. 2020. 10.5524/100796. PMC7607552 33140829

