# Supplementary material for: Correcting for experiment-specific variability in expression compendia can remove underlying signals
Source: Gigascience. 2020 Nov 3;9(11):giaa117. doi: 10.1093/gigascience/giaa117 (PMC7607552; doi:10.1093/gigascience/giaa117)
Supplement: giaa117_GIGA-D-20-00127_Revision_1 [file giaa117_giga-d-20-00127_revision_1.pdf]

GigaScience

Correcting for experiment-specific variability in expression compendia can remove underlying signals

--Manuscript Draft--

**Manuscript Number:**	GIGA-D-20-00127R1
**Full Title:**	Correcting for experiment-specific variability in expression compendia can remove underlying signals
**Article Type:**	Research
**Funding Information:**	Cystic Fibrosis Foundation (HOGAN19G0) — Prof Deborah A Hogan<br>Cystic Fibrosis Foundation (STANTO19R0) — Prof Deborah A Hogan<br>National Science Foundation (1458359) — Ms Georgia Doing, Prof Casey S Greene, Prof Deborah A Hogan<br>National Institute of General Medical Sciences (5T32GM008704) — Ms Georgia Doing<br>Gordon and Betty Moore Foundation (GBMF 4552) — Prof Casey S Greene<br>National Human Genome Research Institute (R01 HG010067) — Prof Casey S Greene<br>National Cancer Institute (R01 CA237170) — Prof Casey S Greene
**Abstract:**	Motivation: In the last two decades, scientists working in different labs have assayed gene expression from millions of samples. These experiments can be combined into compendia and analyzed collectively to extract novel biological patterns. Technical variability, sometimes referred to as batch effects, may result from combining samples collected and processed at different times and in different settings. Such variability may distort our ability to interpret and extract true underlying biological patterns. As more integrative analysis methods are developed and available data collections are increased in size, it is crucial to determine how technical variability affect our ability to detect desired patterns when many experiments are combined<br>Objective: We sought to determine the extent to which an underlying signal was masked by technical variability by simulating compendia comprised of data aggregated across multiple experiments.<br>Method: We developed a generative multi-layer neural network to simulate compendia of gene expression experiments from large-scale microbial and human datasets. We compared simulated compendia before and after introducing varying numbers of sources of undesired variability.<br>Results: We found that the signal from a baseline compendium was obscured when the number of added sources of variability was small. Perhaps as expected, applying statistical correction methods rescued the underlying signal in these cases. However, as the number of sources of variability increased we observed that detecting the original signal became increasingly easier even without correction. In fact, applying statistical correction methods reduced our power to detect the underlying signal.<br>Conclusion: When combining a modest number of experiments, it is best to correct for experiment-specific noise. However, when many experiments are combined, statistical correction reduces our ability to extract underlying patterns.
**Corresponding Author:**	Casey S. Greene<br><br>UNITED STATES
**Corresponding Author Secondary Information:**	
**Corresponding Author's Institution:**	

Powered by Editorial Manager® and ProduXion Manager® from Aries Systems Corporation

**Corresponding Author's Secondary Institution:**	
**First Author:**	Alexandra J Lee
**First Author Secondary Information:**	
**Order of Authors:**	Alexandra J Lee
	YoSon Park
	Georgia Doing
	Deborah A Hogan
	Casey S Greene
**Order of Authors Secondary Information:**	
**Response to Reviewers:**	We uploaded the response to reviewer as a cover letter because we embedded figures to provide clarity and a self-contained document.
**Additional Information:**	
**Question**	**Response**
Are you submitting this manuscript to a special series or article collection?	No
**Experimental design and statistics**<br><br>Full details of the experimental design and statistical methods used should be given in the Methods section, as detailed in our Minimum Standards Reporting Checklist. Information essential to interpreting the data presented should be made available in the figure legends.<br><br>Have you included all the information requested in your manuscript?	Yes
**Resources**<br><br>A description of all resources used, including antibodies, cell lines, animals and software tools, with enough information to allow them to be uniquely identified, should be included in the Methods section. Authors are strongly encouraged to cite Research Resource Identifiers (RRIDs) for antibodies, model organisms and tools, where possible.<br><br>Have you included the information requested as detailed in our Minimum Standards Reporting Checklist?	Yes

Powered by Editorial Manager® and ProduXion Manager® from Aries Systems Corporation

**Availability of data and materials**<br><br>All datasets and code on which the conclusions of the paper rely must be either included in your submission or deposited in publicly available repositories (where available and ethically appropriate), referencing such data using a unique identifier in the references and in the "Availability of Data and Materials" section of your manuscript.<br><br>Have you have met the above requirement as detailed in our Minimum Standards Reporting Checklist?	Yes

Manuscript

Click here to
access/download;Manuscript;compendium_manuscript_revised_

Correcting for experiment-specific variability in expression compendia can remove underlying signals

Alexandra J. Lee[1,2], YoSon Park[2], Georgia Doing[3], Deborah A. Hogan[3], Casey S. Greene[2,4]

[1] Genomics and Computational Biology Graduate Program, University of Pennsylvania, Philadelphia, PA, USA
[2] Department of Systems Pharmacology and Translational Therapeutics, University of Pennsylvania, Philadelphia, PA, USA
[3] Department of Microbiology and Immunology, Geisel School of Medicine, Dartmouth, Hanover, NH, USA
[4] Childhood Cancer Data Lab, Alex's Lemonade Stand Foundation, Philadelphia, PA, USA

ORCID IDs:

Alexandra Lee: 0000-0002-0208-3730; Georgia Doing: 0000-0002-0835-6955; Deborah Hogan: 0000-0002-6366-297; Casey Greene: 0000-0001-8713-9213

Email: greenescientist@gmail.com

Abstract:

Motivation: In the last two decades, scientists working in different labs have assayed gene expression from millions of samples. These experiments can be combined into compendia and analyzed collectively to extract novel biological patterns. Technical variability, sometimes referred to as batch effects, may result from combining samples collected and processed at different times and in different settings. Such variability may distort our ability to interpret and extract true underlying biological patterns. As more integrative analysis methods are developed and available data collections are increased in size, it is crucial to determine how technical variability affect our ability to detect desired patterns when many experiments are combined

Objective: We sought to determine the extent to which an underlying signal was masked by technical variability by simulating compendia comprised of data aggregated across multiple experiments.

Method: We developed a generative multi-layer neural network to simulate compendia of gene expression experiments from large-scale microbial and human datasets. We compared simulated compendia before and after introducing varying numbers of sources of undesired variability.

Results: We found that the signal from a baseline compendium was obscured when the number of added sources of variability was small. Perhaps as expected, applying statistical correction methods rescued the underlying signal in these cases. However, as the number of sources of variability increased we observed that detecting the original signal became increasingly easier even without correction. In fact, applying statistical correction methods reduced our power to detect the underlying signal.

Conclusion: When combining a modest number of experiments, it is best to correct for experiment-specific noise. However, when many experiments are combined, statistical correction reduces our ability to extract underlying patterns.

Introduction:

Over the last two decades, unprecedented amounts of transcriptome-wide gene expression profiling data have been generated. Most of these datasets are shared in public platforms for the research community.[1] Researchers are now combining samples across different experiments to form compendia, and analyzing these compendia is revealing new biology.[2–6] It is well-understood that technical sources of variability pervade large-scale data analysis such as transcriptome-wide expression profiling studies.[7–10] Numerous methods have been designed to correct for various types of effects.[7,11–13] Despite the prevalence of technical sources of variability, researchers have successfully extracted biological patterns from multi-experiment compendia without applying correction methods.[2–5,14] To determine the basis of these seemingly contradictory results, we examined the extent to which underlying statistical structure can be extracted from compendium-style datasets in the presence of sources of undesired variability.

A number of methods have been developed to simulate transcriptome-wide expression experiments.[15–18] However, these existing approaches require defining a statistical model that describes the process by which researchers design and carry out experiments, which is often very challenging to obtain. Instead, we developed an approach to simulate compendia by sampling from the low-dimensional representation produced by multi-layer generative neural networks trained on gene expression data from an existing compendium. This allowed us to simulate gene expression experiments that mimic real experimental configurations. We combined these experiments to create compendia.

Using this simulation approach, we studied how adding varying amounts of experiment-specific noise affects our ability to detect underlying patterns in the gene expression compendia. This topic is becoming pressing as more large-scale expression compendia are becoming available. We found that prior reports of pervasive technical noise and analyses that succeed without correcting for it are, in fact, consistent. In settings with relatively few experiment-specific sources of undesired variation, the added noise substantially alters the structure of the data. In these settings, statistical correction produces a data representation that better captures the original variability in the data. On the other hand, when the number of experiment-specific sources of undesired variability is large, attempting to correct for these sources does more harm than good.

Results:

We characterized publicly available data compendia using refine.bio[19], a meta-repository that integrates data from multiple different repositories. We found that, on average, experiments contained hundreds to thousands of samples in most widely studied organisms (Table 1). These samples were derived from hundreds to thousands of experiments, and the most common experimental designs had relatively few samples (medians from 5-12). We compared compendia from refine.bio to two readily available compendia, recount2 and one for *P. aeruginosa*, that have been used for compendium-wide analyses.[2,3,6] The compendia that have been successfully used in prior work[2,3,6] have similar median numbers of samples per experiment (recount2 = 4, *P. aeruginosa* = 6) to the current publicly available data.

Table 1: *Public data usually have only a modest number of samples per experiment, though many samples are available in aggregate. Statistics for the 10 largest transcriptomic compendia found in refine.bio, which is a meta-repository containing publicly available expression data from the Sequence Read Archive (SRA)[20], Gene Expression Omnibus (GEO)[21] and ArrayExpress[22].*

	**No. experiments**	**Median no. samples**	**Total no. samples**
Homo sapiens	15,440	12	571,862
Mus musculus	13,224	10	296,829
Arabidopsis thaliana	1,627	9	24,855
Rattus norvegicus	1,368	12	38,530
Drosophila melanogaster	853	9	17,836
Saccharomyces cerevisiae	627	12	12,972
Danio rerio	546	9.5	28,518
Caenorhabditis elegans	375	10	7,953
Sus scrofa	280	12	6,063
Zea mays	274	5	3,458

Constructing a generative model for gene expression samples

We developed an approach to simulate new gene expression compendia using generative multi-layer neural networks. Specifically, we trained a variational autoencoder (VAE)[23], which was comprised of an encoder and decoder neural network. The encoder neural network compressed the input data through two layers into a low-dimensional representation and the decoder neural network expanded the dimensionality back to the original input size. The VAE learned a low-dimensional representation that can reconstruct the original input data.

Simultaneously, the VAE optimized the lowest dimensional representation to follow a normal distribution (Figure 1A). This normal distribution constraint, which distinguishes VAE's from other types of autoencoders, allowed us to generate variations of the input data by sampling from a continuous latent space.[23]

We trained VAEs for each compendium: recount2 (896 samples with 58,037 genes) and *P. aeruginosa* (989 samples with 5,549 genes). We evaluated the training and validation set losses at each epoch, which stabilized after roughly 100 epochs (Figure 1B). We observed a similar stabilization after 40 epochs for recount2 (Figure S1A). We simulated new genome-wide gene expression data by sampling from the latent space of the VAE using a normal distribution (Figure 1C). We used UMAP[24] to visualize the structure of the original and simulated data and found that the simulated data generally fell near original data for both compendia (Figure 1D; Figure S1B).

Simulating gene expression compendia with synthetic samples

We designed a simulation study to assess the extent to which artifactual noise associated with individual partitions of a large compendium affects the structure of the overall compendium. Our simulation is akin to asking: if different labs performing transcriptome-wide experiments randomly sampled from the available set of possible conditions, to what extent would experiment-specific biases dominate the signal of the data. First, we simulated new compendia. Then we randomly divided the samples within these compendia into partitions and added noise to each partition. Finally, we compared the simulated compendia with added noise to the unpartitioned one (Figure 2A). Each partition represented groups of samples with shared experiment-specific noise. We evaluated the similarity before and after applying an algorithm designed to correct for technical noise in each partition – given that the added noise was linear, we used limma[25] to correct. Singular Vector Canonical Correlation Analysis (SVCCA)[26] was used to assess similarity. The SVCCA analysis measured the correlation between the distribution of gene expression in the compendia without noise compared to the distribution in the compendia with multiple sources of technical variance.

We performed a study with this design using the VAE trained from the *P. aeruginosa* compendium. We simulated a *P. aeruginosa* compendium with 6,000 samples for [1, 2, 5, 10, 20, 50, 100, 500, 1000, 2000, 3000, 6000] partitions. We found that adding technical variance to

partitions always reduced the similarity between the simulated data without partitions and the partitioned simulated data. However, the nature of the change in similarity differed substantially between the partitioned compendia before and after the correction step (Figure 2B). With the correction step (dark blue line) similarity dropped throughout the range of the study, eventually reaching the same level as the permuted data (dashed grey line). Without the correction step (light blue line), similarity dropped immediately to the random level and then recovered throughout the rest of the tested range. We visualized the simulated data on the top 2 principle components from the original data (Figure 2C, grey points). The corrected (Figure 2C, dark blue) and uncorrected (Figure 2C, light blue) data at various numbers of partitions revealed that the correction step removes both wanted and unwanted variability, eventually removing all variability in the data. Without correction, the data were initially dramatically transformed. However, as the number of partitions grows very large the effect on the structure of the data was diminished.

To determine whether or not this correction removing signal was a more general property of such compendia, we repeated the same simulation study using a VAE trained on a recount2 compendium. recount2 is a compendium comprised of human RNA-seq samples, so it is generated using a different technology and consists of assays of a very different organism. We simulated a compendium with 500 samples for [1, 2, 5, 10, 20, 50, 100, 250, 500] partitions. The results with recount2 mirrored our findings with the *P. aeruginosa* compendium. The correction step initially retained more similarity, but performance crossed over and by 500 partitions the uncorrected data were more similar to the unpartitioned simulated compendium (Figure 2D). Visualizing the top principle components, again, revealed that correction restored the structure of the original data with few partitions, but with many partitions the structure was better retained without correction (Figure 2E). Additionally, the same trends were observed when we varied the magnitude of the noise added (Figure S2) or used a different noise correction method, such as COMBAT[12] (Figure S3). In general, there exists some minimum number of experiment-specific sources of noise that determines the effectiveness of applying noise correction to these multi-experiment compendia.

A generative model for gene expression experiments

We randomly selected samples from the range of all possible samples in the compendium. This next simulation added another level of complexity to the model, by simulating experiments as

opposed to samples to make the simulated compendia more representative of true expression data. This simulation generated synthetic experiments for which the gene expression patterns were consistent with those from the types of experiments that are used within the field. The technique that we developed uses the same underlying approach of sampling from a VAE. However, in this case we randomly selected a template experiment (E-GEOD-51409, which compared *P. aeruginosa* at 22˚C and 37˚C) and a vector that would move that template experiment to a new location in the gene expression space (Figure 3A). The simulation preserved the relationship between samples within the template experiment while also shifting the activity of the samples in the latent space (Figure 3B). Intuitively, this process maintained the relationship between samples but changed the underlying perturbation; this simulation maintained the same experimental design but is akin to studying a distinct biological process. We used this process to generate compendia of new gene expression experiments. We then examined the retention of the original differential expression signature by comparing the set of differentially expressed genes (DEGs) found in the simulated experiments (Figure 3D). Applying only the VAE compression to the original experiment (E-GEOD-51409), generated an experiment that had the same sample grouping as the original. However, only a subset of the DEGs found in the VAE compressed experiment were also found in the original experiment. The VAE compression step added some noise to the expression signal in the original experiment, as expected, since the data was being compressed into a low dimensional space. Overall, the correlation between the genes, based on their log2 fold-change values, in the original and VAE compressed experiment was high, $R^2 = 0.822$ (Figure 3C). Next, we exampled how the original samples in an experiment and a simulated experiment, applying VAE compression and latent space translation of the E-GEOD-51409 experiment, had consistent clustering of samples (Figure 3D original and experiment-level simulated experiment).[27] However the sets of genes that were differentially expressed were different between the two experiments. This demonstrated that the perturbation intensity and experimental design were relatively consistent in gene expression space, even though the nature of the perturbation differed. The correlation between genes in the original and the experiment-level experiment was lower, $R^2 = 0.230$, since it represented a unique experiment. The residual similarity was likely due to commonly differentially expressed genes that have been observed previously[28,29]. Finally, as a control, we demonstrated that the original experiment structure was not well preserved using the random sampling approach (Figure 3D, sample-level simulated experiment). The correlation between genes in the original and sample-level experiment was non-existent, $R^2 = -0.055$, since we did not account for experiment structure in the sample-level simulation.

In general, the numbers of differentially expressed genes found in the experiment-preserving simulated experiments (78 DEGs in VAE compressed, 14 DEGs in experiment-level) were lower compared to the original experiment (505 DEGs). This was because the simulated experiments had a lower variance compared to the original experiment. This reduced variance was due to the normality assumption made by the VAE, which compressed the latent space data representation.[23] However, the clustering of samples was conserved between the simulated and original experiments and this was also observed in the additional template experiments with more complex experimental setups (Figure S4). Given the fact that we preserved the association between samples and experiments in this new experiment-level simulation, we expected that simulated experiments would preserve the correlation in expression of genes that are in the same pathway. In our previous example, the simulated experiment generated using the original E-GEOD-51409 as a template (i.e. experiment-level, Figure 3A) identified 14 DEGs (Figure 3D). In contrast, the simulated experiment generated by random sampling (i.e. sample-level, Figure 1C) did not identify any DEGs; the median log2 fold-change was 0.08. Furthermore, simulating 100 new experiments using E-GEOD-51409 as a template, identified a median of 2,588 DEGs compared to simulated experiments generated by random sampling which identified a median of 0 DEGs (Figure 3E). Additionally, the median number of enriched KEGG pathways was 1 using the template shifting approach compared to 0 using the random sampling approach (Figure 3F). Overall, it appeared that this new simulation approach generated a compendium of more realistic experiments with underlying biology. (examples of the significantly enriched pathways in Table 2). The top over-represented pathway was the ribosome pathway, which is likely a commonly altered pathway found in many experiments regardless of experiment type, similar to the findings from human array experiments in Crow et. al.[28,29] The remaining pathways found in the original experiment were related to metabolism, which is consistent with the finding from the original publication.[27] The simulated experiment was particularly enriched in sulfur metabolism and ABC transporters, which is consistent with an experiment that found upregulation of transport systems in response to sulfate limitations.[30] Overall, in accordance with real gene expression experiments, the new simulated experiments contain related groups of enriched pathways that reflect the specific hypotheses being tested. These results demonstrate the use of a VAE as a hypothesis generating tool. We can now simulate new experiments in order to study the response of *P. aeruginosa* in response to untested conditions.

Table 2: *Enriched pathways found in the original E-GEOD-51409 experiment and the pseudo-experiment generated using the experiment-level simulation.*

Original	Adjusted p-value	Experiment level simulation	Adjusted p-value
Pae03010: Ribosome	2.966E-11	Pae03010: Ribosome	7.96E-07
Pae00500: Starch and sucrose metabolism	1.512E-03	Pae02010: ABC transporters	4.009E-03
Pae01200: Carbon metabolism	4.466E-03	Pae00920: Sulfur metabolism	1.576E-02
Pae00640: Propanoate metabolism	1.954E-03		

Simulating gene expression compendia with synthetic experiments

We used our method to simulate new experiments that followed existing patterns to examine the patterns from generic partitions (Figure 4A). We simulated 600 experiments using the *P. aeruginosa* compendium. We divided these experiments into [1, 2, 3, 5, 10, 20, 30, 50, 70, 100, 200, 300, 400, 500, 600] partitions. These partitions represented groupings of experiments with shared noise, such as experiments from the same lab or experiments with the same experimental design. Each partition contained technical sources of variance within and between experiments. Results with simulated experiments were similar to those from arbitrarily partitioned samples. We observed a monotonic loss of similarity after the correction step as the number of partitions increased (Figure 4B). Visualizing the top principal components revealed that statistical correction initially better recapitulated the overall structure of the data but that similarity decreased with many partitions (Figure 4C, dark blue). Without statistical correction there was a larger initial drop in similarity but a later recovery (Figure 4B) and visualizing the top principal components recapitulated this finding (Figure 4C, light blue). We performed analogous experiments using the recount2 VAE and 50 simulated experiments with [1, 2, 5, 10, 20, 30, 50] partitions. We observed consistent results with this dataset using both SVCCA similarity (Figure 4D) and visual inspection of the top principal components (Figure 4E).

One caveat in the design of the previous analysis, is that the effect of the number of partitions was confounded by the number of experiments per partition. For example, more partitions equated to each partition having a smaller effect size since each partition had fewer experiments. To study the contribution of individual experiments in our signal detection, we

performed an analysis where we held the number of experiments per partition fixed and varied the number of total experiments within a compendia (Figure S5A). With few experiments in a compendia, the main signal was the difference between experiments so adding noise to each experiment drove signal detection down (Figure S5B). Additionally, applying noise correction removed the main experiment-specific signal, as it was designed to do. With more experiments in a compendia, we gained a more global gene expression representation, where the main signal was no longer focused on the difference between experiments. Thus, adding noise to each experiment did not affect our signal detection and our similarity remained constant. However, applying noise correction will consistently remove more of our signal of interest. The results of this analysis exemplify how existing experiments can be combined and used without need for correction.

In summary, as the number of partitions or experiments increase the experiment-specific technical sources contribute less to the overall signal and the underlying patterns dominate the overall signal. When many partitions or experiments are present, even ideal statistical approaches to correct for noise over-corrects and removes the underlying signal.

Discussion:

Our findings reveal that compendia-wide analyses do not always require correction for experiment-specific technical variance and that correcting for such variance may remove signal. This simulation study provides an explanation for the observation that past studies[2–6] have successfully extracted biological signatures from gene expression compendia despite the presence of uncorrected experiment-specific sources of technical variability. In general, there exists compendia that contain some small number of experiment-specific sources where traditional correction methods can be effective at recovering the biological structure of interest. However, there also exist large-scale gene expression compendia where these methods may be harmful instead of helpful. The number of experiment-specific sources that determine whether to apply correction will vary depending on the size of the compendia and the magnitude and structure of the signals. Using the associated repository (https://github.com/greenelab/simulate-expression-compendia) users can customize the scripts to run the simulation experiments on their own expression data in order to examine the effect of a linear noise model with linear noise correction on their dataset. Though our analysis uses simplifying assumptions that preclude us from defining a specific threshold for noise correction,

these simulations define a set of general properties that will guide compendia analyses moving forward. This study suggests that new large-scale datasets can be created by distributing different experiments across many different labs and centers as opposed to being consolidated within a single lab.

We introduce a new method to simulate genome-wide gene expression experiments, using existing gene expression data as starting material, which goes beyond simulating individual samples. This allows us to examine the extent to which our findings hold with realistic experimental designs. The ability to simulate gene expression experiments with a realistic structure has many potential legitimate uses: pre-training for machine learning models, providing synthetic test data for software, and other such applications. Additionally, this simulation technique can be used to explore hypothetical experiments that have not been previously performed and generate hypotheses. However, such approaches could also be used by nefarious actors to generate synthetic data for publications. Forensic tools that detect synthetic genome-wide data may be needed to combat potential fraudulent uses.

Our study has several limitations. We assume a certain noise model that differs between experiments. However, the sources of real noise are multifaceted and any such assumption will necessarily be an oversimplification, though such assumptions are not uncommon.[10,12,31] By selecting a specific noise model and using an ideal noise-removal step, we provide a best case scenario for artifact removal. While any simulation study will necessarily make simplifying assumptions, this work is the first to use deep generative models as part of a simulation study to probe the long-standing assumption that correcting for technical variability is necessary for analyses that span multiple experiments. Our findings reveal that in settings with hundreds or thousands of experiments, correcting for experiment-specific effects can harm performance and that it can be best to forgo statistical correction. Adjusting the choices of normalization, noise magnitude, and noise patterns will result in different selections of the precise cross-over point where it becomes beneficial to perform correction. With this design, we do not expect to estimate exactly where this precise cross-over point is. Such an estimation would require a compendium where investigators systematically performed the same combination of different experiments in multiple labs at different times. We were unable to identify such a compendium on the scale of thousands of samples from tens to hundreds of labs. Thus, though our analysis necessarily includes simplifying assumptions that limit our ability to precisely define the

thresholds for correction for arbitrary datasets and noise sources, it remains suitable for examining the overriding principles that govern compendium-wide analyses.

Our study has broad implications for efforts to standardize scientific processes. Centralization of large-scale data generation has the potential to reduce experiment-specific technical noise, though it comes at a cost of flexibility. Our results suggest that a highly distributed process where experiments are carried out in many different locations, with their own specific sources of technical noise, can also lead to valuable data collections.

Methods:

Pseudomonas aeruginosa gene expression compendium

We downloaded a compendium of *P. aeruginosa* data that was previously used for compendium-wide analyses.[2] Previous studies identified biologically-relevant processes such as oxygen deprivation[2] and phosphate starvation[3] by applying denoising autoencoders. We obtained the processed and normalized gene expression matrices from the ADAGE GitHub repository (https://github.com/greenelab/adage/tree/master/Data_collection_processing). The *P. aeruginosa* dataset was previously processed by Tan et. al. During processing, raw microarray data were downloaded as .cel files, *rma* was used to convert probe intensity values from the *.cel* files to log2 base gene expression measurements, and these gene expression values were then normalized to 0-1 range per genes.

This compendium includes measurements from 107 experiments that contain 989 samples for 5,549 genes.[2] It contains experiments that accrued between the release of the GeneChip *P. aeruginosa* genome array and the time of data freeze in 2014. Approximately 70% of the samples were from cultures of strain PAO1 and derivatives, 13% were in strain PA14 background, 0.6% were from PAK strains and the remaining were largely clinical isolates. Of the strains, 73% were wild-type (WT) genotypes and the rest were mutants that had undergone genetic modification. Approximately 60% of the samples were grown in lysogeny broth (LB) medium while the rest were grown in Pseudomonas Isolation Agar (PIA), glucose, pyruvate or amino acid-based media.[3] Roughly 80% were grown planktonically, 15% were grown in biofilms and the remaining samples were in vivo or not annotated. Overall, this *P. aeruginosa* compendium covered a wide range of gene expression patterns including: characterization of

clinical isolates from cystic fibrosis infections, differences between mutant versus WT, response to antibiotic treatment, microbial interactions, adaptation from water to GI tract infection. Despite having 989 samples, this compendium represents the heterogeneity of *P. aeruginosa* gene expression.

recount2 gene expression compendium

We downloaded human RNA-seq data from recount2.[32] The dataset includes over 70,000 samples collected from Sequencing Read Archive (SRA). It is comprised of more than 50,000 samples from different types of experiments, roughly 10,000 samples from Genotype-Tissue Expression project (GTEx v6) covering 44 types of normal tissue, and more than 10,000 samples from The Cancer Genome Atlas (TCGA) measuring 33 cancer types.[20,33,34] The recount2 authors uniformly processed and quantified these data. We downloaded data using the recount library in Bioconductor (version 1.14.0).[32] The entire recount2 dataset is 8TB. Based on the *P. aeruginosa* compendium we expected that a subset of the compendium would be sufficient for this simulation, so we selected a random subset of 50 NCBI studies, which resulted in 896 samples with 58,037 genes for our simulation. Each project (imported from NCBI bioproject) is akin to an experiment in the *P. aeruginosa* compendium, and we used the term *experiment* to describe different projects in order to maintain consistency in this paper. The downloaded recount2 dataset was in the form of raw read counts, which was normalized to produce RPKMs used in our analysis. The normalized gene expression data was then scaled to a 0-1 range per gene.

Strategy to construct VAE: structure and hyperparameters

We designed an approach to simulate gene expression compendia with a multi-layer variational autoencoder (VAE). We built this model in Keras (version 2.1.6) with a TensorFlow backend (version 1.10.0), modifying the previously published Tybalt method.[35–37] Our architecture used each input gene as a feature. These genes were compressed to 2,500 intermediate features using a rectified linear unit (ReLU) activation function to combine weighted nodes from the previous layer. These features were encoded into 30 latent space features, also using a ReLU activation function, which were optimized via the addition of a Kullbach-Leibler (KL) divergence term into the loss function (binary cross entropy) to follow a standard normal distribution. These features were then reconstructed back to the input feature dimensions using decoding layers that mirror the structure of the encoder network. We trained the VAE using 90% of the input

dataset, leaving 10% as a validation set. We determined training hyperparameters by manually adjusting parameters and selecting the parameters that optimized the validation loss based on visual inspection. These were a learning rate of 0.001, a batch size of 100, warmups set to 0.01, 100 epochs for the *P. aeruginosa* compendium and 20 epochs for the recount2 compendium. A similar assessment was performed to determine the neural network architecture. We manually inspected the validation loss using multiple different 2-layer designs (300-10, 2500-10, 2500-20, 2500-30, 2500-100, 2500-300) and found a 2,500 layer to a 30 hidden layer VAE to be most optimal.

Sample-based simulation

We used the VAE trained from each compendium to generate new compendia by randomly sampling from the latent space. We generated a simulated compendium containing 6,000 *P. aeruginosa* samples or 500 recount2 samples. For our first simulation, we sampled randomly - ignoring the relationship between samples within a specific experiment. We simulated experiment-specific sources of undesired variability within compendia by dividing the data into partitions and adding noise to each partition.

We divided the *P. aeruginosa* simulated compendium into [1, 2, 5, 10, 20, 50, 100, 500, 1000, 2000, 3000, 6000] partitions and divided the recount2 simulated compendium into [1, 2, 5, 10, 20, 50, 100, 250, 500] partitions. Each partition of data represented a group of samples from the same experiment or lab. We randomly added linear noise to each partition by generating a vector of length equal to the number of genes (5,549 *P. aeruginosa* genes and 58,037 human genes) where each value in the vector was drawn from a normal distribution with a mean of 0 and a variance of 0.2. With the 0-1 scaling, a value of 0.2 produces a relatively large difference in gene expression space (Figure S1). Though linear noise is an over-simplification of the types of noise that affect gene expression data, it allowed us to design an approach to optimally remove noise.

Experiment-based simulation

For the experiment-level simulation, we developed an approach that could simulate realistic experimental structure. There was no consistent set of annotated experimental designs, so we developed a simulation method that did not depend on *a priori* knowledge of experimental

design. For each synthetic experiment, we randomly sampled a “template experiment” from the set of *P. aeruginosa* or recount2 experiments*.* We then simulated new data that matched the template experiment by selecting a random location from the low dimensional representation of the simulated compendia (i.e. selecting a location according to the low dimensional distribution) and calculating the vector that connected this random location and the encoded template experiment. We then linearly shifted the template experiment in the low-dimensional latent space by adding this vector to each sample in the experiment. This process preserved the relationship between samples within the experiment but shifted the samples to a new location in the latent space. Repeating this process for each experiment allowed us to generate new simulated compendia comprised of realistic experimental designs.

We divided the *P. aeruginosa* simulated compendium into [1, 2, 3, 5, 10, 20, 30, 50, 70, 100, 200, 300, 400, 500, 600] partitions and divided the recount2 simulated compendium into [1, 2, 5, 10, 20, 30, 50] partitions, where experiments are divided equally amongst the partitions. For each partition we added simulated noise as described in the previous section. Experiments within the same partition had the same noise added. Each partition represented a group of experiments generated from the same lab or with the same experimental design.

Experiment-effect analysis

For this analysis we wanted to examine the effect of individual experiments in our ability to detect underlying gene expression structure. First, we used the experiment-based simulation approached to simulate *P. aeruginosa* compendia with [2, 3, 5, 10, 20, 30, 50, 70, 100, 200, 300, 400, 500, 600] experiments. Next, we divided the simulated compendium into the same number of partitions so that there was one experiment per partition. For each partition we added simulated noise as described in the previous section. Finally we used SVCCA to compare the noisy compendia with X number experiments with the unpartitioned compendia with X number of experiments. We also used SVCCA to compare the noise-corrected compendia with X experiments with the unpartitioned compendia with X experiments.

Removing technical variability from noisy compendia

Our model of undesired variability was a linear signature applied separately to each partition of the data, which we considered akin to experiments or groups of experiments in a compendium

of gene expression data. We used the removeBatchEffect function in the R library, limma (RRID:SCR_010943, version 3.44.0), to correct for the technical variation that was artificially added to the simulated compendia.[25] Limma removes the technical noise by first fitting a linear model to describe the relationship between the input gene expression data and the experiment labels. The input expression data contains both a biological signal and technical noise component. By fitting a linear model, limma will extract the noise contribution and then subtract this from the total input expression data. This method presents a best-case scenario for removing the undesired variability in the simulated compendia because the model matches the noise pattern we used in the simulation.

Measuring the similarity of matched compendia

We used Singular Vector Canonical Correlation Analysis (SVCCA)[26] to estimate similarities between different compendia. SVCCA is a method designed to compare two data representations[26]. Given two multivariate datasets, X_1 and X_2, the goal of SVCCA is to find the basis vectors, *w* and *s*, to maximize the correlation between w^TX^1 and s^TX_2. In other words, SVCCA attempts to find the space, defined by a set of basis vectors, such that the projection of the data onto that space is most correlated. Two datasets are considered similar if their linearly invariant correlation is high (i.e., if X_1 is a shift or rotation of X_2 then X_1 and X_2 are considered similar).

We compared the statistical structure of the gene expression, projected onto the first 10 principle components, in the baseline simulated compendia (those with only one experiment or partition, X_1) versus those with multiple experiments or partitions (X_2). Our SVCCA analysis was designed to measure the extent to which the gene expression structure of the compendia without noise was similar to the gene expression structure of the compendia with multiple sources of technical variance added as well as those where correction has been applied. Here we use 10 principle components for computational simplicity. Selecting a different value would affect the crossover point but not the general trends that we describe

A case study of differential expression in a template experiment

We compared the E-GEOD-51409 experiment[38] with two different simulated representations to provide a case study for experiment-based simulation. E-GEOD-51409 included *P. aeruginosa*

in two different growth conditions. For one simulation, we generated random samples and randomly assigned them to conditions, which we termed the sample-simulated experiment. For the second we used the latent space transformation process described above, which we termed the experiment-simulated experiment. We used the eBayes module in the limma library to calculate differential gene expression values for each gene between the two different growth conditions in the real and simulated data. We built heatmaps for the 14 most differentially expressed genes, where differentially expressed genes where those with FDR adjusted cutoff (using Benjamini-Hochberg correction) < 0.05 and log2 fold-change >1, which are thresholds frequently used in practice. We selected 14 genes because there were 505, 14 and 0 differentially expressed genes found in the original experiment, experiment-simulated experiment and sample-simulated experiment, respectively. Since there were 0 differentially expressed genes found in the sample-simulated experiment, we displayed the top 14 genes sorted by adjusted p-value to provide a visual summary of the simulation process.

Comparing sample-level and experiment-level simulated datasets

We simulated 100 experiments using the template E-GEOD-51409 experiment[38]. We sought to compare the sample-level and experiment-level simulation processes. We set a threshold for differentially expressed genes at a Bonferroni-corrected p-value cutoff of 0.05/5549. We used the enrichKEGG module in the clusterProfiler library (clusterProfiler, RRID:SCR_016884) to conduct an over-representation analysis[39]. We used the Fisher's exact test to calculate a p-value for over-representation of pathways in the set of differentially expressed genes. We considered pathways to be over-represented if the q-value was less than 0.02.

Implementation and Software Availability

All scripts to reproduce this analysis are available the GitHub repository (https://github.com/greenelab/simulate-expression-compendia) under an open source license. The repository contains 98% python jupyter notebooks, 2% python and 0.1% R scripts. The repository's structure is separated by input dataset. Pseudomonas/ and Human/ directories each contain the input data in the data/input/ directory. Scripts for the sample level simulation can be found in Pseudomonas /Pseudomonas_sample_lvl_sim.ipynb for the *P. aeruginosa* compendium and Human/Human_sample_lvl_sim.ipynb for the recount2 compendium. Scripts for the experiment level simulation can be found in

Pseudomonas/Pseudomonas_experiment_lvl_sim.ipynb and Human/Human_experiment_lvl_sim.ipynb respectively. The virtual environment was managed using conda (version 4.6.12), and the required libraries and packages are defined in the environment.yml file. Additionally, scripts to simulate gene expression compendia using the sample-level and experiment-level approaches are available as a separate module, called *ponyo*, and can be installed from PyPi (https://github.com/greenelab/ponyo). We describe in the Readme file how users can analyze different compendia or use different noise patterns. All simulations were run on a CPU.

Availability of supporting source code and requirements

- Project name: Simulate Expression Compendia
- Project home page: https://github.com/greenelab/simulate-expression-compendia
- Operating systems: Mac OS, Linux
- Programming language: Python, R
- Other requirements: Git LFS
- License: BSD v3

Availability of supporting data

An archival copy of the GitHub repository (including scripts and result files) is available in the *GigaScience* GigaDB repository[40].

Figure Legends:

Figure 1. Simulating gene expression data using VAE. A) Architecture of the VAE, where the input data gets compressed into intermediate layer of 2500 features and then into a hidden layer of 30 latent features. Each latent feature follows a normal distribution with mean μ and variance σ. The input dimensions of the *P. aeruginosa* dataset are shown here as an example (989 samples, 5549 genes). The same architecture is used to train the recount2 dataset except the input has 896 samples and 58,037 genes. B) Validation loss plotted per epoch during training using the P. aeruginosa compendium. C) Workflow to simulate gene expression samples from a compendium model, where new samples are generated by sampling from the latent space distribution. D) UMAP projection of P. aeruginosa gene expression data from the real dataset (pink) and the simulated compendium using the workflow in C (grey).

Figure 2. Results of simulating compendia. A) workflow describing how experiment-specific noise was added to the simulated compendia and how the noisy simulated compendia were evaluated for similarity compared to the unpartitioned simulated compendia. B,D) SVCCA curve measuring the similarity between a compendia without noise versus a compendium with noise (light blue), compendium with noise corrected for (dark blue). As a negative control, we used the similarity between the gene expression pattern of the simulated data with a single partition compared with the simulated data that has been permuted to destroy any meaningful structure in the data. C,E) Subsampled gene expression data (500 samples per compendia) projected onto the first two principal components showing the overlap in structure between the compendia without noise (gray) versus the compendia with noise (light blue), compendia with noise corrected for (dark blue).

Figure 3. Simulating gene expression compendia by experiment. A) Workflow to simulate gene expression per experiment. B) UMAP projection of P. aeruginosa gene expression data highlighting a single experiment, E-GEOD-51409, (red) in the original dataset (left) and the simulated dataset (right), which was subsampled to 1000 samples. C) Differential expression analysis of experiment E-GEOD-51409 (left), random simulated samples (middle), simulated samples using the same experiment as a template (right). D) Number of differentially expressed genes identified across 100 simulated experiments generated using experiment-level simulation and sample-level simulation. E) Number of enriched pathways identified across 100 simulated experiments generated using experiment-level simulation and sample-level simulation.

Figure 4. Results of simulating compendia comprised of gene expression experiments. A) workflow describing how experiment-specific noise was added to the simulated compendia and how the noisy simulated compendia were evaluated for similarity compared to the unpartitioned simulated compendia. B,D) SVCCA curve measuring the similarity between a compendia without noise versus a compendium with noise (light blue), compendium with noise corrected for (dark blue). As a negative control, we used the similarity between the gene expression pattern of the simulated data with a single partition compared with the simulated data permuted to destroy any meaningful structure in the data. C,E) Subsampled gene expression data (500 samples per compendia) projected onto the first two principal components showing the overlap in structure between the compendia without noise (gray) versus the compendia with noise (light blue), compendia with noise corrected for (dark blue).

Figure S1. Simulating recount2 gene expression data using VAE. A) Validation loss plotted per epoch during training. B) UMAP projection of gene expression data from the real dataset (pink) and the simulated compendium using the workflow in Figure 1C (grey).

Figure S2. Results of varying the magnitude of the experiment-specific noise added to each partition. SVCCA curve measuring the similarity between a compendia without noise versus a compendium with noise (light blue), compendium with noise corrected for (dark blue). As a negative control, we used the similarity between the gene expression pattern of the simulated data with a single partition compared with the simulated data permuted to destroy any meaningful structure in the data. Using noise model with A) 0.2 variance, B) 0.05 variance with a zoomed in view on the left, C) 0.025 variance with a zoomed in view on the left.

Figure S3. Results of simulating *P. aeruginosa* compendia using A) sample-level simulation or B) experiment-level simulation with COMBAT noise correction.

Figure S4. Clustering of 100 random gene expression profiles in original versus simulated experiments using A) E-GEOD-21704 and B) E-GEOD-10030 as templated.

Figure S5. Results of simulating compendia with fixed number of experiments. A) workflow describing how each compendia is designed to have a fixed number of experiments, experiment-specific noise was added to the simulated compendia and how the noisy simulated

compendia were evaluated for similarity compared to the unpartitioned simulated compendia. B) SVCCA curve measuring the similarity between a compendia without noise versus a compendium with noise (light blue), compendium with noise corrected for (dark blue). As a negative control, we used the similarity between the gene expression pattern of the simulated data with a single partition compared with the simulated data that has been permuted to destroy any meaningful structure in the data. C) Subsampled gene expression data (fewer than 500 samples per compendia) projected onto the first two principal components showing the overlap in structure between the compendia without noise (gray) versus the compendia with noise (light blue), compendia with noise corrected for (dark blue).

References:

1. Perou CM. Show me the data! *Nat Genet*. 2001;29(4):373. doi:10.1038/ng1201-373
2. Tan J, Hammond JH, Hogan DA, Greene CS. ADAGE-Based Integration of Publicly Available Pseudomonas aeruginosa Gene Expression Data with Denoising Autoencoders Illuminates Microbe-Host Interactions. *mSystems*. 2016;1(1). doi:10.1128/mSystems.00025-15
3. Tan J, Doing G, Lewis KA, et al. Unsupervised Extraction of Stable Expression Signatures from Public Compendia with an Ensemble of Neural Networks. *Cell Syst*. 2017;5(1):63-71.e6. doi:10.1016/j.cels.2017.06.003
4. Chen L, Cai C, Chen V, Lu X. Learning a hierarchical representation of the yeast transcriptomic machinery using an autoencoder model. *BMC Bioinformatics*. 2016;17 Suppl 1(Suppl 1):9. doi:10.1186/s12859-015-0852-1
5. Zhou W, Altman RB. Data-driven human transcriptomic modules determined by independent component analysis. *BMC Bioinformatics*. 2018;19(1):327. doi:10.1186/s12859-018-2338-4
6. Taroni JN, Grayson PC, Hu Q, et al. MultiPLIER: a transfer learning framework for transcriptomics reveals systemic features of rare disease. *Cell Syst*. 2019;8(5):380-394.
7. Leek JT, Storey JD. Capturing heterogeneity in gene expression studies by surrogate variable analysis. *PLoS Genet*. 2007;3(9):1724-1735. doi:10.1371/journal.pgen.0030161
8. Renard E, Absil PA. Comparison of batch effect removal methods in the presence of correlation between outcome and batch. 2017.
9. Tseng GC, Oh M-K, Rohlin L, Liao JC, Wong WH. Issues in cDNA microarray analysis: quality filtering, channel normalization, models of variations and assessment of gene effects. *Nucleic Acids Res*. 2001;29(12):2549-2557. doi:10.1093/nar/29.12.2549
10. Kerr MK, Martin M, Churchill GA. Analysis of variance for gene expression microarray data. *J Comput Biol*. 2000;7(6):819-837.
11. Chen C, Grennan K, Badner J, et al. Removing batch effects in analysis of expression microarray data: an evaluation of six batch adjustment methods. *PLoS One*. 2011;6(2):e17238-e17238. doi:10.1371/journal.pone.0017238
12. Johnson WE, Li C, Rabinovic A. Adjusting batch effects in microarray expression data using empirical Bayes methods. *Biostatistics*. 2007;8(1):118-127. doi:10.1093/biostatistics/kxj037
13. Stegle O, Parts L, Piipari M, Winn J, Durbin R. Using probabilistic estimation of expression residuals (PEER) to obtain increased power and interpretability of gene expression analyses. *Nat Protoc*. 2012;7(3):500.
14. Taroni JN, Greene CS. Cross-platform normalization enables machine learning model training on microarray and RNA-Seq data simultaneously. *bioRxiv*. January 2017:118349. doi:10.1101/118349
15. Parrish RS, Spencer III HJ, Xu P. Distribution modeling and simulation of gene expression data. *Comput Stat Data Anal*. 2009;53(5):1650-1660.
16. Singhal S, Kyvernitis CG, Johnson SW, Kaiser LR, Liebman MN, Albelda SM. Microarray data simulator for improved selection of differentially expressed genes. *Cancer Biol Ther*. 2003;2(4):383-391.
17. Law CW, Chen Y, Shi W, Smyth GK. voom: Precision weights unlock linear model analysis tools for RNA-seq read counts. *Genome Biol*. 2014;15(2):R29.
18. Pimentel H, Bray NL, Puente S, Melsted P, Pachter L. Differential analysis of RNA-seq incorporating quantification uncertainty. *Nat Methods*. 2017;14(7):687.
19. Casey S. Greene, Dongbo Hu, Richard W. W. Jones, Stephanie Liu, David S. Mejia, Rob Patro, Stephen R. Piccolo, Ariel Rodriguez Romero, Hirak Sarkar, Candace L. Savonen, Jaclyn N. Taroni, William E. Vauclain, Deepashree Venkatesh Prasad KGW. refine.bio: a resource of uniformly processed publicly available gene expression datasets.

20. Leinonen R, Sugawara H, Shumway M, Collaboration INSD. The sequence read archive. *Nucleic Acids Res*. 2010;39(suppl_1):D19-D21.
21. Edgar R, Domrachev M, Lash AE. Gene Expression Omnibus: NCBI gene expression and hybridization array data repository. *Nucleic Acids Res*. 2002;30(1):207-210.
22. Brazma A, Parkinson H, Sarkans U, et al. ArrayExpress—a public repository for microarray gene expression data at the EBI. *Nucleic Acids Res*. 2003;31(1):68-71.
23. Kingma DP, Welling M. Auto-encoding variational bayes. *arXiv Prepr arXiv13126114*. 2013.
24. McInnes L, Healy J, Melville J. Umap: Uniform manifold approximation and projection for dimension reduction. *arXiv Prepr arXiv180203426*. 2018.
25. Ritchie ME, Phipson B, Wu D, et al. limma powers differential expression analyses for RNA-sequencing and microarray studies. *Nucleic Acids Res*. 2015;43(7):e47-e47.
26. Raghu M, Gilmer J, Yosinski J, Sohl-Dickstein J. Svcca: Singular vector canonical correlation analysis for deep learning dynamics and interpretability. In: *Advances in Neural Information Processing Systems*. ; 2017:6076-6085.
27. Barbier M, Damron FH, Bielecki P, et al. From the environment to the host: re-wiring of the transcriptome of Pseudomonas aeruginosa from 22°C to 37°C. *PLoS One*. 2014;9(2):e89941-e89941. doi:10.1371/journal.pone.0089941
28. Powers RK, Goodspeed A, Pielke-Lombardo H, Tan A-C, Costello JC. GSEA-InContext: identifying novel and common patterns in expression experiments. *Bioinformatics*. 2018;34(13):i555-i564. doi:10.1093/bioinformatics/bty271
29. Crow M, Lim N, Ballouz S, Pavlidis P, Gillis J. Predictability of human differential gene expression. *Proc Natl Acad Sci*. 2019;116(13):6491 LP - 6500. doi:10.1073/pnas.1802973116
30. Tralau T, Vuilleumier S, Thibault C, Campbell BJ, Hart CA, Kertesz MA. Transcriptomic analysis of the sulfate starvation response of Pseudomonas aeruginosa. *J Bacteriol*. 2007;189(19):6743-6750.
31. Espín-Pérez A, Portier C, Chadeau-Hyam M, van Veldhoven K, Kleinjans JCS, de Kok TMCM. Comparison of statistical methods and the use of quality control samples for batch effect correction in human transcriptome data. *PLoS One*. 2018;13(8):e0202947-e0202947. doi:10.1371/journal.pone.0202947
32. Collado-Torres L, Nellore A, Kammers K, et al. Reproducible RNA-seq analysis using recount2. *Nat Biotechnol*. 2017;35(4):319-321. doi:10.1038/nbt.3838
33. Weinstein JN, Collisson EA, Mills GB, et al. The Cancer Genome Atlas Pan-Cancer analysis project. *Nat Genet*. 2013;45(10):1113-1120. doi:10.1038/ng.2764
34. Consortium Gte. The Genotype-Tissue Expression (GTEx) pilot analysis: multitissue gene regulation in humans. *Science (80-)*. 2015;348(6235):648-660.
35. Abadi M, Agarwal A, Barham P, et al. Tensorflow: Large-scale machine learning on heterogeneous distributed systems. *arXiv Prepr arXiv160304467*. 2016.
36. Chollet F. No Title.
37. Way GP, Greene CS. Extracting a biologically relevant latent space from cancer transcriptomes with variational autoencoders. *Pac Symp Biocomput*. 2018;23:80-91.
38. Smyth Gordon K. Linear models and empirical bayes methods for assessing differential expression in microarray experiments. *Stat Appl Genet Mol Biol*. 2004;3(1):1-25.
39. Yu G, Wang L-G, Han Y, He Q-Y. clusterProfiler: an R package for comparing biological themes among gene clusters. *Omi a J Integr Biol*. 2012;16(5):284-287.
40. Lee AJ; Park Y; Doing G; Hogan DA; Greene CS (2020): Supporting data for "Correcting for experiment-specific variability in expression compendia can remove underlying signals" GigaScience Database. http://dx.doi.org/10.5524/100796

Figure 1

Click here to access/download;Figure;Fig1.png

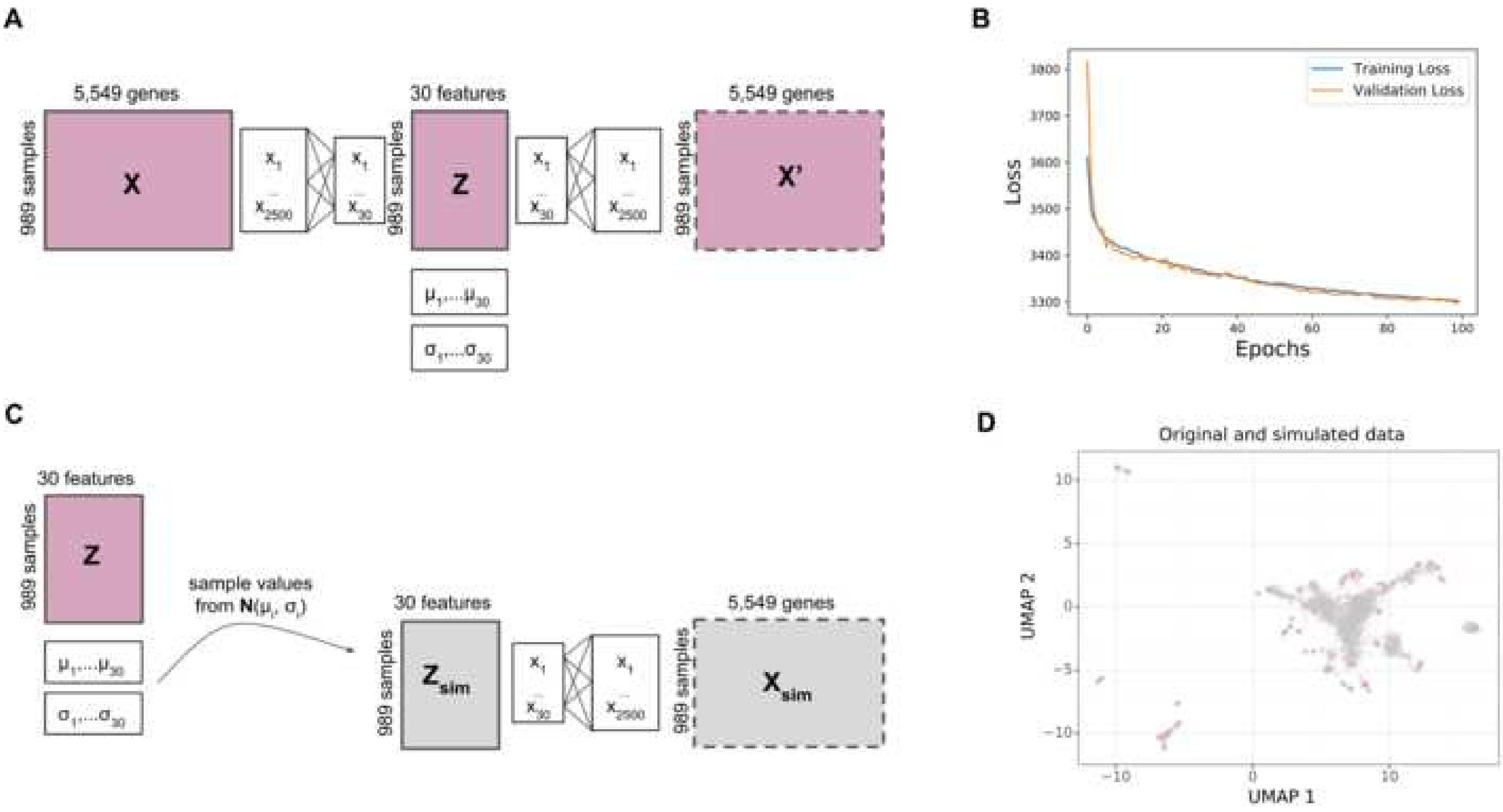

Figure 2

Click here to access/download;Figure;Fig2.png

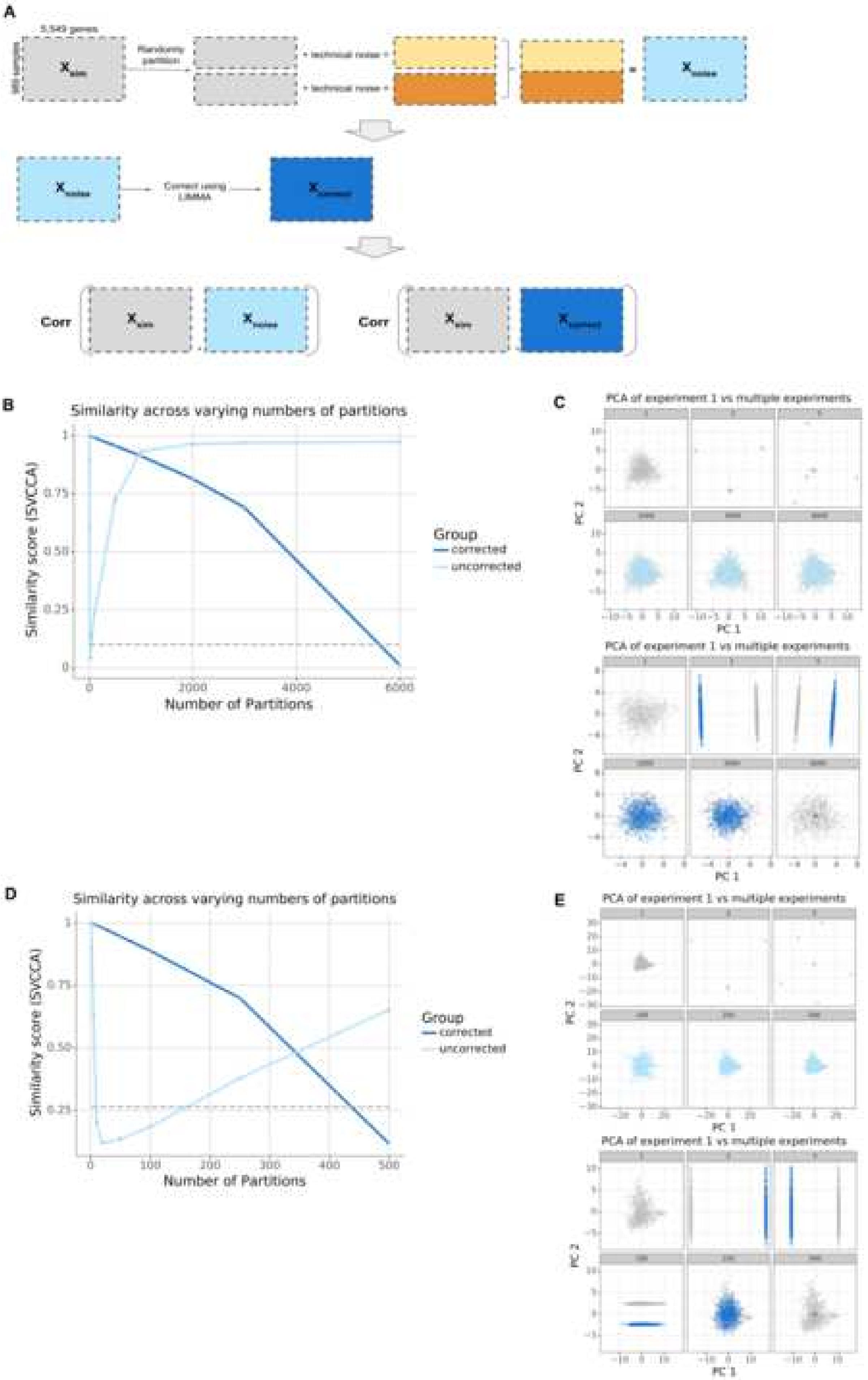

Figure 3

Click here to access/download;Figure;Fig3_revised.png

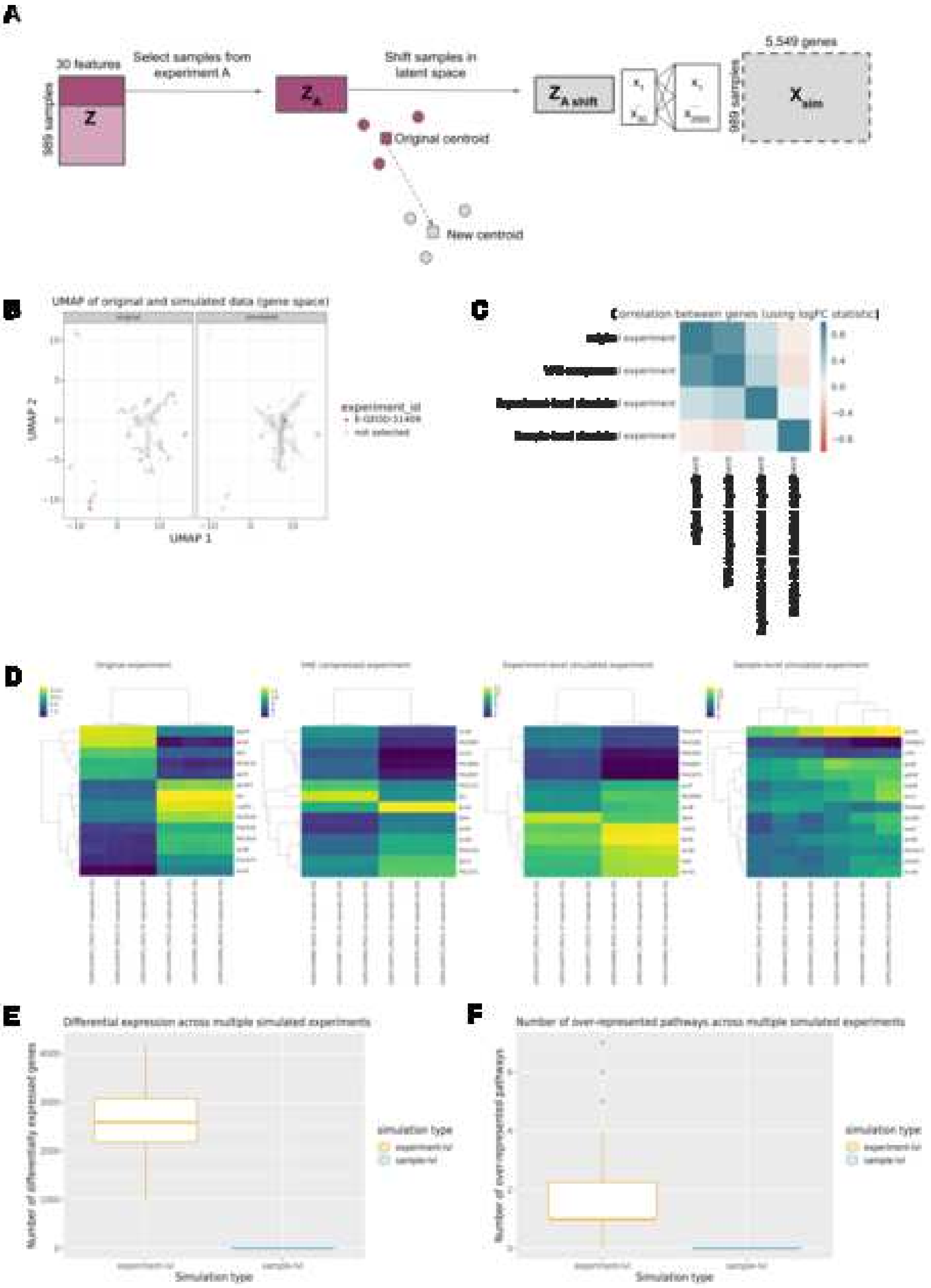

Figure 4

Click here to access/download;Figure;Fig4.png

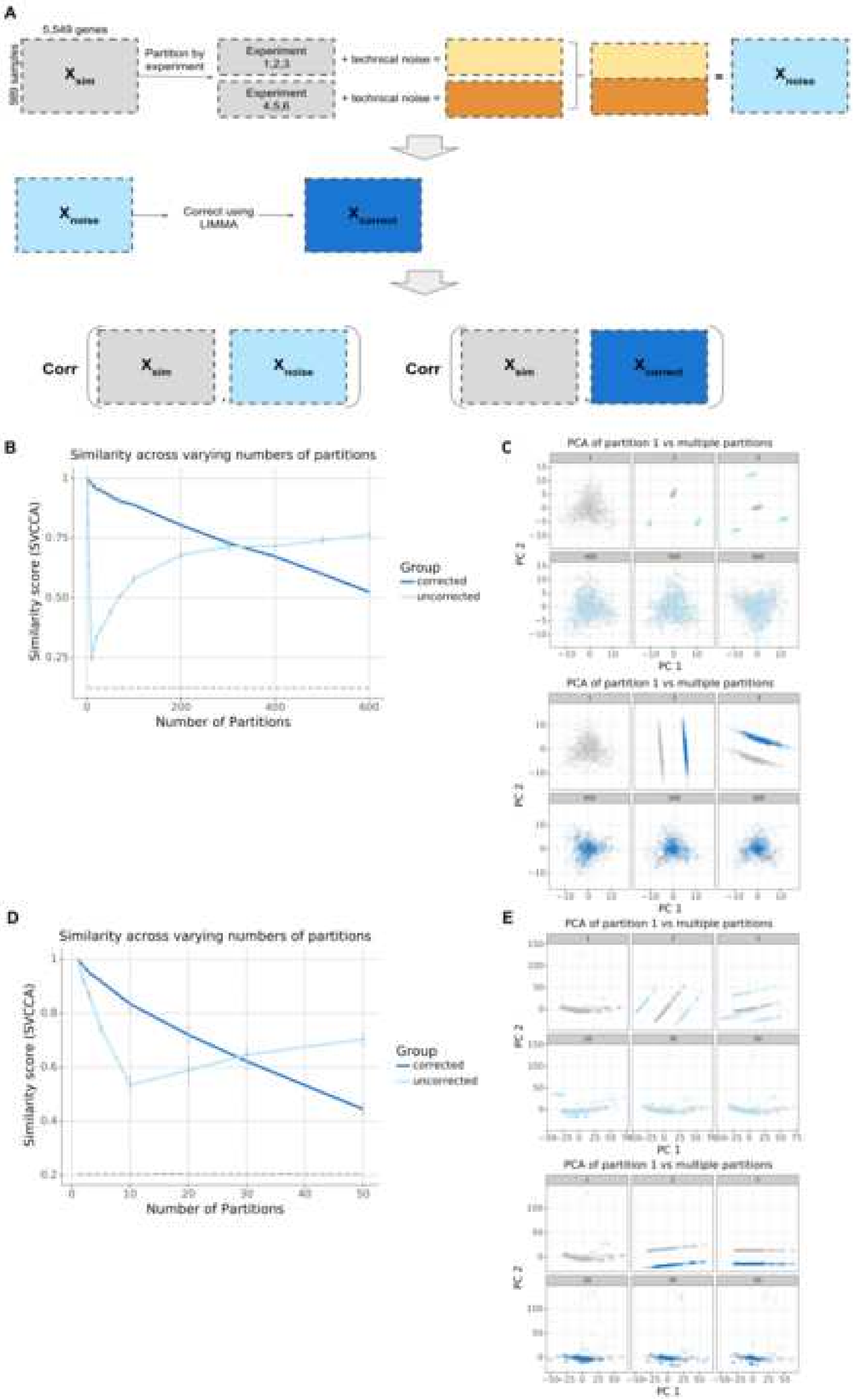

Supplementary Figure 1

Click here to access/download

Supplementary Material

Supp1.png

Supplementary Figure 2

Click here to access/download
Supplementary Material
Supp2.png

Supplementary Figure 3

Click here to access/download
Supplementary Material
Supp3.png

Supplementary Figure 4

Click here to access/download
Supplementary Material
Supp4.png

Supplementary Figure 5

Click here to access/download
Supplementary Material
Supp5.png

Response to Reviewers Click here to access/download;Personal Cover;Reviewer comments (1).pdf

Dear Dr. Edmunds,

We want to thank the reviewers for taking the time to read and give feedback on this manuscript. All the comments and suggestions were very helpful. We think they greatly improve the quality of the manuscript. We tracked revisions in the manuscript document that we uploaded. We addressed every comment in the point-by-point responses that follow. Before we get to that, there were two major changes suggested by the reviewers that led us to perform new analyses. The first was suggested by both reviewers. They observed that while the main claim of the paper was that increasing the number of partitions (noise) allows us to recover our original signal without correction. In this setting the size of partitions is not constant (partitions shrink as the number goes up). In order to test the effect of fixed size partitions (individual experiments), we performed a new analysis where we generate experiments without holding the total compendium size constant.

We found that with few experiments in a compendium, the main signal was the difference between experiments. adding noise to each experiment drives signal detection down. Additionally, applying noise correction removed the main experiment-specific signal, as it was designed to do, but in this case the technical noise is perfectly confounded with experiment-to-experiment differences, so applying noise correction will consistently remove more of the signal of interest. The results of this analysis exemplifies how existing experiments can be combined and used without need for correction - consistent with our previous findings. We found this analysis interesting, but we expect that our findings are most relevant in the case of fixed-size compendia (i.e., what one would download from public repositories). We have added this result to the paper and supplement.

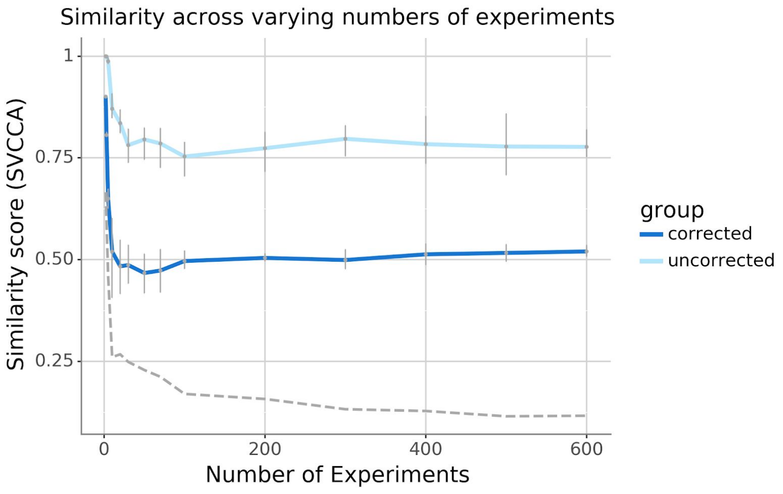

The second major change was brought up by Reviewer 2. In the original manuscript, we presented differential expression (DE) analysis using the original experiment, VAE compression and latent space transformed experiment (experiment-level), and VAE with random sampled experiment (sample-level). We now realize that combining two processes into one (adding both

the VAE and latent space transformation) was particularly confusing. We realized that adding an additional panel to Figure 3 would help to make the experiment much clearer. We revised Figure 3 and the associated text to present the DE analysis using the original experiment, VAE compressed only experiment, VAE compressed and latent space translated experiment (experiment-level), VAE compressed and random sampled experiment (sample-level). We then examined the retention of the original differential expression signature by comparing the set of differentially expressed genes (DEGs) found in the original experiments versus the other simulated experiments. Applying only the VAE compression to the original experiment, generated an experiment that had the same sample grouping as the original. However, only a subset of the differentially expressed genes found in the VAE compressed were also found in the original experiment. We found that the correlation between genes, based on log fold change values, is high (R^2 = 0.822) between the original and the VAE compressed only experiment as expected. The VAE compression step slightly alters the set of differentially expressed genes since the data is being compressed into a low dimensional space. Next, the original samples in an experiment and the VAE compressed and latent space translated simulated experiment have consistent clustering of samples (experiment-level simulated experiment). However the genes that were differentially expressed were different between the two experiments. The correlation between genes in the original and the experiment-level experiment are lower, R^2 = 0.230, since they ideally represent unique experiments with an identical design. The residual similarity is likely due to commonly differentially expressed genes that have been observed previously (Powers et. al. 2018, Crow et. al. 2019). Finally, the original experiment structure is not well preserved using the VAE compressed and random sampling approach (sample-level simulated experiment). The correlation between genes in the original and sample-level experiment is non-existent, R^2 = -0.055, since this simulation does not account for any experiment structure.

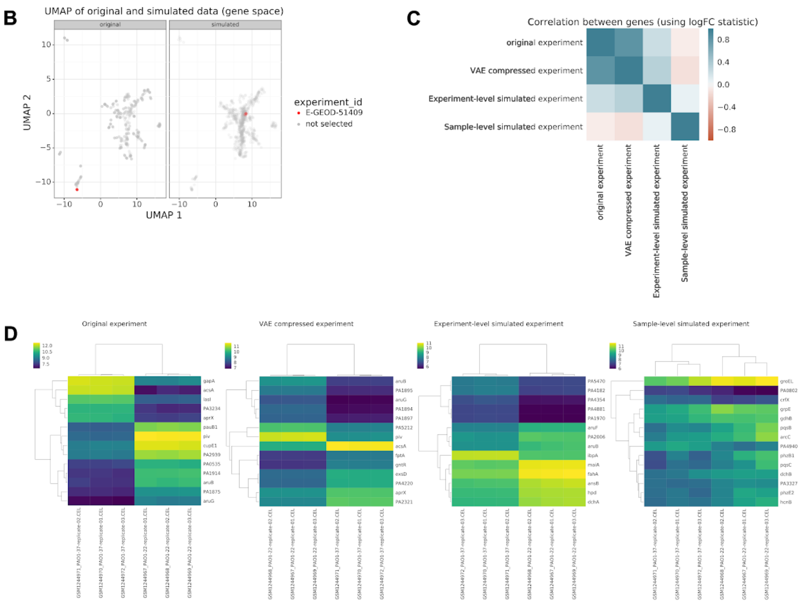

Sincerely,
Casey

POINT BY POINT RESPONSE

Reviewer #1

1. The figures S1 and S2 were switched.
    a. Thank you for noticing this. We have corrected the labels of the figure captions on page 22 of the revised manuscript so that the figure caption matches the appropriate figure.
2. The authors should also cite PEER (PMID: 22343431), which is currently one of the most widely used package to correct for experimental noises.
    a. Thank you for the suggestion. We have added this new citation to the introduction. The sentence with this citation states: "*Numerous methods have been designed to correct for various types of effects*." (page 3)
3. It would be interesting if the authors could explore the influence of experiment numbers in the noise effects.
    a. We showed that with increasing the number of partitions (noise) we can recover our original signal without correction. However, as both reviewers point out, the effect of the number of partitions is confounded by the number of experiments or samples per partition since more partitions equated to each partition having fewer samples or experiments. We introduced a new analysis where we held the partition size fixed. The approach for this new analysis is described in the new "Experiment-effect analysis" section of the methods (page 16): "*First, we used the experiment-based simulation approach to simulate P. aeruginosa compendia with [2, 3, 5, 10, 20, 30, 50, 70, 100, 200, 300, 400, 500, 600] experiments. Next, we divided the simulated compendium into the same number of partitions so that there was one experiment per partition. For each partition we added simulated noise as described in the previous section. Finally we used SVCCA to compare the noisy compendia with X number experiments with the unpartitioned compendia with X number of experiments. We also used SVCCA to compare the noise-corrected compendia with X experiments with the unpartitioned compendia with X experiments*." We added the following text to "*Simulating gene expression compendia with synthetic experiments*" section of the results (page 10): "*With few experiments in a compendia, the main signal is the difference between experiments so adding noise to each experiment drives signal detection down. Additionally, applying noise correction removed the main experiment-specific signal, as it was designed to do. With more experiments in a compendia, we gain a more global gene expression representation, where the main signal is no longer focused on the difference between experiments. Thus, adding noise to each experiment does not affect our signal detection and our similarity remains constant. However, applying noise correction will consistently remove more of our signal of interest. The results of this analysis exemplifies how existing experiments can be combined and used without need for correction.*" A new supplementary figure, S5, has been added with the results of the additional analysis

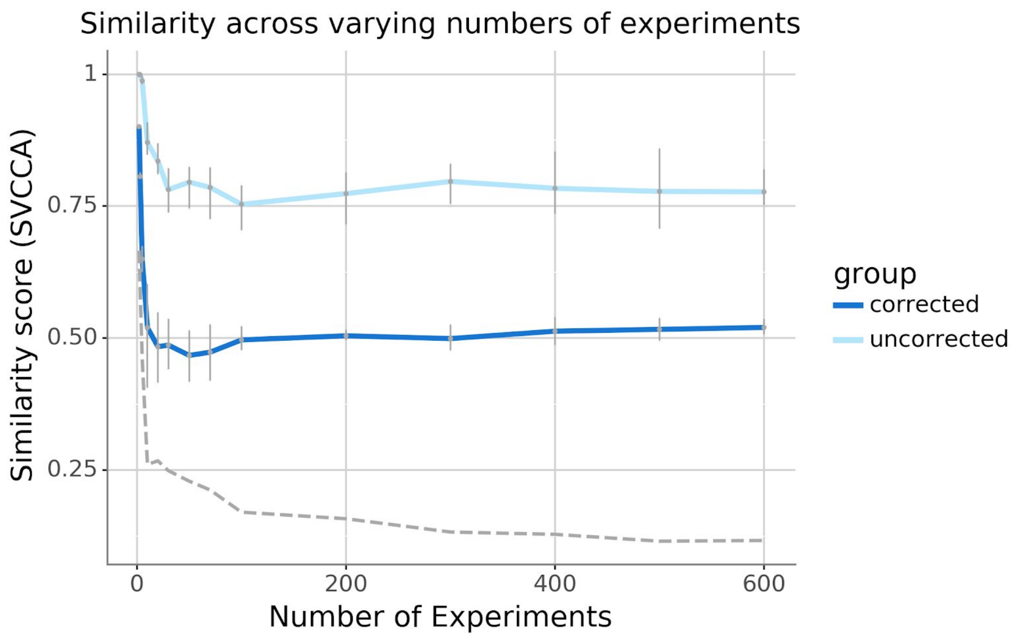

b.

4. Much of the description regarding the analyses and discussions were included in Methods part, the authors might consider to move some of the text to Results and Discussion to facilitate the understanding of readers.
    a. This is a very good point. We have moved some of the statements that were originally in the methods section into the discussion and results section in the revised manuscript. Some of the sections that were moved include: moving the explanation for the model limitation to the discussion section, moving details about the number of partitions to the results section, moving the intuition behind the simulations to the results.

Reviewer #2

Major revisions:

1. In gene expression analysis, the objective is commonly to seek for differentially expressed genes and gene co-expression networks. The experiments should be designed to investigate these relevant signals rather than only compare the structure with original data. In single experiment-based simulation, the paper had some work on differentially expressed genes and enriched pathways in Figure 3C and Table 2, but the results look a little bit weird. There were 505 and 14 differentially expressed genes found in the original experiment and experiment-simulated data, where many signals were removed in VAE-based simulation. Also, in Figure 3C, I found the top differentially expressed genes for original data and experiment-level simulation data are quite different. The author concluded "Repeating this process for each experiment allowed us to generate new simulated compendia comprised of realistic experimental designs.", but I don't think it is correct if their differentially expressed genes are not from the same pool. For simulating multiple synthetic experiments, I do suggest to add another set of experiments to evaluate the similarity between corrected and uncorrected data by

calculating the proportion of differentially expressed genes or strong gene co-expression connections from original data can be captured. E.g. collecting several experiments from the same disease with both patients and controls. I think it is a more practical scenario in biomedical research.

a. In the original manuscript, we presented DE analysis using the original experiment, VAE compression and latent space transformed experiment (experiment-level), and VAE with random sampled experiment (sample-level). It seems there was a misunderstanding and this reviewer thought that we presented the DE results using the original experiment, VAE compression only experiment, VAE random sampled experiment (sample-level simulation). We apologize for the confusion and realized that adding an additional panel to Figure 3 would help to make the logic more clear. We revised Figure 3 and the associated text to present the DE analysis using the original experiment, VAE compressed only experiment, VAE compressed and latent space translated experiment (experiment-level), VAE compressed and random sampled experiment (sample-level). We then examined the retention of the original differential expression signature by comparing the set of differentially expressed genes (DEGs) found in the original experiments versus the other simulated experiments. Applying only the VAE compression to the original experiment, generated an experiment that had the same sample grouping as the original. However, only a subset of the differentially expressed genes found in the VAE compressed were also found in the original experiment. We found that the correlation between genes, based on log fold change values, is high (R^2 = 0.822) between the original and the VAE compressed only experiment as expected. The VAE compression step adds some noise to the expression signal in the original experiment, as expected, since the data is being compressed into a low dimensional space. Next, the original samples in an experiment and the VAE compressed and latent space translated simulated experiment have consistent clustering of samples (experiment-level simulated experiment). However the genes that were differentially expressed were different between the two experiments. The correlation between genes in the original and the experiment-level experiment are lower, R^2 = 0.230, since they represent unique experiments. The residual similarity is likely due to commonly differentially expressed genes that have been observed previously[1],[2]. Finally, the original experiment structure is not well preserved using the VAE compressed and random sampling approach (sample-level simulated experiment). The correlation between genes in the original and sample-level experiment is non-existent, R^2 = -0.055, since we did not account for experiment structure in the sample-level simulation.

---

[1] "Predictability of human differential gene expression | PNAS." 7 Mar. 2019, https://www.pnas.org/content/116/13/6491. Accessed 28 Aug. 2020.

[2] "GSEA-InContext: identifying novel and common patterns in ...." 27 Jun. 2018, https://academic.oup.com/bioinformatics/article/34/13/i555/5045793. Accessed 28 Aug. 2020.

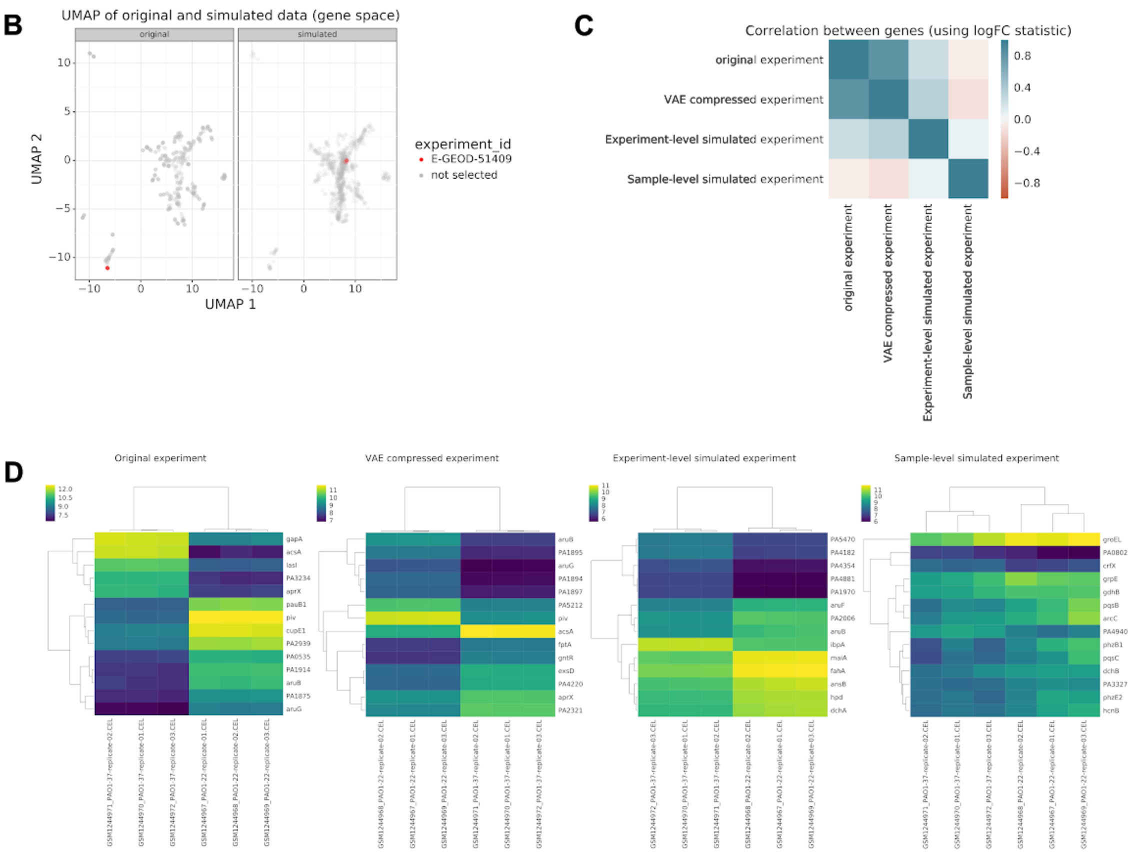

b.

2. With a large number of partitions, I am not surprised the correlation is low between the original data and corrected simulated data. Besides the number of partitions, the sample size per partition is another factor influence batch effect correction. E.g. Based on the information from Table 1, if we assume the no. samples per partition is 10 and one partition corresponding to one experiment, before the partition ca. 350 (in Figure 2D), the corrected data is better than the uncorrected one. We have the experiments by fixing sample size and changing the partition number. And I wonder if the no. samples per partition is fixed (e.g 10), what happens when the number of partition increases? Also, the conclusion should be more precise with the information for both number of partition and sample size.
    a. This is a very good suggestion. Both reviewers have asked this question. We designed an experiment, also described in response to the first reviewer 1.3, to address this. As noted above, “We showed that with increasing the number of partitions (noise) we can recover our original signal without correction. However, as both reviewers point out, the effect of the number of partitions is confounded by the number of experiments or samples per partition since more partitions equated to each partition having a smaller effect size (i.e. each partition having fewer samples or experiments). We introduced a new analysis where we held the partition size fixed. First, we used the experiment-based simulation approach (defined in Figure 3A) to simulate *P. aeruginosa* compendia with varying number

of experiments (compendia with [2, 3, 5, 10, 20, 30, 50, 70, 100, 200, 300, 400, 500, 600] experiments). Next, we divided the simulated compendium into the same number of partitions so that there was one experiment per partition. For each partition we added simulated noise as described in the previous section. Finally we used SVCCA to compare the noisy compendia with X number experiments with the unpartitioned compendia with X number of experiments. We also used SVCCA to compare the noise-corrected compendia with X experiments with the unpartitioned compendia with X experiments. With few experiments in a compendia, the main signal is the difference between experiments so adding noise to each experiment drives signal detection down. Additionally, applying noise correction removed the main experiment-specific signal, as it was designed to do. With more experiments in a compendia, we gain a more global gene expression representation, where the main signal is no longer focused on the difference between experiments. Thus, adding noise to each experiment does not affect our signal detection and our similarity remains constant. However, applying noise correction will consistently remove more of our signal of interest. The results of this analysis exemplifies how existing experiments can be combined and used without need for correction."

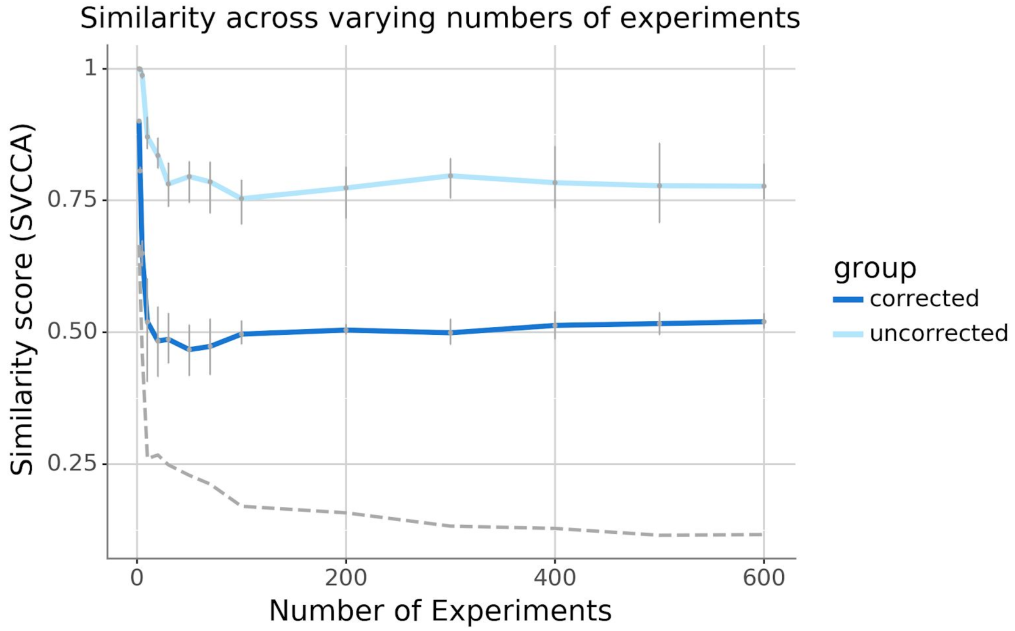

b.

3. Most parts of this paper are well-written, but the structure of several long sentences should be further revised.
    a. This is a very good suggestion. We have rewritten some of the previously long sentences to be shorter to hopefully make reading the manuscript smoother.

Minor revisions:

4. For the VAE model, how to determine the number of hidden layers and hidden nodes? Why you choose 30 latent space features finally? Do the users need GPU to run the simulation codes?

    a. This is a good suggestion. We have added text to explain the process by which we chose the neural network architecture. The added text reads: "*A similar assessment was performed to determine the neural network architecture. We manually inspected the validation loss using multiple different 2-layer designs (300-10, 2500-10, 2500-20, 2500-30, 2500-100, 2500-300) and found 2,500 layer to 30 hidden layer VAE to be most optimal.*"(page 14 of the revised manuscript). Overall, we used the validation loss of the trained VAE as our metric to determine which set of parameters, including neural network architecture, to select.
    b. A GPU was not required to run these simulations. We have added the following text to the *implementation and software availability* section: "*All simulations were run on a CPU.*"(page 20)
5. Page 5, "We evaluated the training and validation set loss at each epoch, which stabilized after roughly 100 epochs (Figure 1B)". Although the authors mentioned the dataset they used in Figure legend, but not in the text.
    a. We have added the size of the datasets used for training to the text on page 5 of the revised manuscript. The added text states: "*We trained VAEs for each dataset: recount2 (896 samples with 58,037 genes) and P. aeruginosa(989 samples with 5,549 genes).* "
6. Page 7, "We used UMAP to visualize the structure of the original and data…", data refers to simulated data?
    a. You're correct in your interpretation of the text. We have added "simulated" to this sentence to clarify which datasets are being compared (see page 5 of the revised manuscript).
7. The authors should involve some key numbers in the result section, such as the number of simulated samples, the list of number of partitions etc.
    a. We have added the number of simulated samples and partitions to both the "*Simulating gene expression compendia with synthetic samples*" and "*Simulating gene expression compendia with synthetic experiments"* sections.
8. In Methods section, delete "The P. aeruginosa dataset was previously processed by Tan et. al.2 ", this have been mentioned two times before.
    a. The additional citation has been deleted in the revised manuscript (page 12)
9. Page 13, "58,037 gene". This is not for "gene", using "transcript" is more precise
    a. Thank you for checking this detail of the manuscript. We have confirmed that these numbers refer to genes instead of transcripts. Recount's website (https://jhubiostatistics.shinyapps.io/recount/) states in the documentation (which we cannot directly link because of the way the shiny app is constructed): "*The RangedSummarizedExperiment object for the counts summarized at the gene level using the Gencode v25 (GRCh38.p7, CHR) annotation as provided by Gencode*." When we downloaded the *Comprehensive gene annotation Gencode v25 GTF* file from this link: https://www.gencodegenes.org/human/release_25.html, we get 58,037 genes, which includes pseudogenes, lncRNAs, snRNA, and other types in addition to protein coding genes.

10. Page14. I suggest to divide the "Constructing a generative model of gene expression compendia" into three sections, 1. The strategy to construct VAE and define its structure and hyperparameters, 2. Sample-based simulation; 3. Experiment-based simulation
    a. Thank you for the helpful suggestion. We have divided the "*Constructing a generative model of gene expression compendia*" into the three suggested sections (page 14-16 of the revised manuscript)
11. Page 14, move "Though linear noise is an over-simplification of the types…" to discussion.
    a. This sentence has been moved to the discussion section on page 11 of the revised manuscript.
12. Page 9. "We exampled how the original samples in an experiment (E-GEOD- 51409)…. However the genes that were differently expressed were different between the two datasets." This sentence should be revised.
    a. This sentence has been updated to reflect the additional experiment described in 1.3 and 2.2.

Manuscript with Tracked Changes

Click here to access/download;Personal Cover;compendium_manuscript_revised.docx

Correcting for experiment-specific variability in expression compendia can remove underlying signals

Alexandra J. Lee[1,2], YoSon Park[2], Georgia Doing[3], Deborah A. Hogan[3], Casey S. Greene[2,4]

[1] Genomics and Computational Biology Graduate Program, University of Pennsylvania, Philadelphia, PA, USA
[2] Department of Systems Pharmacology and Translational Therapeutics, University of Pennsylvania, Philadelphia, PA, USA
[3] Department of Microbiology and Immunology, Geisel School of Medicine, Dartmouth, Hanover, NH, USA
[4] Childhood Cancer Data Lab, Alex's Lemonade Stand Foundation, Philadelphia, PA, USA

Abstract:

Motivation: In the last two decades, scientists working in different labs have assayed gene expression from millions of samples. These experiments can be combined into compendia and analyzed collectively to extract novel biological patterns. Technical variability, sometimes referred to as batch effects, may result from combining samples collected and processed at different times and in different settings. Such variability may distort our ability to interpret and extract true underlying biological patterns. As more ~~multi-experiment,~~ integrative analysis methods are developed and available data collections are increased in size, it is crucial to determine how technical variability affect our ability to detect desired patterns when many experiments are combined

Objective: We sought to determine the extent to which an underlying signal was masked by technical variability by simulating compendia comprised of data aggregated across multiple experiments.

Method: We developed a generative multi-layer neural network to simulate compendia of gene expression experiments from large-scale microbial and human datasets. We compared simulated compendia before and after introducing varying numbers of sources of undesired variability.

Results: We found that the signal from a baseline compendium was obscured when the number of added sources of variability was small. Perhaps as expected, applying statistical correction methods rescued the underlying signal in these cases. However, ~~A~~as the number of sources of variability increased~~, surprisingly,~~ we observed that detecting the original signal became increasingly easier even without correction. In fact, applying statistical correction methods reduced our power to detect the underlying signal.

Conclusion: When combining a modest number of experiments, it is best to correct for experiment-specific noise. However, when many experiments are combined, statistical correction reduces ~~one's~~ our ability to extract underlying patterns.

Introduction:

Over~~For~~ the last two decades, unprecedented amounts of transcriptome-wide gene expression profiling data have been generated. ~~, m~~Most of these datasets~~which~~ are shared in public platforms for the research community.[1] Researchers are now combining samples across different experiments to form compendia, and analyzing these compendia is revealing new biology.[2–6] It is well-understood that technical sources of variability pervade large-scale data analysis such as transcriptome-wide expression profiling studies.[7–10] Numerous methods have been designed to correct for various types of effects.[7,11–13] Despite the prevalence of technical sources of variability, researchers have successfully extracted biological patterns from multi-experiment compendia without applying correction methods.[2–5,14] ~~We sought t~~To determine the basis of these seemingly contradictory results, we ~~by~~ examined~~ing~~ the extent to which underlying statistical structure can be extracted from compendium-style datasets in the presence of sources of undesired variability.

A number of methods have been developed to simulate transcriptome-wide expression experiments.[15–18] However, ~~simulating a compendium of many experiments with~~these existing approaches ~~would~~ require defining a statistical model that describes the~~-~~ process by which researchers design and carry out experiments, which is often ~~likely to be~~ very challenging to obtain. Instead, we developed an approach to simulate compendia by sampling from the low-dimensional representation produced by multi-layer generative neural networks trained on gene expression data from an existing compendium. This allowed us to simulate gene expression experiments that mimic real experimental configurations. We combined these experiments to create compendia.

Using this simulation approach, we studied how adding varying amounts of experiment-specific noise affects ~~the statistical structure of gene expression compendia and~~ our ability to detect underlying patterns in the gene expression compendia. This topic is becoming pressing as more large-scale expression compendia are becoming~~e~~ available. We found that prior reports of pervasive technical noise and analyses that succeed without correcting for it are, in fact, consistent. In settings with relatively few experiment-specific sources of undesired variation, the added noise substantially alters the structure of the data. In these settings, statistical correction produces a data representation that better captures the original variability in the data. On the

other hand, when the number of experiment-specific sources of undesired variability is~~becomes~~ large, attempting to correct for these sources does more harm than good.

Results:

We characterized publicly available data compendia using refine.bio[19], a meta-repository that integrates data from multiple different repositories. We found that, on average, ~~an average~~ experiments contained hundreds to thousands of samples in most widely studied organisms (Table 1). These samples were derived from hundreds to thousands of experiments, and the most common experimental designs had relatively few samples (medians from 5-12). We compared compendia from refine.bio ~~these~~ to two readily available compendia, recount2 and one for *P. aeruginosa*, that have been used for compendium-wide analyses.[2,3,6] The compendia that have been successfully used in prior work[2,3,6] have similar median numbers of samples per experiment (recount2 = 4, *P. aeruginosa* = 6) to the current publicly available data.

Table 1: *Public data usually have only a modest number of samples per experiment, though ~~in aggregate~~ many samples are available in aggregate. Statistics for the 10 largest transcriptomic compendia found in refine.bio, which is a meta-repository containing publicly available expression data from the Sequence Read Archive (SRA)[20], Gene Expression Omnibus (GEO)[21] and ArrayExpress[22].*

	**No. experiments**	**Median no. samples**	**Total no. samples**
Homo s~~S~~apiens	15,440	12	571,862
Mus m~~M~~usculus	13,224	10	296,829
Arabidopsis ~~T~~thaliana	1,627	9	24,855
Rattus n~~N~~orvegicus	1,368	12	38,530
Drosophila m~~M~~elanogaster	853	9	17,836
Saccharomyces ~~C~~cerevisiae	627	12	12,972
Danio ~~R~~rerio	546	9.5	28,518
Caenorhabditis ~~E~~elegans	375	10	7,953
Sus ~~S~~scrofa	280	12	6,063
Zea ~~M~~mays	274	5	3,458

Constructing a generative model for gene expression samples

We developed an approach to simulate new gene expression compendia using generative multi-layer neural networks. Specifically, we trained a variational autoencoder (VAE)[23], which

Formatted: Font: Italic
Formatted: Font: Italic
Formatted: Left
Formatted: Font: Italic
Formatted: Font: Italic
Formatted: Left
Formatted: Font: Italic
Formatted: Font: Italic
Formatted: Left
Formatted: Font: Italic
Formatted: Font: Italic
Formatted: Left
Formatted: Font: Italic
Formatted: Font: Italic
Formatted: Left
Formatted: Font: Italic
Formatted: Font: Italic
Formatted: Left
Formatted: Font: Italic
Formatted: Font: Italic
Formatted: Left
Formatted: Font: Italic
Formatted: Font: Italic
Formatted: Left
Formatted: Font: Italic
Formatted: Font: Italic
Formatted: Left
Formatted: Font: Italic
Formatted: Font: Italic
Formatted: Left

was comprised of an encoder and decoder neural network. The encoder neural network compressed the input data through two layers into a low-dimensional representation and the decoder neural network expanded the dimensionality back to the original input size. The VAE learned a low-dimensional representation that can reconstruct the original input data. Simultaneously, the VAE optimized the lowest dimensional representation to follow a normal distribution (Figure 1A). This normal distribution constraint, which distinguishes VAE's from other types of autoencoders,~~ and~~ allowed us to generate variations of the input data by sampling from a continuous latent space.[23]

We trained VAEs for each compendium~~dataset~~: ~~(~~recount2 (896 samples with 58,037 genes) and *P. aeruginosa*~~)~~(989 samples with 5,549 genes). We evaluated the training and validation set losses at each epoch, which stabilized after roughly 100 epochs (Figure 1B). We observed a similar stabilization after 40 epochs for recount2 (Figure S1A). We simulated new genome-wide gene expression data by sampling from the latent space of the VAE using a normal distribution (Figure 1C). We used UMAP[24] to visualize the structure of the original and simulated data and found that the simulated data generally fell near original data for both compendia (Figure 1D; Figure S1B).

Simulating gene expression compendia with synthetic samples

We designed a simulation study to assess the extent to which artifactual noise associated with individual partitions of a large compendium affects the structure of the overall compendium. Our simulation is akin to asking: if different labs performing transcriptome-wide experiments randomly sampled from the available set of possible conditions, to what extent would experiment-specific biases dominate the signal of the data. First, we ~~We~~ simulated new compendia. Then we ~~,~~ randomly divided the samples within these compendia into partitions~~,~~ and ~~then~~ added noise to each partition. Finally, we~~, and~~ compared the simulated compendia with added noise to the unpartitioned one (Figure 2A). Each partition represent~~s~~eds groups of samples with shared experiment-specific noise. We evaluated the similarity ~~-~~before and after applying an algorithm designed to correct for technical noise in each partition – given that the added noise was linear, ~~noise added~~ we used limma[25] to correct. Singular Vector Canonical Correlation Analysis (SVCCA)[26] was used to assess similarity. The SVCCA analysis measured the correlation between the distribution of gene expression in the compendia without noise compared to the distribution in the compendia with multiple sources of technical variance.

Formatted: Line spacing: 1.5 lines

We performed a study with this design using the VAE trained from the *P. aeruginosa* compendium. We simulated a *P. aeruginosa* compendium with 6,000 samples for [1, 2, 5, 10, 20, 50, 100, 500, 1000, 2000, 3000, 6000] ~~2 to 6,000~~ partitions. We found that adding technical ~~noise~~ variance to partitions always reduced the similarity between the simulated data without partitions and the partitioned simulated data. However, the nature of the change in similarity differed substantially between the partitioned ~~sets~~ compendia before and after the correction step (Figure 2B). With the correction step (dark blue line) similarity dropped throughout the range of the study, eventually reaching the same level as the permuted data (dashed grey line). Without the correction step (light blue line), similarity dropped immediately to ~~near~~ the random level and then recovered throughout the rest of the tested range. We ~~V~~visualized the~~ing~~ simulated data on the top 2 principle components from the original data (Figure 2C, grey points). ~~with t~~The corrected (Figure 2C, dark blue) and uncorrected (Figure 2C, light blue) data at various numbers of partitions revealed that the correction step removes both wanted and unwanted variability, eventually removing all variability in the data. Without correction, the data were initially dramatically transformed.~~;~~ ~~h~~However, as the number of partitions grows very large the effect on the structure of the data was diminished.

To determine whether or not this correction removing signal was a more general property of such compendia, we repeated the same simulation study using a VAE trained on a recount2 compendium. recount2 is a compendium comprised of human RNA-seq samples, so it is generated using a different technology and consists of assays of a very different organism. We~~Results with recount2~~ simulated a compendium with 500 samples for [1, 2, 5, 10, 20, 50, 100, 250, 500] partitions. The results with recount2 mirrored our findings with the *P. aeruginosa* compendium. The correction step initially retained more similarity, but performance crossed over and by 500 partitions ~~the end of the study~~ the uncorrected data were more similar to the unpartitioned simulated compendium (Figure 2D). Visualizing the top principle components, again, revealed that correction ~~better retained~~restored the structure of the original data with few partitions, but with many partitions the structure was better retained without correction (Figure 2E). Additionally, the same trends ~~are~~were observed when we varied the magnitude of the noise added (Figure S2) or used~~ing~~ a different noise correction method, such as COMBAT[12] (Figure S3). In general, there exists some minimum number of experiment-specific sources of

noise that determines the effectiveness of applying noise correction to these multi-experiment compendia.

A generative model for gene expression experiments

We randomly selected samples from the range of all possible samples in the compendium. ~~For the next simulation, we developed an approach that could simulate realistic experimental structure.~~ This next simulation added another level of complexity to the model, by simulating experiments as opposed to samples~~, in order to~~to make the simulated compendia more representative of true expression data. This simulation generated synthetic experiments for which the gene expression patterns were consistent with those from the types of experiments that are used within the field. The technique that we developed uses the same underlying approach of sampling from a VAE. However, in this case

we randomly selected a template experiment (E-GEOD-51409, which compared *P. aeruginosa* at 22˚C and 37˚C) and a vector that would move that template experiment to a new location in the gene expression space (Figure 3A). The simulation preserved the relationship between samples within the template experiment while also shifting the activity of the samples in the latent space (Figure 3B). Intuitively, this process maintained the relationship between samples but changed the underlying perturbation; this simulation maintained the same experimental design but is akin to studying a distinct biological process. We used this process to generate compendia of new gene expression experiments. We then examined the retention of the original differential expression signature by comparing the set of differentially expressed genes (DEGs) found in the simulated experiments (Figure 3D). Applying only the VAE compression to the original experiment (E-GEOD-51409), generated an experiment that had the same sample grouping as the original. However, only a subset of the DEGs found in the VAE compressed experiment were also found in the original experiment. The VAE compression step added some noise to the expression signal in the original experiment, as expected, since the data was being compressed into a low dimensional space. Overall, the correlation between the genes, based on their log2 fold-change values, in the original and VAE compressed experiment was high, R^2 = 0.822 (Figure 3C). Next, ~~W~~we exampled how the original samples in an experiment ~~(E-GEOD- 51409)~~ and a simulated experiment, applying VAE compression and latent space translation of the ~~generated using~~ E-GEOD-51409 experiment ~~as a template~~, ha~~d~~ve consistent clustering of samples (Figure 3D~~C~~ original and experiment~~-~~level simulated experiment).[27] However the sets of genes that were differentially expressed were

Formatted: Font: 11 pt

Formatted: Superscript

different between the two ~~datasets~~experiments. This demonstrated that the perturbation intensity and experimental design were relatively consistent in gene expression space, even though the nature of the perturbation differed. The correlation between genes in the original and the experiment-level experiment was lower, R^2 = 0.230, since it represented a unique experiment. The residual similarity was likely due to commonly differentially expressed genes that have been observed previously[28,29]. Finally, as a control, we demonstrated that the original experiment structure was not well preserved using the random sampling approach (Figure 3D, sample-level simulated experiment). The correlation between genes in the original and sample-level experiment was non-existent, R^2 = -0.055, since we did not account for experiment structure in the sample-level simulation.

Formatted: Superscript

Formatted: Superscript

In general, the numbers of differentially expressed genes found in the experiment-preserving simulated experiments (78 DEGs in VAE compressed, 14 DEGs in experiment-level) were lower compared to the original experiment (505 DEGs). This was because the ~~The~~ simulated experiments had a lower variance compared to the original ~~dataset~~ experiment. This reduced variance was due to the normality assumption made by the VAE, which compresse~~s~~d the latent space data representation.[23] However, ~~in general,~~ the clustering of samples was~~is~~ conserved between the simulated and original experiments~~,~~ and this was also~~as~~ observed in the additional template experiments with more complex ~~structures~~ experimental setups (Figure S4).

Given the fact that we preserved the association between samples and experiments in this new experiment-level simulation, we ~~would~~ expected that ~~new~~ simulated experiments would preserve the correlation in expression of genes that are in the same pathway. In our previous example ~~experiment, E-GEOD-51409~~, the simulated experiment generated using the original E-GEOD-51409 as a template (i.e. experiment-level, Figure 3A) identified 14 ~~differentially expressed genes~~DEGs (Figure 3D~~C~~). In contrast, the simulated experiment generated by random~~ly~~ sampling (i.e. sample-level, Figure 1C) did not identify any ~~differentially expressed genes~~DEGs; the median log2 fold~~-~~change was 0.08. Furthermore, ~~when~~ simulating 100 new experiments using E-GEOD-51409 as a template, ~~the experiments generated using the workflow in Figure 3A~~ identified a median of 2,588 ~~differentially expressed genes~~DEGs compared to ~~those new~~ simulated experiments generated by random~~ly~~ sampling ~~from the compendium resulting from the workflow in Figure 1C (Figure 3D)~~ which identified a median of 0 ~~differentially expressed genes~~DEGs (Figure 3E). Additionally, the median number of enriched KEGG pathways wa~~i~~s 1 using the ~~workflow in Figure 3A~~template shifting approach compared to

0 using the random sampling approach ~~using the previous simulation strategy ~~(Figure 3F~~E~~). Overall, it appeared~~s~~ that this new simulation approach generated a compendium of more realistic experiments with ~~some real ~~underlying biology.~~ and therefore this new simulation represents a more realistic simulation compared to the previous one.~~ (~~E~~examples of the significantly enriched pathways ~~can be seen ~~in Table 2). The top over-represented pathway was~~is~~ the ribosome pathway, which is likely a commonly altered pathway found in many experiments regardless of experiment type, similar to the findings from human array experiments in Crow et. al.[28,29] The remaining pathways found in the original experiment were ~~generally ~~related to metabolism~~ related~~, which is consistent with the finding from the original publication.[27] The simulated experiment was particularly enriched in sulfur metabolism and ABC transporters, which is consistent with an ~~different previous~~ experiment that found upregulation of transport systems in response to sulfate limitations.[30] Overall, in accordance with real gene expression experiments, the new simulated experiments contain related groups of enriched pathways ~~which ~~that reflect the specific hypothese~~i~~s being tested. These results demonstrate the use of a VAE as a hypothesis generating tool.~~;~~ ~~w~~We can now simulate new experiments in order to study the response of *P. aeruginosa* in response to untested conditions~~stimuli~~.

Table 2: *Enriched pathways found in the original E-GEOD-51409 experiment and the pseudo-experiment generated using the experiment-level simulation.*

Original	Adjusted p-value	Experiment level simulation	Adjusted p-value
Pae03010: Ribosome	2.966E-11	Pae03010: Ribosome	7.96E-07
Pae00500: Starch and sucrose metabolism	~~0.001512~~1.512 E-03	Pae02010: ABC transporters	~~0.004009~~4.009 E-03
Pae01200: Carbon metabolism	~~0.004466~~4.466 E-03	Pae00920: Sulfur metabolism	~~0.01576~~1.576E-02
Pae00640: Propanoate metabolism	~~0.001954~~1.954 E-03		

Formatted: Font: (Default) Arial, 10 pt

Formatted: Font: (Default) Arial, 10 pt

Formatted: Font: (Default) Arial, 10 pt

Formatted: Font: (Default) Arial, 10 pt

Simulating gene expression compendia with synthetic experiments

We used our method to simulate new experiments that followed existing patterns to examine the patterns from~~that we observed for~~ generic partitions (Figure 4A). We simulated 600 experiments using the *P. aeruginosa* compendium. We divided these experiments into [1, 2, 3, 5, 10, 20, 30, 50, 70, 100, 200, 300, 400, 500, 600] partitions. These partitions represented groupings of

experiments with shared noise, such as experiments from the same lab or experiments with the same experimental design. Each partition contain<u>ed</u>~~s~~ technical sources of variance within and between experiments. Results with simulated experiments were similar to those from arbitrarily partitioned samples. We observed a monotonic loss of similarity after the correction step as the number of partitions increased (Figure 4B). Visualizing the top principal components revealed that statistical correction initially better recapitulated the overall structure of the data but that ~~with many partitions~~ similarity decreased <u>with many partitions</u> (Figure 4C, dark blue). Without <u>statistical</u> correction there was a larger initial drop in similarity but a later recovery (Figure 4B) and visualizing the top principal components recapitulated this finding (Figure 4C, light blue). We performed analogous experiments using the recount2 VAE and <u>5</u>~~10~~0 simulated experiments <u>with [1, 2, 5, 10, 20, 30, 50] partitions</u>. We observed consistent results with this dataset using both SVCCA similarity (Figure 4D) and visual inspection of the top principal components (Figure 4E).

<u>One caveat in the design of the previous analysis, is that the effect of the number of partitions was confounded by the number of experiments per partition. For example, more partitions equated to each partition having a smaller effect size since each partition had fewer experiments. To study the contribution of individual experiments in our signal detection, we performed an analysis where we held the number of experiments per partition fixed and varied the number of total experiments within a compendia (Figure S5A). With few experiments in a compendia, the main signal was the difference between experiments so adding noise to each experiment drove signal detection down (Figure S5B). Additionally, applying noise correction removed the main experiment-specific signal, as it was designed to do. With more experiments in a compendia, we gained a more global gene expression representation, where the main signal was no longer focused on the difference between experiments. Thus, adding noise to each experiment did not affect our signal detection and our similarity remained constant. However, applying noise correction will consistently remove more of our signal of interest. The results of this analysis exemplify how existing experiments can be combined and used without need for correction.</u>

In summary, as the number of partitions <u>or experiments</u> increase the experiment-specific technical sources contribute less ~~overall~~ to the <u>overall</u> signal and the underlying patterns dominate the overall signal. When many partitions <u>or experiments</u> are present, even ideal statistical approaches to correct for noise over-corrects and removes the underlying signal.

Formatted: Font: (Default) +Body (Calibri), 10 pt, Font color: Auto, Pattern: Clear

Formatted: Comment Text, Font Alignment: Auto

Discussion:

Our findings reveal that compendia-wide analyses do not always require correction for experiment-specific technical variance and that correcting for such variance may remove signal. This simulation study provides an explanation for the observation that past studies[2–6] have successfully extracted biological signatures from gene expression compendia despite the presence of uncorrected experiment-specific sources of technical variability. In general, there exists compendia that contain some small number of experiment-specific sources where traditional correction methods can be effective at recovering the biological structure of interest.; hHowever, there also exist large-scale gene expression compendia where these methods may be harmful instead of helpful. The number of experiment-specific sources that determine whether to apply correction will vary depending on the dataset – the size of the compendia, and the magnitude and structure of the signals. Using the associated repository (https://github.com/greenelab/simulate-expression-compendia) users can customize the scripts to run the simulation experiments on their own expression data in order to examine the effect of a linear noise model with linear noise correction on their dataset. Though our analysis uses simplifying assumptions that preclude us from defining a specific threshold for noise correction, these simulations define a set of general properties that will guide compendia analyses moving forward. This study suggests that new large-scale datasets can be created by distributing different experiments across many different labs and centers as opposed to being consolidated within a single lab.

We introduce a new method to simulate genome-wide gene expression experiments, using existing gene expression data as starting material, which goes beyond simulating individual samples. This allowsed us to examine the extent to which our findings hold with realistic experimental designs. The ability to simulate gene expression experiments with a realistic structure may havehas many potential legitimate uses: e.g., pre-training for machine learning models, providing synthetic test data for software, and other such applications. Additionally, this simulation technique can be used to explore hypothetical experiments that have not been previously performed and generate hypotheses. However, such approaches could also be used by nefarious actors to generate synthetic data for publications. Forensic tools that detect synthetic genome-wide data may be needed to combat potential fraudulent uses.

Our study has several limitations. We assume a certain noise model that differs between experiments. However, the sources of real noise are multifaceted and any such assumption will necessarily be an oversimplification, though such assumptions are not uncommon.[10,12,31] By selecting a specific noise model and using an ideal noise-removal step, we provide a best case scenario for artifact removal. While any simulation study will necessarily make simplifying assumptions, this work is the first to use deep generative models as part of a simulation study to probe the long-standing assumption that correcting for technical variability is necessary for analyses that span multiple experiments. Our findings reveal that in settings with hundreds or thousands of experiments, correcting for experiment-specific effects can harm performance and that it can be best to ~~do nothing~~forgo statistical correction. Adjusting the choices of normalization, noise magnitude, and noise patterns will result in different selections of the precise cross-over point where it becomes beneficial to perform correction. With this design, we do not expect ~~that it is possible~~ to estimate exactly where this precise cross-over point is. Such an estimation~~That~~ would require a compendium where investigators systematically performed the same combination of different experiments in multiple labs at different times. We were unable to identify such a compendium on the scale of thousands of samples from tens to hundreds of labs. Thus, though our analysis necessarily includes simplifying assumptions that limit our ability to precisely define the thresholds for correction for arbitrary datasets and noise sources, it remains suitable for examining the overriding principles that govern compendium-wide analyses.

Formatted: Pattern: Clear (White)

Our study ~~also~~ has broad~~er~~ implications for efforts to standardize scientific processes. Centralization of large-scale data generation has the potential to reduce experiment-specific technical noise, though it comes at a cost of flexibility. Our results suggest that a highly distributed process where experiments are carried out in many different locations, with their own specific sources of technical noise, can also lead to valuable data collections.

Methods:

Pseudomonas aeruginosa gene expression compendium

We downloaded a compendium of *P. aeruginosa* data that was previously used for compendium-wide analyses.[2] Previous studies identified biologically-relevant processes such as oxygen deprivation[2] and phosphate starvation[3] by applying denoising autoencoders. We obtained the processed and normalized gene expression matrices from the ADAGE GitHub repository (https://github.com/greenelab/adage/tree/master/Data_collection_processing). The *P. aeruginosa* dataset was previously processed by Tan et. al.[2] During processing, raw microarray data were downloaded as .cel files, *rma* was used to convert probe intensity values from the *.cel* files to log2 base gene expression measurements, and these gene expression values were then normalized to 0-1 range per genes.

This compendium includes measurements from 107 experiments that contain 989 samples for 5,549 genes.[2] It contains experiments that accrued between the release of the GeneChip *P. aeruginosa* genome array and at the time of data freeze in 2014. Approximately 70% of the samples were from cultures of strain PAO1 and derivatives, 13% were in strain PA14 background, 0.6% were from PAK strains and the remaining were largely clinical isolates. Of the strains, 73% were wild-type (WT) genotypes and the rest were mutants that had undergone genetic modification. Approximately 60% of the samples were grown in lysogeny broth (LB) medium while the rest were grown in Pseudomonas Isolation Agar (PIA), glucose, pyruvate or amino acid-based media.[3] Roughly 80% were grown planktonically, 15% were grown in biofilms and the remaining samples were in vivo or not annotated. Overall, this *P. aeruginosa* compendium covered a wide range of gene expression patterns including: characterization of clinical isolates from cCystic fFibrosis infections, differences between response of mutant versus WT, response ofto antibiotic treatment, microbial interactions, adaptation from water to GI tract infection. Despite having 989 samples, this compendium represents the heterogeneity of *P. aeruginosa* gene expression.

recount2 gene expression compendium

We downloaded human RNA-seq data from recount2.[32] The dataset includes over 70,000 samples collected from Sequencing Read Archive (SRA). It is comprised of more than 50,000 samples from different types of experiments, roughly 10,000 samples from Genotype-Tissue Expression project (GTEx v6) covering 44 types of normal tissue, and more than 10,000 samples from The Cancer Genome Atlas (TCGA) measuring 33 cancer types.[20,33,34] The recount2 authors uniformly processed and quantified these data. We downloaded data using the

recount library in Bioconductor (version 1.14.0).[32] The entire recount2 dataset is 8TB. Based on the *P. aeruginosa* compendium we expected that a subset of the compendium would be sufficient for this simulation, so we selected a random subset of 50 NCBI studies, which resulted in 896 samples with 58,037 genes for our simulation. Each project (imported from NCBI bioproject) is akin to an experiment in the *P. aeruginosa* compendium, and we used the term *experiment* to describe different projects in order to maintain consistency in this paper. The downloaded recount2 dataset was in the form of raw read counts, which was normalized to produce RPKMs used in our analysis. The normalized gene expression data was then scaled to a 0-1 range per gene.

Formatted: Pattern: Clear

~~Constructing a generative model of gene expression compendia~~

Strategy to construct VAE: structure and hyperparameters

Formatted: Font: Italic

We designed an approach to simulate gene expression compendia with a multi-layer variational autoencoder (VAE). We built this model in Keras (version 2.1.6) with a TensorFlow backend (version 1.10.0), modifying the previously published Tybalt method.[35–37] Our architecture used each input gene as a feature. These genes were compressed to 2,500 intermediate features using a rectified linear unit (ReLU) activation function to combine weighted nodes from the previous layer. These features were encoded into 30 latent space features, also using a ReLU activation function, which were optimized via the addition of a Kullbach-Leibler (KL) divergence term into the loss function (binary cross entropy) to follow a standard normal distribution. These features were then reconstructed back to the input feature dimensions using decoding layers that mirror the structure of the encoder network. We trained the VAE using 90% of the input dataset, leaving 10% as a validation set. We determined training hyperparameters by manually adjusting parameters and selecting the parameters that optimized the validation loss based on visual inspection. These were a learning rate of 0.001, a batch size of 100, warmups set to 0.01, 100 epochs for the *P. aeruginosa* compendium and 20 epochs for the recount2 compendium. A similar assessment was performed to determine the neural network architecture. We manually inspected the validation loss using multiple different 2-layer designs (300-10, 2500-10, 2500-20, 2500-30, 2500-100, 2500-300) and found a 2,500 layer to a 30 hidden layer VAE to be most optimal.

~~Simulating gene expression compendia~~Sample-based simulation

We used the VAE trained from each compendium to generate new compendia by randomly sampling from the latent space. We generated a simulated compendium containing 6,000 *P. aeruginosa* samples or 500 recount2 samples. For our first simulation, we sampled randomly - ignoring the relationship between samples within a specific experiment. We simulated experiment-specific sources of undesired variability within compendia by dividing the data into partitions and adding noise to each partition.

We divided the *P. aeruginosa* simulated compendium into [1, 2, 5, 10, 20, 50, 100, 500, 1000, 2000, 3000, 6000] partitions and divided the recount2 simulated compendium into [1, 2, 5, 10, 20, 50, 100, 250, 500] partitions. Each partition of data representeds a group of samples that are from the same experiment or lab. We randomly added linear noise to each partition by generating a vector of length equal to the number of genes (5,549 *P. aeruginosa* genes and 58,037 hHuman genes) where each value in the vector was drawn from a normal distribution with a mean of 0 and a variance of 0.2. With the 0-1 scaling, a value of 0.2 produces a relatively large difference in gene expression space (Figure S1).

Though linear noise is an over-simplification of the types of noise that affect gene expression data, it allowed us to design an approach to optimally remove noise. Adjusting the choices of normalization, noise magnitude, and noise patterns will result in different selections of the precise cross-over point where it becomes beneficial to perform correction. With this design, we do not expect that it is possible to estimate exactly where this precise cross-over point is. That would require a compendium where investigators systematically performed the same combination of different experiments in multiple labs at different times. We were unable to identify such a compendium on the scale of thousands of samples from tens to hundreds of labs. Thus, though our analysis necessarily includes simplifying assumptions that limit our ability to precisely define the thresholds for correction for arbitrary datasets and noise sources, it remains suitable for examining the overriding principles that govern compendium-wide analyses.

Experiment-based simulation

For the experiment-level simulation, we developed an approach that could simulate realistic experimental structure. There was no consistent set of annotated experimental designs, so we developed a simulation method that did not depend on *a priori* knowledge of experimental

design. For each synthetic experiment, we randomly sampled a “template experiment” from the set of *P. aeruginosa* or recount2 experiments. We then simulated new data that matched the template experiment by selecting a random location from the low dimensional representation of the simulated compendia (i.e. selecting a location according to the low dimensional distribution) and calculating the vector that connected this random location and the encoded template experiment. We then linearly shifted the template experiment in the low-dimensional latent space by adding this vector to each sample in the experiment. This process preserved the relationship between samples within the experiment but shifted the samples to a new location in the latent space. Repeating this process for each experiment allowed us to generate new simulated compendia comprised of realistic experimental designs.

We divided the *P. aeruginosa* simulated compendium into [1, 2, 3, 5, 10, 20, 30, 50, 70, 100, 200, 300, 400, 500, 600] partitions and divided the recount2 simulated compendium into [1, 2, 5, 10, 20, 30, 50] partitions, where experiments are divided equally amongst the partitions. For each partition we added simulated noise as described in the previous section. Experiments within the same partition had the same noise added. Each partition represented a group of experiments generated from the same lab or with the same experimental design.

Experiment-effect analysis

For this analysis we wanted to examine the effect of individual experiments in our ability to detect underlying gene expression structure. First, we used the experiment-based simulation approached to simulate *P. aeruginosa* compendia with [2, 3, 5, 10, 20, 30, 50, 70, 100, 200, 300, 400, 500, 600] experiments. Next, we divided the simulated compendium into the same number of partitions so that there was one experiment per partition. For each partition we added simulated noise as described in the previous section. Finally we used SVCCA to compare the noisy compendia with X number experiments with the unpartitioned compendia with X number of experiments. We also used SVCCA to compare the noise-corrected compendia with X experiments with the unpartitioned compendia with X experiments.

Removing technical variability from noisy compendia

Our model of undesired variability was~~is~~ a linear signature applied separately to each partition of the data, which we considered akin to experiments or groups of experiments in a compendium of gene expression data. We used the removeBatchEffect function in the R library, limma (version 3.44.0), to correct for the technical variation that was artificially added to the simulated compendia.[25] Limma removes the technical noise by first fitting a linear model to describe the relationship between the input gene expression data and the experiment labels. The input expression data contains both a biological signal and technical noise component. By fitting a linear model, limma will extract the noise contribution and then subtract this from the total input expression data. This method presents a best-case scenario for removing the undesired variability in the simulated compendia because the model matches the noise pattern we~~'ve~~ used in the simulation.

~~*Simulating experiments that comprise gene expression compendia*~~

~~For the next simulation, we developed an approach that could simulate realistic experimental structure. We next generated synthetic experiments for which the gene expression patterns were consistent with the types of experiments that are used within the field. There was no consistent set of annotated experimental designs, so we developed a simulation method that did not depend on *a priori* knowledge of experimental design. For each synthetic experiment, we randomly sampled a "template experiment" from the set of *P. aeruginosa* or recount2 experiments. We then simulated new data that matched the template experiment by selecting a random sample from the low dimensional representation of the simulated compendia and calculating the vector that connects this random sample and the encoded template experiment. We then linearly shifted the template experiment in the low-dimensional latent space by adding this vector to each sample in the experiment. This process preserves the relationship between samples within the experiment but shifts the samples to a new location in the latent space. Intuitively this simulation maintains the same experimental design but is akin to studying a distinct biological process. Repeating this process for each experiment allowed us to generate new simulated compendia comprised of realistic experimental designs.~~

~~We divided the *P. aeruginosa* simulated compendium into [1, 2, 3, 5, 10, 20, 30, 50, 70, 100, 200, 300, 400, 500, 600] partitions and divided the recount2 simulated compendium into [1, 2, 5, 10, 20, 30, 50] partitions, where experiments are divided equally amongst the partitions. For each partition we added simulated noise as described in the previous section. Experiments~~

within the same partition have the same noise added. Each partition represents a group of experiments generated from the same lab or with the same experimental design.

Measuring the similarity of matched compendia

We used Singular Vector Canonical Correlation Analysis (SVCCA)[26] to estimate similarities between different compendia. SVCCA is a method designed to compare two data representations[26]. Given two multivariate datasets, X_1 and X_2, the goal of SVCCA is to find the basis vectors, *w* and *s*, to maximize the correlation between w^TX^1 and s^TX_2. In other words, SVCCA attempts to find the space, defined by a set of basis vectors, such that the projection of the data onto that space is most correlated. Two datasets are considered similar if their linearly invariant correlation is high (i.e., if X_1 is a shift or rotation of X_2 then X_1 and X_2 are considered similar).

We compared the statistical structure of the gene expression, projected onto the first 10 principle components, in the baseline simulated compendia (those with only one experiment or partition, X_1) versus those with multiple experiments or partitions (X_2). Our SVCCA analysis was designed to measure the extent to which the gene expression structure of the compendia without noise iswas similar to the gene expression structure of the compendia with multiple sources of technical variance has been added as well as those where correction has been applied. Here we use 10 principle components for computational simplicity. Selecting a different value would affect the crossover point but not the general trends that we describe

Formatted: Pattern: Clear

A case study of differential expression in a template experiment

We compared the E-GEOD-51409 experiment[38] with two different simulated representations to provide a case study for experiment-based simulation. E-GEOD-51409 included *P. aeruginosa* in two different growth conditions. For one simulation, we generated random samples and randomly assigned them to conditions, which we termed the sample-simulated experiment. For the second we used the latent space transformation process described above, which we termed the experiment-simulated experiment. We used the eBayes module in the limma library to calculate differential gene expression values for each gene between the two different growth conditions in the real and simulated data. We built heatmaps for the 14 most differentially expressed genes, where differentially expressed genes where those with FDR adjusted cutoff

(using Benjamini-Hochberg correction) < 0.05 and log2 fold~~-~~change >1, which are thresholds frequently used in practice. We selected 14 genes because there were 505, 14 and~~,~~ 0 differentially expressed genes found in the original experiment, experiment-simulated experiment~~,~~ and sample-simulated experiment, respectively. Since there were 0 differentially expressed genes found in the sample-simulated experiment, we displayed the top 14 genes sorted by adjusted p-value to provide a visual summary of the simulation process.

Comparing sample-level and experiment-level simulated datasets

We simulated 100 experiments using the template E-GEOD-51409 experiment[38]. We sought to compare the sample-level and experiment-level simulation processes. We set a threshold for differentially expressed genes at a Bonferroni-corrected p-value cutoff of 0.05/5549. We used the enrichKEGG module in the clusterProfiler library to conduct an over-representation analysis[39]. We used the Fisher's exact test to calculate a p-value for over-representation of pathways in the set of differentially expressed genes. We considered pathways to be over-represented if the ~~Bonferroni corrected p~~q-value was less than 0.02~~5~~.

Implementation and Software Availability

All scripts to reproduce this analysis are available the GitHub repository (https://github.com/greenelab/simulate-expression-compendia) under an open source license. The repository contains 98% python jupyter notebooks, 2% python and 0.1% R scripts. The repository's structure is separated by input dataset. Pseudomonas/ and Human/ directories each contain the input data in the data/input/ directory. Scripts for the sample level simulation can be found in Pseudomonas /Pseudomonas_sample_lvl_sim.ipynb for the *P. aeruginosa* compendium and Human/Human_sample_lvl_sim.ipynb for the recount2 compendium. Scripts for the experiment level simulation can be found in Pseudomonas/Pseudomonas_experiment_lvl_sim.ipynb and Human/Human_experiment_lvl_sim.ipynb respectively. The virtual environment was managed using conda (version 4.6.12), and the required libraries and packages are defined in the environment.yml file. Additionally, scripts to simulate gene expression compendia using the sample-level and experiment-level approaches are available as a separate module, called *ponyo*, and can be installed from PyPi (https://github.com/greenelab/ponyo). We describe in the

Formatted: Font: 11 pt

Formatted: Font: (Default) Arial, 11 pt

Formatted: Font: (Default) Arial, 11 pt

Readme file how users can analyze different compendia or use different noise patterns. All simulations were run on a CPU.

Figure Legends:

Figure 1. Simulating gene expression data using VAE. A) Architecture of the VAE, where the input data gets compressed into intermediate layer of 2500 features and then into a hidden layer of 30 latent features. Each latent feature follows a normal distribution with mean µ and variance σ. The input dimensions of the ~~Pseudomonas~~ *P. aeruginosa* dataset are shown here as an example (989 samples, 5549 genes). The same architecture is used to train the recount2 dataset except the input has 896 samples and 58,037 genes. B) Validation loss plotted per epoch during training using the P. aeruginosa compendium. C) Workflow to simulate gene expression samples from a compendium model, where new samples are generated by sampling from the latent space distribution. D) UMAP projection of P. aeruginosa gene expression data from the real dataset (pink) and the simulated compendium using the workflow in C (grey).

Formatted: Font: Italic

Figure 2. Results of simulating compendia. A) workflow describing how experiment-specific noise was added to the simulated compendia and how the noisy simulated compendia were evaluated for similarity compared to the ~~original input~~unpartitioned simulated compendia. B,D) SVCCA curve measuring the similarity between a compendia without noise versus a compendium with noise (light blue), compendium with noise corrected for (dark blue). As a negative control, we used the similarity between the gene expression pattern of the simulated data with a single partition compared with the simulated data that has been permuted to destroy any meaningful structure in the data. C,E) Subsampled gene expression data (500 samples per compendia) projected onto the first two principal components showing the overlap in structure between the compendia without noise (gray) versus the compendia with noise (light blue), compendia with noise corrected for (dark blue).

Figure 3. Simulating gene expression compendia by experiment. A) Workflow to simulate gene expression per experiment. B) UMAP projection of P. aeruginosa gene expression data highlighting a single experiment, E-GEOD-51409, (red) in the original dataset (left) and the simulated dataset (right), which was subsampled to 1000 samples. C) Differential expression analysis of experiment E-GEOD-51409 (left), random simulated samples (middle), simulated samples using the same experiment as a template (right). D) Number of differentially expressed genes identified across 100 simulated experiments generated using experiment-level simulation and sample-level simulation. E) Number of enriched pathways identified across 100 simulated experiments generated using experiment-level simulation and sample-level simulation.

Figure 4. Results of simulating compendia comprised of gene expression experiments. A) workflow describing how experiment-specific noise was added to the simulated compendia and how the noisy simulated compendia were evaluated for similarity compared to the ~~original input~~unpartitioned simulated compendia. B,D) SVCCA curve measuring the similarity between a compendia without noise versus a compendium with noise (light blue), compendium with noise corrected for (dark blue). As a negative control, we used the similarity between the gene expression pattern of the simulated data with a single partition compared with the simulated data ~~that has been~~ permuted to destroy any meaningful structure in the data. C,E) Subsampled gene expression data (500 samples per compendia) projected onto the first two principal components showing the overlap in structure between the compendia without noise (gray) versus the compendia with noise (light blue), compendia with noise corrected for (dark blue).

~~**Figure S1**. Results of varying the magnitude of the experiment-specific noise added to each partition. SVCCA curve measuring the similarity between a compendia without noise versus a compendium with noise (light blue), compendium with noise corrected for (dark blue). As a negative control, we used the similarity between the gene expression pattern of the simulated data with a single partition compared with the simulated data that has been permuted to destroy any meaningful structure in the data. Using noise model with A) 0.2 variance, B) 0.05 variance with a zoomed in view on the left, C) 0.025 variance with a zoomed in view on the left.~~

Figure S1~~2~~. Simulating recount2 gene expression data using VAE. A) Validation loss plotted per epoch during training. B) UMAP projection of gene expression data from the real dataset (pink) and the simulated compendium using the workflow in Figure 1C (grey).

Figure S2~~1~~. Results of varying the magnitude of the experiment-specific noise added to each partition. SVCCA curve measuring the similarity between a compendia without noise versus a compendium with noise (light blue), compendium with noise corrected for (dark blue). As a negative control, we used the similarity between the gene expression pattern of the simulated data with a single partition compared with the simulated data ~~that has been~~ permuted to destroy any meaningful structure in the data. Using noise model with A) 0.2 variance, B) 0.05 variance with a zoomed in view on the left, C) 0.025 variance with a zoomed in view on the left.

Formatted: Caption, Line spacing: 1.5 lines

Formatted: Font: (Default) +Body (Calibri), Italic

Formatted: Normal, Line spacing: single

Figure S3. Results of simulating *P. aeruginosa* compendia using A) sample-level simulation or B) experiment-level simulation with COMBAT noise correction.

Figure S4. Clustering of 100 random gene expression profiles in original versus simulated experiments using A) E-GEOD-21704 and B) E-GEOD-10030 as templated.

Figure S5. Results of simulating compendia with fixed number of experiments. A) workflow describing how each compendia is designed to have a fixed number of experiments, experiment-specific noise was added to the simulated compendia and how the noisy simulated compendia were evaluated for similarity compared to the unpartitioned simulated compendia. B) SVCCA curve measuring the similarity between a compendia without noise versus a compendium with noise (light blue), compendium with noise corrected for (dark blue). As a negative control, we used the similarity between the gene expression pattern of the simulated data with a single partition compared with the simulated data that has been permuted to destroy any meaningful structure in the data. C) Subsampled gene expression data (fewer than 500 samples per compendia) projected onto the first two principal components showing the overlap in structure between the compendia without noise (gray) versus the compendia with noise (light blue), compendia with noise corrected for (dark blue).

Formatted: Font: (Default) +Body (Calibri), Not Bold, Italic, Check spelling and grammar

Formatted: Normal, Line spacing: single

References:

1. Perou CM. Show me the data! *Nat Genet*. 2001;29(4):373. doi:10.1038/ng1201-373
2. Tan J, Hammond JH, Hogan DA, Greene CS. ADAGE-Based Integration of Publicly Available Pseudomonas aeruginosa Gene Expression Data with Denoising Autoencoders Illuminates Microbe-Host Interactions. *mSystems*. 2016;1(1). doi:10.1128/mSystems.00025-15
3. Tan J, Doing G, Lewis KA, et al. Unsupervised Extraction of Stable Expression Signatures from Public Compendia with an Ensemble of Neural Networks. *Cell Syst*. 2017;5(1):63-71.e6. doi:10.1016/j.cels.2017.06.003
4. Chen L, Cai C, Chen V, Lu X. Learning a hierarchical representation of the yeast transcriptomic machinery using an autoencoder model. *BMC Bioinformatics*. 2016;17 Suppl 1(Suppl 1):9. doi:10.1186/s12859-015-0852-1
5. Zhou W, Altman RB. Data-driven human transcriptomic modules determined by independent component analysis. *BMC Bioinformatics*. 2018;19(1):327. doi:10.1186/s12859-018-2338-4
6. Taroni JN, Grayson PC, Hu Q, et al. MultiPLIER: a transfer learning framework for transcriptomics reveals systemic features of rare disease. *Cell Syst*. 2019;8(5):380-394.
7. Leek JT, Storey JD. Capturing heterogeneity in gene expression studies by surrogate variable analysis. *PLoS Genet*. 2007;3(9):1724-1735. doi:10.1371/journal.pgen.0030161
8. Renard E, Absil PA. Comparison of batch effect removal methods in the presence of correlation between outcome and batch. 2017.
9. Tseng GC, Oh M-K, Rohlin L, Liao JC, Wong WH. Issues in cDNA microarray analysis: quality filtering, channel normalization, models of variations and assessment of gene effects. *Nucleic Acids Res*. 2001;29(12):2549-2557. doi:10.1093/nar/29.12.2549
10. Kerr MK, Martin M, Churchill GA. Analysis of variance for gene expression microarray data. *J Comput Biol*. 2000;7(6):819-837.
11. Chen C, Grennan K, Badner J, et al. Removing batch effects in analysis of expression microarray data: an evaluation of six batch adjustment methods. *PLoS One*. 2011;6(2):e17238-e17238. doi:10.1371/journal.pone.0017238
12. Johnson WE, Li C, Rabinovic A. Adjusting batch effects in microarray expression data using empirical Bayes methods. *Biostatistics*. 2007;8(1):118-127. doi:10.1093/biostatistics/kxj037
13. Stegle O, Parts L, Piipari M, Winn J, Durbin R. Using probabilistic estimation of expression residuals (PEER) to obtain increased power and interpretability of gene expression analyses. *Nat Protoc*. 2012;7(3):500.
14. Taroni JN, Greene CS. Cross-platform normalization enables machine learning model training on microarray and RNA-Seq data simultaneously. *bioRxiv*. January 2017:118349. doi:10.1101/118349
15. Parrish RS, Spencer III HJ, Xu P. Distribution modeling and simulation of gene expression data. *Comput Stat Data Anal*. 2009;53(5):1650-1660.
16. Singhal S, Kyvernitis CG, Johnson SW, Kaiser LR, Liebman MN, Albelda SM. Microarray data simulator for improved selection of differentially expressed genes. *Cancer Biol Ther*. 2003;2(4):383-391.
17. Law CW, Chen Y, Shi W, Smyth GK. voom: Precision weights unlock linear model analysis tools for RNA-seq read counts. *Genome Biol*. 2014;15(2):R29.
18. Pimentel H, Bray NL, Puente S, Melsted P, Pachter L. Differential analysis of RNA-seq incorporating quantification uncertainty. *Nat Methods*. 2017;14(7):687.
19. Casey S. Greene, Dongbo Hu, Richard W. W. Jones, Stephanie Liu, David S. Mejia, Rob Patro, Stephen R. Piccolo, Ariel Rodriguez Romero, Hirak Sarkar, Candace L. Savonen, Jaclyn N. Taroni, William E. Vauclain, Deepashree Venkatesh Prasad KGW. refine.bio: a resource of uniformly processed publicly available gene expression datasets.

20. Leinonen R, Sugawara H, Shumway M, Collaboration INSD. The sequence read archive. *Nucleic Acids Res*. 2010;39(suppl_1):D19-D21.
21. Edgar R, Domrachev M, Lash AE. Gene Expression Omnibus: NCBI gene expression and hybridization array data repository. *Nucleic Acids Res*. 2002;30(1):207-210.
22. Brazma A, Parkinson H, Sarkans U, et al. ArrayExpress—a public repository for microarray gene expression data at the EBI. *Nucleic Acids Res*. 2003;31(1):68-71.
23. Kingma DP, Welling M. Auto-encoding variational bayes. *arXiv Prepr arXiv13126114*. 2013.
24. McInnes L, Healy J, Melville J. Umap: Uniform manifold approximation and projection for dimension reduction. *arXiv Prepr arXiv180203426*. 2018.
25. Ritchie ME, Phipson B, Wu D, et al. limma powers differential expression analyses for RNA-sequencing and microarray studies. *Nucleic Acids Res*. 2015;43(7):e47-e47.
26. Raghu M, Gilmer J, Yosinski J, Sohl-Dickstein J. Svcca: Singular vector canonical correlation analysis for deep learning dynamics and interpretability. In: *Advances in Neural Information Processing Systems*. ; 2017:6076-6085.
27. Barbier M, Damron FH, Bielecki P, et al. From the environment to the host: re-wiring of the transcriptome of Pseudomonas aeruginosa from 22°C to 37°C. *PLoS One*. 2014;9(2):e89941-e89941. doi:10.1371/journal.pone.0089941
28. Powers RK, Goodspeed A, Pielke-Lombardo H, Tan A-C, Costello JC. GSEA-InContext: identifying novel and common patterns in expression experiments. *Bioinformatics*. 2018;34(13):i555-i564. doi:10.1093/bioinformatics/bty271
29. Crow M, Lim N, Ballouz S, Pavlidis P, Gillis J. Predictability of human differential gene expression. *Proc Natl Acad Sci*. 2019;116(13):6491 LP - 6500. doi:10.1073/pnas.1802973116
30. Tralau T, Vuilleumier S, Thibault C, Campbell BJ, Hart CA, Kertesz MA. Transcriptomic analysis of the sulfate starvation response of Pseudomonas aeruginosa. *J Bacteriol*. 2007;189(19):6743-6750.
31. Espín-Pérez A, Portier C, Chadeau-Hyam M, van Veldhoven K, Kleinjans JCS, de Kok TMCM. Comparison of statistical methods and the use of quality control samples for batch effect correction in human transcriptome data. *PLoS One*. 2018;13(8):e0202947-e0202947. doi:10.1371/journal.pone.0202947
32. Collado-Torres L, Nellore A, Kammers K, et al. Reproducible RNA-seq analysis using recount2. *Nat Biotechnol*. 2017;35(4):319-321. doi:10.1038/nbt.3838
33. Weinstein JN, Collisson EA, Mills GB, et al. The Cancer Genome Atlas Pan-Cancer analysis project. *Nat Genet*. 2013;45(10):1113-1120. doi:10.1038/ng.2764
34. Consortium Gte. The Genotype-Tissue Expression (GTEx) pilot analysis: multitissue gene regulation in humans. *Science (80-)*. 2015;348(6235):648-660.
35. Abadi M, Agarwal A, Barham P, et al. Tensorflow: Large-scale machine learning on heterogeneous distributed systems. *arXiv Prepr arXiv160304467*. 2016.
36. Chollet F. No Title.
37. Way GP, Greene CS. Extracting a biologically relevant latent space from cancer transcriptomes with variational autoencoders. *Pac Symp Biocomput*. 2018;23:80-91.
38. Smyth Gordon K. Linear models and empirical bayes methods for assessing differential expression in microarray experiments. *Stat Appl Genet Mol Biol*. 2004;3(1):1-25.
39. Yu G, Wang L-G, Han Y, He Q-Y. clusterProfiler: an R package for comparing biological themes among gene clusters. *Omi a J Integr Biol*. 2012;16(5):284-287.
