# Supplementary figures and images for: Correcting for experiment-specific variability in expression compendia can remove underlying signals
Source: Gigascience. 2020 Nov 3;9(11):giaa117. doi: 10.1093/gigascience/giaa117 (PMC7607552; doi:10.1093/gigascience/giaa117)

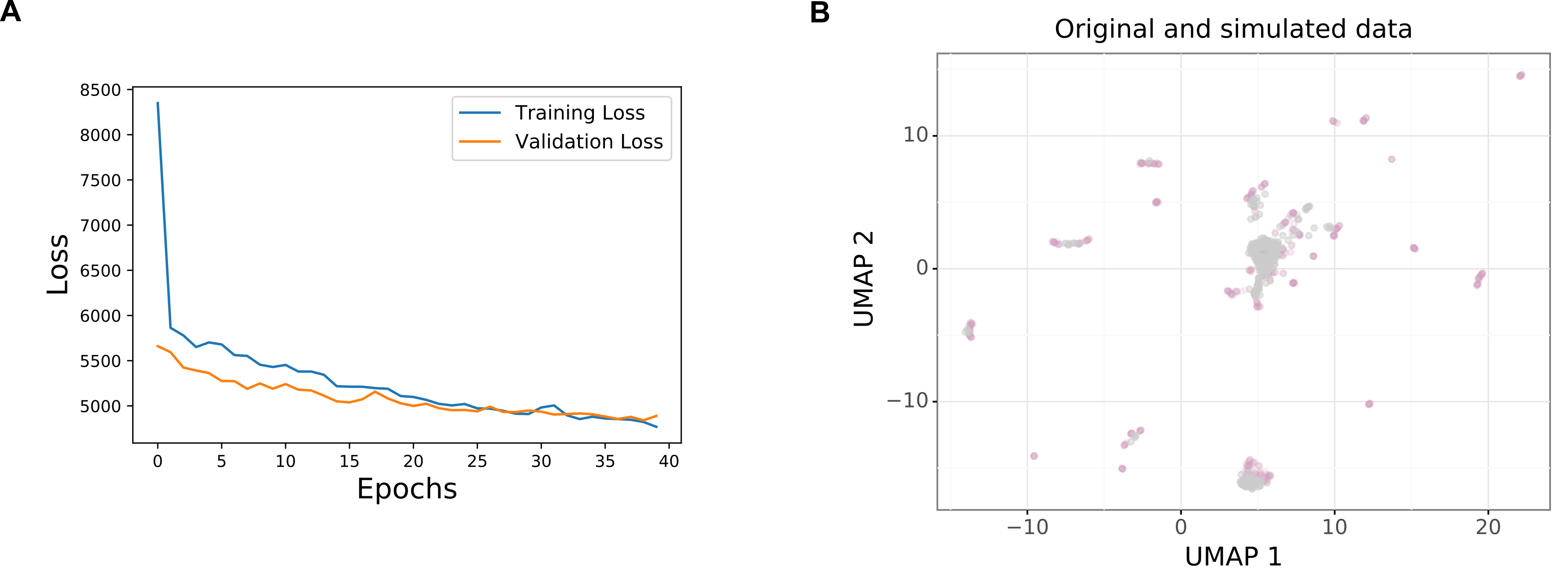

Supplement: giaa117_Supplemental_Figures [file giaa117_supplemental_figures.zip › Supp1.png]

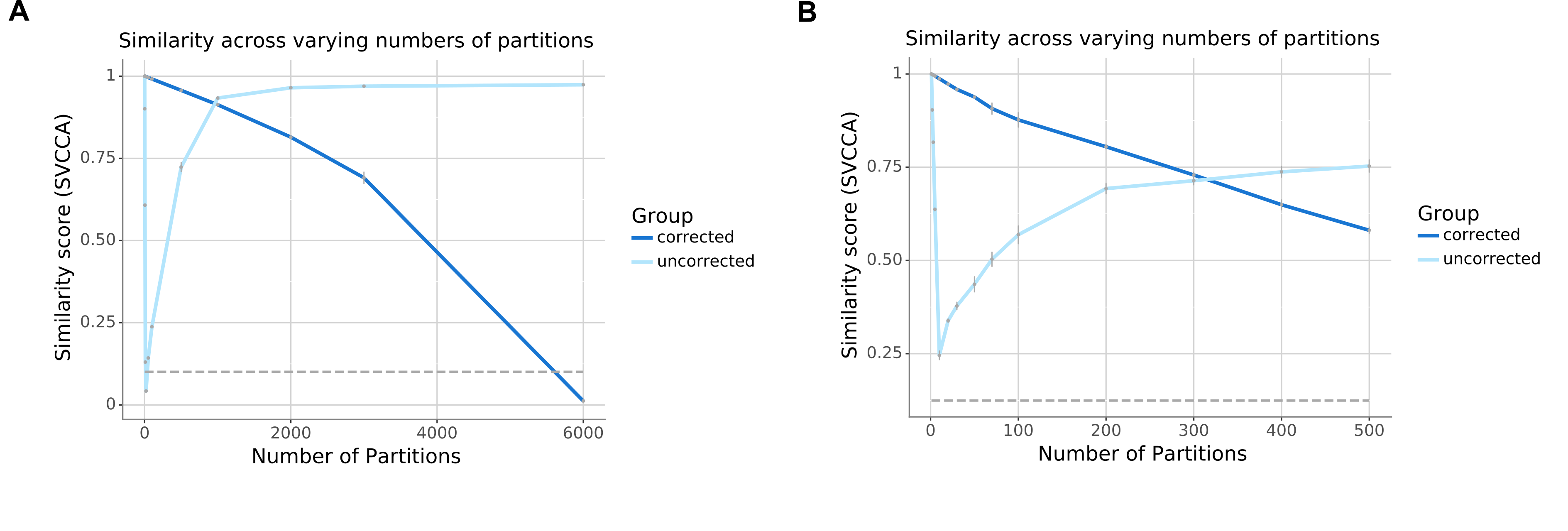

Supplement: giaa117_Supplemental_Figures [file giaa117_supplemental_figures.zip › Supp3.png]

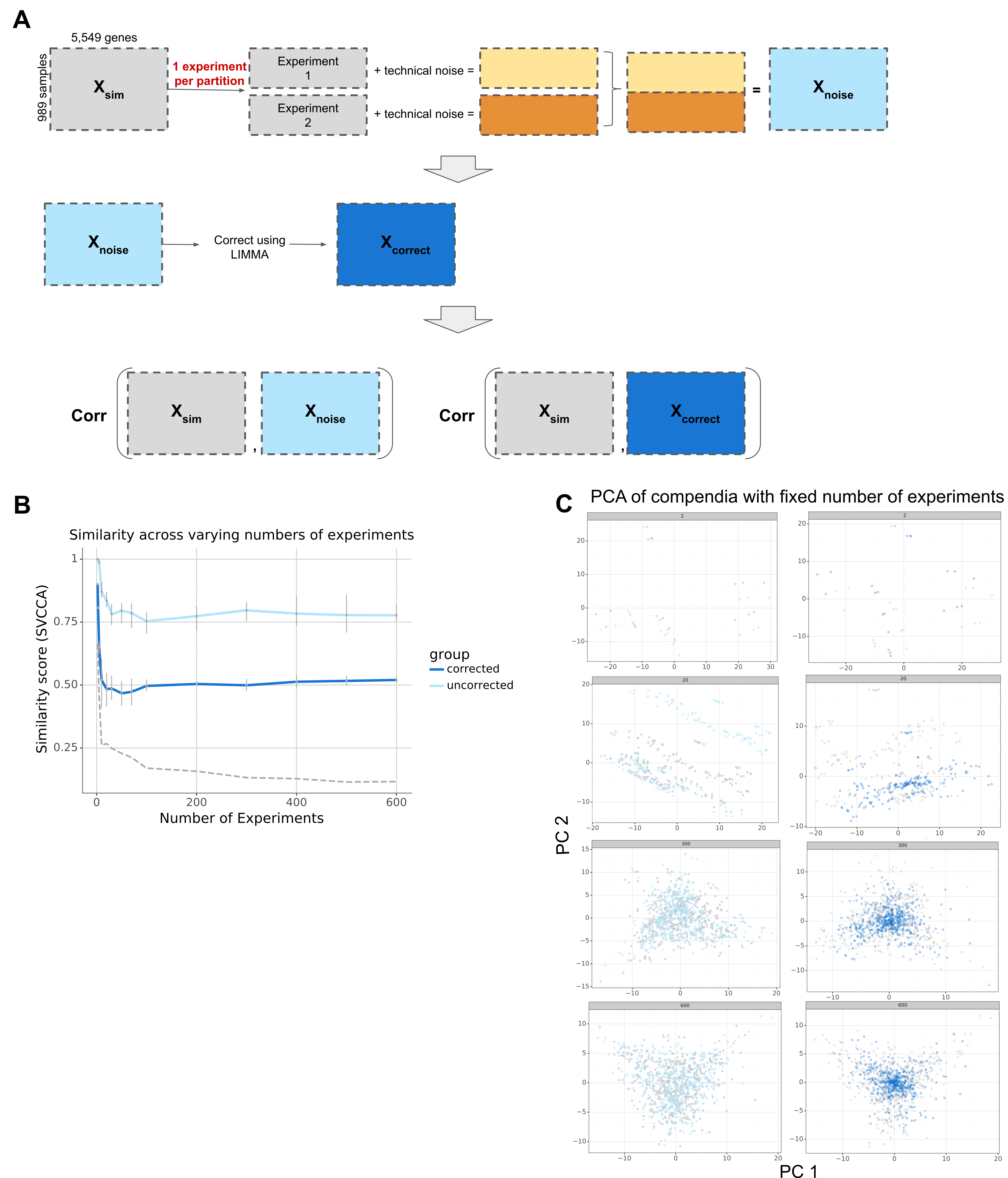

Supplement: giaa117_Supplemental_Figures [file giaa117_supplemental_figures.zip › Supp5.png]
