# Supplementary material for: Correcting for experiment-specific variability in expression compendia can remove underlying signals
Source: Gigascience. 2020 Nov 3;9(11):giaa117. doi: 10.1093/gigascience/giaa117 (PMC7607552; doi:10.1093/gigascience/giaa117)
Supplement: giaa117_Response_to_Reviewer_Comments_Original_Submission [file giaa117_response_to_reviewer_comments_original_submission.pdf]

Author's Response To Reviewer Comments

Close

We uploaded the response to reviewer as a cover letter because we embedded figures to provide clarity and a self-contained document.

Close
