# Supplementary material for: Correcting for experiment-specific variability in expression compendia can remove underlying signals
Source: Gigascience. 2020 Nov 3;9(11):giaa117. doi: 10.1093/gigascience/giaa117 (PMC7607552; doi:10.1093/gigascience/giaa117)
Supplement: giaa117_Reviewer_2_Report_Original_Submission — Eric Lu Zhang -- 7/31/2020 Reviewed [file giaa117_reviewer_2_report_original_submission.pdf]

Reviewer Report

Title: Correcting for experiment-specific variability in expression compendia can remove underlying signals

Version: Original Submission **Date:** 7/31/2020

Reviewer name: Eric Lu Zhang

Reviewer Comments to Author:

Alexandra et al. and colleagues investigated the key problem for correcting experiment-specific bias in large gene expression compendia by developing a VAE-based framework. They performed several experiments on both microarray (from P. aeruginosa) and RNA-seq data (from human tissues) for three different simulation scenarios, for sample-based simulation, single experiment-based simulation and simulations for synthetic experiments. The authors observed the correction of experiment-specific signals could also remove biological signals if the partition size is large. They also provided an open-source package to allow the users to examine their own data. With the prevalence of compendium-based studies, the results from this paper could become a practical guideline. Before publication, there are several issues need to be addressed first. Especially for the experiments, I think they should be closer to the real practical applications.

1. In gene expression analysis, the objective is commonly to seek for differentially expressed genes and gene co-expression networks. The experiments should be designed to investigate these relevant signals rather than only compare the structure with original data. In single experiment-based simulation, the paper had some work on differentially expressed genes and enriched pathways in Figure 3C and Table 2, but the results look a little bit weird. There were 505 and 14 differentially expressed genes found in the original experiment and experiment-simulated data, where many signals were removed in VAE-based simulation. Also, in Figure 3C, I found the top differentially expressed genes for original data and experiment-level simulation data are quite different. The author concluded "Repeating this process for each experiment allowed us to generate new simulated compendia comprised of realistic experimental designs.", but I don't think it is correct if their differentially expressed genes are not from the same pool. For simulating multiple synthetic experiments, I do suggest to add another set of experiments to evaluate the similarity between corrected and uncorrected data by calculating the proportion of differentially expressed genes or strong gene co-expression connections from original data can be captured. E.g. collecting several experiments from the same disease with both patients and controls. I think it is a more practical scenario in biomedical research.

2. With a large number of partitions, I am not surprised the correlation is low between the original data and corrected simulated data. Besides the number of partitions, the sample size per partition is another factor influence batch effect correction. E.g. Based on the information from Table 1, if we assume the no. samples per partition is 10 and one partition corresponding to one experiment, before the partition ca. 350 (in Figure 2D), the corrected data is better than the uncorrected one. We have the experiments by fixing sample size and changing the partition number. And I wonder if the no. samples per partition is fixed (e.g 10), what happens when the number of partition increases? Also, the conclusion should be

more precise with the information for both number of partition and sample size.
3. Most parts of this paper are well-written, but the structure of several long sentences should be further revised.
Minor points
1. For the VAE model, how to determine the number of hidden layers and hidden nodes? Why you choose 30 latent space features finally? Do the users need GPU to run the simulation codes?
2. Page 5, "We evaluated the training and validation set loss at each epoch, which stabilized after roughly 100 epochs (Figure 1B)". Although the authors mentioned the dataset they used in Figure legend, but not in the text.
3. Page 7, "We used UMAP to visualize the structure of the original and data…", data refers to simulated data?
4. The authors should involve some key numbers in the result section, such as the number of simulated samples, the list of number of partitions etc.
5. In Methods section, delete "The P. aeruginosa dataset was previously processed by Tan et. al.2 ", this have been mentioned two times before.
6. Page 13, "58,037 gene". This is not for "gene", using "transcript" is more precise
7. Page14. I suggest to divide the "Constructing a generative model of gene expression compendia" into three sections, 1. The strategy to construct VAE and define its structure and hyperparameters, 2. Sample-based simulation; 3. Experiment-based simulation
8. Page 14, move "Though linear noise is an over-simplification of the types…" to discussion.
9. Page 9. "We exampled how the original samples in an experiment (E-GEOD- 51409)…. However the genes that were differently expressed were different between the two datasets." This sentence should be revised.

Methods

Are the methods appropriate to the aims of the study, are they well described, and are necessary controls included? Choose an item.

Conclusions

Are the conclusions adequately supported by the data shown? Choose an item.

Reporting Standards

Does the manuscript adhere to the journal’s guidelines on [minimum standards of reporting?](#) Choose an item.

Choose an item.

Statistics

Are you able to assess all statistics in the manuscript, including the appropriateness of statistical tests used? Choose an item.

Quality of Written English

Please indicate the quality of language in the manuscript: Choose an item.

Declaration of Competing Interests

Please complete a declaration of competing interests, considering the following questions:

- Have you in the past five years received reimbursements, fees, funding, or salary from an organisation that may in any way gain or lose financially from the publication of this manuscript, either now or in the future?
- Do you hold any stocks or shares in an organisation that may in any way gain or lose financially from the publication of this manuscript, either now or in the future?
- Do you hold or are you currently applying for any patents relating to the content of the manuscript?
- Have you received reimbursements, fees, funding, or salary from an organization that holds or has applied for patents relating to the content of the manuscript?
- Do you have any other financial competing interests?
- Do you have any non-financial competing interests in relation to this paper?

If you can answer no to all of the above, write 'I declare that I have no competing interests' below. If your reply is yes to any, please give details below.

I declare that I have no competing interests.

I agree to the open peer review policy of the journal. I understand that my name will be included on my report to the authors and, if the manuscript is accepted for publication, my named report including any attachments I upload will be posted on the website along with the authors' responses. I agree for my report to be made available under an Open Access Creative Commons CC-BY license (http://creativecommons.org/licenses/by/4.0/). I understand that any comments which I do not wish to be included in my named report can be included as confidential comments to the editors, which will not be published.

Choose an item.

To further support our reviewers, we have joined with Publons, where you can gain additional credit to further highlight your hard work (see: https://publons.com/journal/530/gigascience). On publication of this paper, your review will be automatically added to Publons, you can then choose whether or not to claim your Publons credit. I understand this statement.

Yes Choose an item.
